# 12,13-diHOME Promotes Inflammatory Macrophages and Epigenetically Modifies Their Capacity to Respond to Microbes and Allergens

**DOI:** 10.1155/2024/2506586

**Published:** 2024-06-29

**Authors:** Din L. Lin, Kevin M. Magnaye, Cara E. Porsche, Sophia R. Levan, Elze Rackaityte, Mustafa Özçam, Susan V. Lynch

**Affiliations:** Division of Gastroenterology Department of Medicine University of California, San Francisco, CA 94143, USA

## Abstract

Elevated infant fecal concentrations of the bacterial-derived lipid 12,13-dihydroxy-9Z-octadecenoic acid (12,13-diHOME) increase the risk for childhood atopy and asthma. However, the mechanisms by which this lipid contributes to disease development are largely unknown. We hypothesized that macrophages, which are key to both antimicrobial and antigen responses, are functionally and epigenetically modified by 12,13-diHOME leading to short- and long-term dysfunction with consequences for both antimicrobial and antigenic responses. Macrophages exposed to 12,13-diHOME are skewed toward inflammatory IL-1*β*^high^CD206^low^ cells, a phenomenon that is further amplified in the presence of common microbial-, aero-, and food-allergens. These IL-1*β*^high^CD206^low^ macrophages also exhibit reduced bacterial phagocytic capacity. In primary immune cell coculture assays involving peanut allergen stimulation, 12,13-diHOME promotes both IL-1*β* and IL-6 production, memory B cell expansion, and increased IgE production. Exposure to 12,13-diHOME also induces macrophage chromatin remodeling, specifically diminishing access to interferon-stimulated response elements resulting in reduced interferon-regulated gene expression upon bacterial lipopolysaccharide stimulation. Thus 12,13-diHOME reprograms macrophage effector function, B-cell interactions and promotes epigenetic modifications that exacerbate inflammatory response to allergens and mutes antimicrobial response along the interferon axis. These observations offer plausible mechanisms by which this lipid promotes early-life pathogenic microbiome development and innate immune dysfunction associated with childhood allergic sensitization.

## 1. Introduction

Delayed early-life gut microbiota diversification and infant gut metabolic dysfunction with the capacity to promote the canonical features of allergic inflammation *in vitro* are associated with heightened risk of atopy and asthma in later childhood [1, 2, 3, 4, 5]. This suggests that high-risk infant gut microbiota stimulate intestinal inflammatory conditions that prevent commensal microbial colonization and promote early-life pathogenic microbiome development and immune dysfunction. Supporting evidence for this model comes from primary human dendritic/T cell coculture experiments which demonstrated that cell-free fecal extracts of 1-month-old infants at high risk for asthma promoted expansion of CD4^+^IL4^+^ T-helper 2 (Th2) cells and increased interleukin 4 (IL-4) production while simultaneously reducing CD4^+^CD25^+^FoxP3^+^ regulatory T-cell frequency [4]. Metabolomic analysis of the fecal metabolome of these infants identified a lipid, 12,13-diHOME as a candidate molecule contributing to this immune dysfunction [4]. A terminal catalytic product of linoleic acid metabolism, 12,13-diHOME was detected in significantly higher concentrations in the feces of 1-month-old infants who subsequently developed childhood atopy or asthma [4], and its concentration was positively correlated with increasing risk of childhood disease [6]. Using the same primary coculture assays, exposure of DCs to 12,13-diHOME resulted in reduced frequency of T-regulatory (T-reg) cells and IL-10 production in a dose-dependent manner [6], indicating that increasing concentrations of this lipid to levels detected in high-risk infant stool, promotes allergic inflammation via its effect on regulatory T cell populations. An examination of the upstream effect of this lipid on antigen-presenting dendritic cells (DC) indicated that it altered expression of genes involved in lipid uptake, metabolism, and presentation and reduced IL-10 cytokine production [6]. Moreover, either peritoneal delivery of 12,13-diHOME or gut microbial production of this lipid promoted exacerbated airway allergic inflammation in cockroach-sensitized mice, characterized by increased airway expression of IL-1*β*, IL-1*α*, and tumor necrosis factor (TNF), decreased Treg frequency and increased serum immunoglobulin E (IgE) concentrations [6].

The pattern of airway gene expression observed in our murine experiments coupled with the observation that gut bacterial-produced 12,13-diHOME leads to elevated local concentrations of this lipid in the respiratory tract [6], led us to consider that the pro-allergic effect of 12,13-diHOME may expand beyond DCs to macrophages. These antigen presenting cells play a key role in innate immune responses to both allergens and microbes and have emerged as a cellular immune player in asthma [7]. Critical to both antigen presentation and pathogenic microbial clearance [8], macrophages undergo cellular reprograming in response to local environmental cues including pathogen exposure and local substrate availability which impacts their metabolism and effector function [9, 10, 11, 12]. Thus, we rationalized that elevated 12,13-diHOME concentrations in the infant gut microbiome may promote an inflammatory macrophage phenotype with reduced capacity for antimicrobial response, reprograming that may be instrumental in shaping both early-life antigenic and antimicrobial responses.

Here, we provide evidence that 12,13-diHOME promotes the expansion of inflammatory macrophages, particularly in the presence of common microbial and food antigenic stimuli. This oxylipin also reduces macrophage phagocytic capacity, induces secreted IL-1*β* and IL-6, promotes expansion of memory B-cells and increases production of IgE in antigen-stimulated coculture assays. Linking early-life microbial products with innate immune training, we show that 12,13-diHOME promotes epigenetic modification of the macrophage genome, specifically restructuring chromatin to render specific interferon stimulated response elements inaccessible. Thus, our study provides insights into how this early-life allergic asthma-predictive gut bacterial lipid influences macrophage function and epigenetics and promotes dysfunctional antigen-presenting cellular phenotypes associated with increased risk of allergic asthma development.

## 2. Materials and Methods

### 2.1. Cell Culture Studies

THP-1 human monocytes (ATCC® TIB-202™) were maintained in a humidified atmosphere at 37°C in RPMI-1640 medium (Gibco™, Thermo Fisher Scientific) supplemented with 10% heat-inactivated fetal bovine serum, 100 U ml^−1^ penicillin, 100 U ml^−1^ streptomycin, and 2 mM L-glutamine (Corning, Manassas, VA) and were activated into macrophages with 5 ng ml^−1^ Phorbol 12-Myristate 13-Acetate (PMA, Thermo Fisher Scientific) for 48 hr. Subsequently, cells in growth medium (nonpolarized controls) were treated with either IFN*γ* (20 ng ml^−1^, PeproTech, Rocky Hill, NJ) plus LPS (100 ng ml^−1^, Sigma–Aldrich) to induce IL-1*β*-expressing macrophages, or IL-4 (20 ng ml^−1^, R&D System, Minneapolis, MN) to induce CD206-expressing macrophages, or the experimental testing agents described, for 24 or 48 hr. Assay to confirm Caspase-1 activity by THP-1 macrophages was quantified by measuring enzymatic activity in cell culture supernatants using the Caspase-Glo® 1 Inflammasome Assay according to the manufacturer's instructions (Promega, Madison, WI), with luminescence read on a BioTek Cytation 3 multimode reader.

Human peripheral blood mononuclear cells (PBMCs) were purified from plasma obtained from healthy, deidentified donors (Blood Centers of the Pacific, San Francisco, CA) through the cell-sourcing program that ensures donor confidentiality. All donors sign an agreement acknowledging that their blood may be used for research. PBMCs were isolated using Ficoll–Hypaque gradient centrifugation. Primary monocytes were isolated from PBMC via CD14 MicroBeads (Miltenyi Biotec, Germany) enrichment. Cells were macrophage differentiated in RPMI-1640 medium supplemented with 10% heat-inactivated human AB positive serum (Sigma), 100 U ml^−1^ penicillin, 100 U ml^−1^ streptomycin, and 2 mM L-glutamine in a humidified atmosphere at 37°C for 6 days with two media changes yielding 96.9 ± 0.01% CD68 positive cells as determined by flow cytometry. To obtain IL-1*β*-polarized, macrophages were stimulated for 48 hr with 20 ng ml^−1^ IFN*γ* and 100 ng ml^−1^ LPS. To obtain CD206-polarized control cells, macrophages were stimulated with 20 ng ml^−1^ IL-4 plus 20 ng ml^−1^ IL-13 (Sigma–Aldrich). The oxylipin 12,13-diHOME (Cayman Chemical, Ann Arbor, MI) was solubilized in 0.1% DMSO and used to treat cells in nonlethal doses, ranging from 37.5 to 100 *µ*M. Antigens applied to cell treatment included, cockroach antigen (CRA) (20,000 PNU ml^−1^; Greer, Lenoir, NC) 5% (v/v); LPS 100 ng ml^−1^ (Sigma–Aldrich, L5543-2ML); albumin from chicken egg white (OVA) (Sigma, A5503-1G) 0.5 mg ml^−1^; and peanut extract (flour extract (Nuts.com) or protein antigens (f171, Greer)) 5% (v/v). Neither the Nuts.com nor the Sigma–Aldrich products are tested for endotoxin. Using Pierce™ Chromogenic Endotoxin Quant Kit (Thermo Scientific, detection range 0.1–1 EU ml^−1^), we quantified endotoxin in the peanut protein f171, peanut extract, and OVA. We did detect minimal endotoxin present in all three reagents: 0.125 EU ml^−1^ in peanut protein f171, 0.06 EU ml^−1^ in peanut extract, and 0.139 EU ml^−1^ in OVA, respectively. Peanut flour extract was obtained by mixing the peanut flour in 20% FBS-PBS-EDTA at 0.5 g ml^−1^, followed by bead beating in matrix D tubes on a FastPrep24 Homogenizer at 5.5 m s^−1^ for 45 s. Sterile liquid peanut extract was obtained by centrifugation of the peanut homogenate at 14,000 rpm for 5 min, followed by filtration of the resulting liquid through a 0.2 *μ*m filter.

For coculture assays, monocyte-differentiated macrophages at the end of 48 hr treatment were washed once in fresh media prior to coculture with lymphocytes. Autologous lymphocytes were enriched from the PBMCs by negative selection using the EasySep™ Direct Human Lymphocyte Isolation Kit (STEMCELL Technologies, Vancouver, Canada). These isolated cells were suspended in the TexMACS Medium (Miltenyi Biotec) prior to being added to macrophages at a ratio of 10 : 1 in the presence of soluble anti-CD28 (CD28.2, BD BioScience), anti-CD40 (5C3, Invitrogen) and anti-CD49d (9F10, BD BioScience) (1 *μ*g ml^−1^). Cells were cocultured for 10 days and replenished with fresh TexMACS Medium every 3 days. Cells were harvested for flow cytometry. Cell-free media from these cocultures were collected and evaluated by ELISA (ab195216, Abcam, detection range: 0.11–1.33 ng ml^−1^) for human IgE concentrations according to the manufacturers' instructions and for IgG using the Human Total IgG flex set (558679, BD Cytometric Bead Array, BD BioScience, detection range: 1.7–430 ng ml^−1^). To assess intracellular cytokine production, cell cultures were mixed with GolgiPlug (Gplug; BD Biosciences) for 6 hr before staining for flow cytometry. Each experiment was independently replicated three times. To quantify secreted cytokines in cell culture supernatants, human IL-1*β* and IL-6 cytokine flex sets (558279 and 558276, BD Cytometric Bead Array, BD BioScience) were used. Cytometric bead array target detection was captured using a LSRII cytometer followed by FCAP Array software (version 3) to calculate the concentration of assay targets.

### 2.2. Flow Cytometry

For flow cytometry, single-cell suspensions were stained. Stained cells were analyzed on a LSRII cytometer using the BD FACSDiva software (BD Biosciences). All cells were incubated with human BD Fc block (BD Biosciences) for 15 min prior to staining with flow antibodies. For macrophages, a panel of antibodies were used including anti-CD14 (63D3, 1 : 100; PerCp-Cy5.5, BioLegend), anti-CD16 (3G8, 1 : 50; BV-605, BioLegend), anti-CD68 (Y1/82A, 1 : 40; PE-Cy7, BioLegend), anti-CD206 (15–2, 1 : 50; APC-Cy7, BioLegend), and anti-IL-1*β* (H1b-98, 1 : 20; Pacific Blue, BioLegend). Dead cells were stained positive with LIVE–DEAD Aqua Dead Cell Stain (Life Technologies). We used permeabilization buffer (BD BioScience) to permeabilize cells before staining for the intracellular markers CD68 and IL-1*β*. For flow analysis, live THP-1 macrophages were gated as CD14^+^CD68^+^ cells (gating cascade as shown in *Supplementary figure 1*; cell viability, *Supplementary figure 1*). Live primary monocyte-derived macrophages were gated as CD16^+^CD68^+^ cells (cell viability in *Supplementary figure 1*, gating cascade, as shown in *Supplementary figure 1*). Among macrophage subpopulations, IL-1*β*-cells were IL-1*β*^Hi^, and CD206-cells were CD206^Hi^.

For the cocultured B cell subsets, cells were stained using panel of antibodies including BD Biosciences Abs anti-CD3 (SP34-2, 1 : 100, Alexa-700), anti-CD20 (2H7, 1 : 100, APC), anti-CD24 (ML5, 1 : 25, BV-650), anti-CD38 (HIT2, 1 : 50, PerCp-Cy5.5), anti-IgD (IA6-2, 1 : 100, PE), anti-CD19 (HIB19, 1 : 100, Affymatrix eBioscience), and anti-CD27 (M-T271, 1 : 20, Miltenyi Biotec). Upon flow analysis, live B cells were gated as CD3^−^CD19^+^ cells. Amongst the B cell subpopulations, naive were CD27^−^IgD^+^, memory cells were CD20^+^CD38^−^IgD^−^CD27^+^, and plasma cells were IgD^−^CD24^−^ both CD27^Hi^ and CD38^Hi^ (gating cascade as shown in *Supplementary figure 1*).

### 2.3. Phagocytosis Assay

We used PMA-differentiated THP-1 and Raw264.7 cells for phagocytosis assays. After 48 hr of treatment either with DMSO, 50 or 100 *µ*M of 12,13-diHOME, cells were washed with PBS after media removal, followed incubation with fluorescent pHrodo™ Green *Escherichia coli* BioParticles™ Conjugate (Thermo-Fisher) for 2 hr in serum-free RPMI-1640 medium at 37°C. The phagocytic events were detectable as shown in the fluorescent microscopic images (*Supplementary figure 1*). Cells were scraped from the plate and then washed three times with cold PBS to remove fluorescent conjugates that had not been internalized. Cells were next suspended with LIVE–DEAD Aqua Dead Cell Stain solution to stain at 4°C for 30 min. Finally, macrophages were fixed in 2% PFA, and analyzed by flow cytometry (*Supplementary figure 1*; cell viability is shown in *Supplementary figure 1*).

### 2.4. Gentamicin Protection Assay

RAW 264.7 cells were seeded in 96-well tissue culture plates. *E. coli* HB101 was cultured overnight in LB broth, washed with PBS, and resuspended to an OD_600_ of 0.7. The dose of Gentamicin to kill 100% *E. coli* was predetermined (*Supplementary figure 1*) before *E. coli* was incubated with an equal volume of *E. coli* BioParticles™ opsonizing reagent (Thermo-Fisher) at 37°C for 1 hr. Cells were washed with PBS, overlaid with the inoculum at a multiplicity of infection (MOI) of 10 and incubated for 2 hr at 37°C [13]. Supernatants were aspirated and cells were washed three times with PBS. Fresh RPMI-1640 supplemented with gentamicin (200 *µ*g ml^−1^) was added and cells were incubated at 37°C for 30 min. Cells were subsequently washed three times with PBS prior to lysis with Tween20 (1%, v/v in water). Lysates were then subjected to 10-fold serial dilutions and aliquots were plated on LB agar plates to determine the number of macrophage intracellular *E. coli*. The experiment was independently repeated twice with three technical replicates per treatment.

### 2.5. Animal Model

The mouse lung tissues that were subjected to bulk RNA-seq characterization were sections obtained from our previous study [6]. Briefly, 6-week-old female C57BL/6 mice were obtained from Jackson Laboratories and orally gavaged with the three *E. coli* strains (3EH) that overexpress epoxide hydrolase (EH) genes, NP_814872, YP_003971091, and YP_003971333, and linoleic acid (LA) prior to intratracheal CRA sensitization on days 1, 2, 3, 14 and a final challenge on day 21 (*Supplementary figure 1*). Each challenge was preceded by 4 days of oral gavage with either 75 *μ*l 3EH and LA or filter sterile 25% glycerol per mouse. Immediately before treatment, 250 *μ*l filter sterilized LA (Sigma–Aldrich) was added to each 500 *μ*l 3EH glycerol stock. On day 22, mice were euthanized and their lungs were collected and stored in RNA*later* (Invitrogen).

### 2.6. RNA Purification and qRT-PCR

Total RNA from human macrophages or mouse lungs was isolated with RNAqueous™-Micro Kit (Cat. No. AM1931, Thermo Fisher Scientific) and treated with DNase I. First-strand cDNA was synthesized using 200 ng RNA with high-capacity RNA-to-cDNA™ Kit (Cat. No. 4387406, Thermo Fisher Scientific) according to the manufacturer's instructions. Real-time PCR was performed in triplicate with SYBR Green master mix (Cat. No. 4368577, Life Technologies) in the QuantStudio™ 6 Flex Real-Time PCR System (Applied Biosystems, Foster City, CA). All primer pairs used for real-time PCR are listed in *Supplementary table 2*. The PCR reaction conditions were as follows: 50°C for 2 min, 95°C for 10 min (1 cycle), 95°C for 15 s, and 60°C for 1 min (40 cycles) and a final melting curve cycle of 95°C for 15 s, 60°C for 1 min, and 95°C for 15 s. The relative fold change in expression of all genes was normalized to *β*-actin expression by the 2^−*ΔΔ*CT^ (where CT is threshold cycle) method.

For the time-course study, human primary (CD14^+^) monocyte-differentiated macrophage cells were treated with either media, DMSO (0.1%, v/v), 50 *μ*M 12,13-diHOME alone or 50 *μ*M 12,13-diHOME 2 hr prior to the addition of peanut. Cells were harvested at 1, 2, 4, 8, 12, 24, and 48 hr posttreatment, washed twice in PBS, and RNA was extracted using the RNAqueous™ Micro Kit (Ambion). RNA was converted to cDNA using the protocol described above. A number of representative marker genes (primers listed in *Supplementary table 2*) were quantitated using the same PCR reaction conditions as described above. Data were normalized using *β*-actin expression and the relative fold change in expression was calculated using the 2^−*∆∆*CT^ method [14].

### 2.7. RNA-Sequencing and Analysis

From the time-course study, described above, RNA aliquots collected at 4, 8, and 24 hr were quantified and quality assessed using a Qubit2.0 (Thermo Fisher Scientific) and Agilent 2100 Bioanalyzer (Agilent), respectively. All RNA extracts had RNA Integrity Number (RIN) > 9. Indexed cDNA sequencing libraries were constructed using the SMART-Seq® v4 Ultra® Low Input RNA Kit (Takara Bio USA, Inc., Mountain View, CA) and a Nextera XT DNA Library Preparation Kit (Illumina, San Diego, CA), according to the manufacturer's instructions. The concentration and quality of the libraries were again measured by Qubit2.0 and Agilent 2100 Bioanalyzer, respectively, prior to sequencing. Twenty-four samples were randomly divided across two sequencing NextSeq runs. The mouse lung samples were sequenced on a single NextSeq 550Dx instrument (Illumina), using the NextSeq 500/550 High Output Kit v2.5 (300 Cycles) with paired-end configuration. Quality control was performed using FASTQC v0.11.3 and quality trimming and adapter filtering with Trimmomatic v0.33 [15]. The data in FASTQ format were mapped to human GRCh38 for human samples or mouse GRCm38 for mouse samples using the STAR v2.7.5a aligner (https://github.com/alexdobin/STAR). Gene list annotations were based on ensembl_hg_100 for human and ensembl_mg_104 for mouse.

For both human and mouse RNA-seq datasets, raw read counts were converted to CPM (counts per million) using the edgeR package in R (https://www.R-project.org). Lowly expressed genes were removed, retaining only genes with at least 1 CPM in at least three samples analyzed. Genes on the X, Y, and mitochondrial chromosomes were also removed. Raw counts were normalized using the trimmed mean of *M*-values normalization method [16] and mean–variance trend was adjusted using variance modeling in *Voom* [17].

For the human macrophage data, we conducted differential expression analysis for two comparisons: one comparing 12,13-diHOME to DMSO treatments, and the other comparing the combined treatment of 12,13-diHOME and peanut antigen to DMSO. For the mouse airway data, differential expression was performed for three comparisons: cockroach antigen and vehicle, *E. coli*-expressing 3-epoxide hydrolase and vehicle, and the combinatorial cockroach and 3-epoxide hydrolase and vehicle. Differential expression was performed using a linear model (limma) adjusting for both timepoint and individual effects. For both datasets, pathway analysis was performed using Gene Set Enrichment Analysis (GSEA), a threshold-free approach that overlays differential expressed genes onto a global molecular function and interaction network using R script generated by Alexander Pico, applying gene ontology, the Wiki Pathways database, and the Cytoscape visualization tool (https://bioconductor.org/packages/release/bioc/vignettes/rWikiPathways/inst/doc/Pathway-Analysis.html). Differentially expressed genes and enriched GSEA pathways were adjusted for multiple testing using the Benjamini–Hochberg method; significant genes and pathways were considered at FDR < 0.05.

### 2.8. ATAC-Sequencing and Analysis

We performed ATAC-seq, as previously described [18]. Briefly, 50,000 of either vehicle (DMSO) or 12,13-diHOME (50 *µ*M) treated human primary macrophages from four donors were lysed with cold lysis buffer (10 mM Tris-HCl, pH 7.4, 10 mM NaCl, 3 mM MgCl2 and 0.1% (v/v) Igepal CA-630). Immediately after lysis, nuclei were spun at 500 g for 10 min at 4°C. After pelleting the nuclei, the supernatant was removed and the nuclei resuspended in Tn5 transposase reaction mix (25 *µ*l 2x TD buffer, 2.5 *µ*l Tn5 transposase, and 22.5 *µ*l nuclease-free water) (Illumina Inc.). The transposition reaction was performed at 37°C for 30 min. Immediately after the transposition reaction, DNA was purified using ZYMO DNA Clean & Concentrator kit. Indexed libraries were generated using NEBNext® High-Fidelity DNA library prep PCR mix (New England Biolabs) and sequenced on an Illumina NovaSeq sequencer at QB3 Genomics (Berkeley, California).

After removing Nextera adaptors using Trimmomatic v0.36, 50 bp paired-end reads were aligned to the human genome (hg19) using Bowtie2 v2.3.4.3. After filtering reads from the mitochondrial genome and from unmapped contigs, paired reads with high mapping quality (MAPQ score >10) were retained using SAMTools v1.10. Duplicate reads were removed using the MarkDuplicates program, as implemented in Picard v2.18.29 (https://broadinstitute.github.io/picard/). ATAC-seq peak regions for each sample were called using MACS2 v2.1.0 with parameters–nomodel–shift -100–extsize 200. Blacklisted regions from ENCODE were also excluded from called peaks (http://hgdownload.cse.ucsc.edu/goldenPath/hg19/encodeDCC/wgEncodeMapability).

To generate a consensus set of unique peaks, bedtools v2.29.0 was used to pool peak calls across samples. Overlapping or duplicate peaks were consolidated into a single peak that ranged from the most 3′ base to the most 5′ base, resulting in 132,340 detected regions on chromosomes 1–22. Next, the number of reads aligned to each peak was determined for each sample using featureCounts in the subread package v1.5.3 and normalized to CPM peak-library mapped reads.

We next defined chromatin accessibility regions that were present in each individual as those regions at CPM > 1. Because of our small sample size, we used a combination of relaxed and stringent criteria to identify chromatin accessibility regions that were associated with either vehicle or 12,13-diHOME. Using relaxed criteria, we identified regions that were present in either all four vehicle-treated samples or all four 12,13-diHOME-treated samples. Next, using stricter criteria, we identified treatment-specific regions as those that were present in all four samples in one treatment group and were absent in all four samples from the other treatment group. Chromatin accessibility regions that were present in either all vehicle-treated samples or 12,13-diHOME-treated samples were referred to as lost and gained regions, respectively.

HOMER motif analysis (v4.10.0) was used to identify transcription factor binding motifs that were enriched within the less stringent numbers of lost and gained chromatin accessibility regions after 12,13-diHOME treatment, using all open areas of open chromatin as background and a Benjamini–Hochberg FDR-adjusted *p* < 0.05.

### 2.9. Statistical Analysis

All values are expressed as means ± SDs or means ± SEMs for each group. Statistical significance for normally distributed samples was assessed by using Kruskal–Wallis test; or by using nonparametric Mann–Whitney test with Prism software (GraphPad Software, La Jolla, Calif), and linear mixed model for repeated measures and/or account for biological replicates such as human donors were conducted in R v4.2.0 (Vigorous Calisthenics). Figures were prepared with either R v4.2.0 on RStudio (2022.02.1) or Prism. Differences with *p* values of less than 0.05 were considered statistically significant. All box plots indicate the interquartile range (IQR) and median.

## 3. Results

### 3.1. 12,13-diHOME Promotes Inflammatory Macrophages with Reduced Phagocytic Capacity

**Figure 1:**
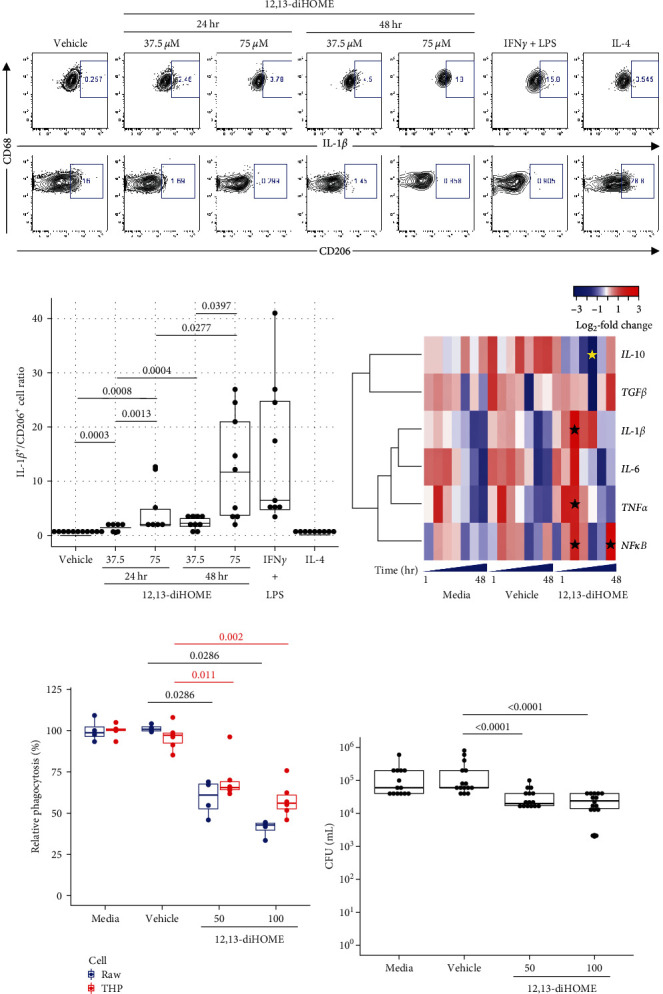
12,13-diHOME promotes inflammatory macrophage polarization and reduces phagocytic capacity, (a) Representative flow cytograms of IL-1*β* (CD68^+^ IL-1*β*^Hi^; upper panel) and CD206 (CD68^+^CD206^Hi^; lower panel) THP-1 macrophages following treatment with 37.5 or 75 *μ*M 12,13-diHOME for 24 or 48 hr (gating strategy provided in *Supplementary figure 1*). As controls, cells were stimulated 48 hr with 20 ng ml^−1^ IFN*γ* and 100 ng ml^−1^ LPS together to promote an IL-1*β* phenotype or 20 ng ml^−1^ IL-4 to induce a CD206 phenotype. (b) IL-1*β*^+^/CD206^+^ THP-1 macrophage ratio following treatment with 37.5 or 75 *μ*M 12,13-diHOME or vehicle (DMSO; control) for 24 or 48 hr (Brown–Forsythe and Welch ANOVA test). (c) Temporal gene expression (1, 2, 4, 8, 12, 24, and 48 hr) of macrophage cytokines associated with inflammatory functional states (*IL-1β*, *TNFα*, *IL-6*, *and NFκB* (*RELA*)) and anti-inflammatory functional states (*IL-10*, *TGFβ*) following exposure of primary human macrophages to 12,13-diHOME. Mean gene expression of triplicate reactions quantitated by qRT-PCR followed with 2^−*ΔΔ*CT^ calculation; each follow-up timepoint was compared to baseline. Significant (*p* < 0.05) findings are indicated with an asterisk (linear mixed model, *Supplementary table 2*). Dose-dependent reduction in phagocytosis of fluorescently labeled *E. coli* following exposure to 12,13-diHOME (50 or 100 *μ*M) exposure is observed in (d) Raw264.7 (blue) and THP-1 (red) macrophages and (e) confirmed using a gentamicin protection assay (Kruskal–Wallis test). All box plots indicate the interquartile range (IQR) and median.

To initially test the hypothesis that 12,13-diHOME affects macrophage function, human THP-1 macrophages were treated with nonlethal doses of this lipid, and CD14^+^CD68^+^ cells were assessed by flow cytometry following 24 or 48 hr of lipid exposure. While it is known that macrophage function exists within a network of functional states [19], our initial experiments simply assessed the canonical markers of inflammatory (IL-1*β*) or noninflammatory (CD206) macrophages by flow cytometry to broadly assess the effect of 12,13-diHOME on macrophage inflammatory status. Exposure to 12,13-diHOME increased the frequency of IL-1*β*-expressing macrophages with a concomitant decrease in CD206 expressing populations, a phenomenon that exhibited both time- and dose-dependency (Figures 1(a) and 1(b)). Caspase-1 cleaves and activates pro IL-1*β* [20]. To confirm that IL-1*β* expression represented active cytokine, we performed analysis of Caspase-1 expression in cell culture supernatants and demonstrated a dose-dependent increase in enzymatic activity with exposure to increasing 12,13-diHOME concentrations (*Supplementary figure 1*).

To further validate our observations, primary monocyte-derived macrophages isolated from the plasma of two healthy human donors were exposed to 12,13-diHOME and expression of key cytokines associated with inflammatory or anti-inflammatory functional states (*IL-6*, *IL-1β*, *TNFα*, *NFκB* (*RELA*), and *IL-10*, *TGFβ*) were assessed longitudinally over a 48-hr period. Compared with vehicle controls, 12,13-diHOME significantly increased transcription of *IL-1β*, *TNFα*, and *NFκ*B within 4 hr of exposure (*p* < 0.003 for all; linear mixed model; *Supplementary table 2*), with decreased *IL-10* and *TGFβ* expression evident by 12 hr postexposure (*p*=0.003 and *p*=0.117, respectively, Figure 1(c)). Thus, these data confirmed that exposure of macrophages to 12,13-diHOME can rapidly induce transcriptional reprograming associated with an inflammatory macrophage phenotype and that the use of IL-1*β* or CD206 markers in our flow cytometry assays represented a reasonable proxy for the inflammatory state of these macrophages.

Inflammatory macrophages commonly exhibit decreased phagocytic capacity compared to noninflammatory macrophages [21]. Given the capacity of 12,13-diHOME to promote inflammatory macrophage gene expression, we rationalized that this lipid also suppresses macrophage phagocytic function thus reducing microbial clearance capacity. To assess this, both human THP-1 and murine Raw264.7 macrophage cells were pretreated with 50 or 100 *µ*M of 12,13-diHOME for 48 hr. Cells were subsequently incubated with a pH-sensitive fluorescently labeled *E. coli* strain to assess phagocytosis by flow cytometry, as previously described [22]. Both THP-1 and Raw264.7 cells exhibited reduced phagocytosis following exposure to 12,13-diHOME in a dose-dependent manner (Figure 1(d)). To validate these observations, *E. coli* phagocytosis by 12,13-diHOME-treated macrophages was measured using a gentamicin protection assay [13]. Macrophages pretreated with 12,13-diHOME exhibited reduced numbers of intracellular *E. coli* (Figure 1(e)), indicating that this lipid reduces macrophage capacity for microbial phagocytosis, a key element of pathogen clearance.

### 3.2. 12,13-diHOME Exacerbates Macrophage Inflammatory Response to Antigenic Stimuli and Promotes Memory B Cell Expansion and Immunoglobulin E Production

**Figure 2:**
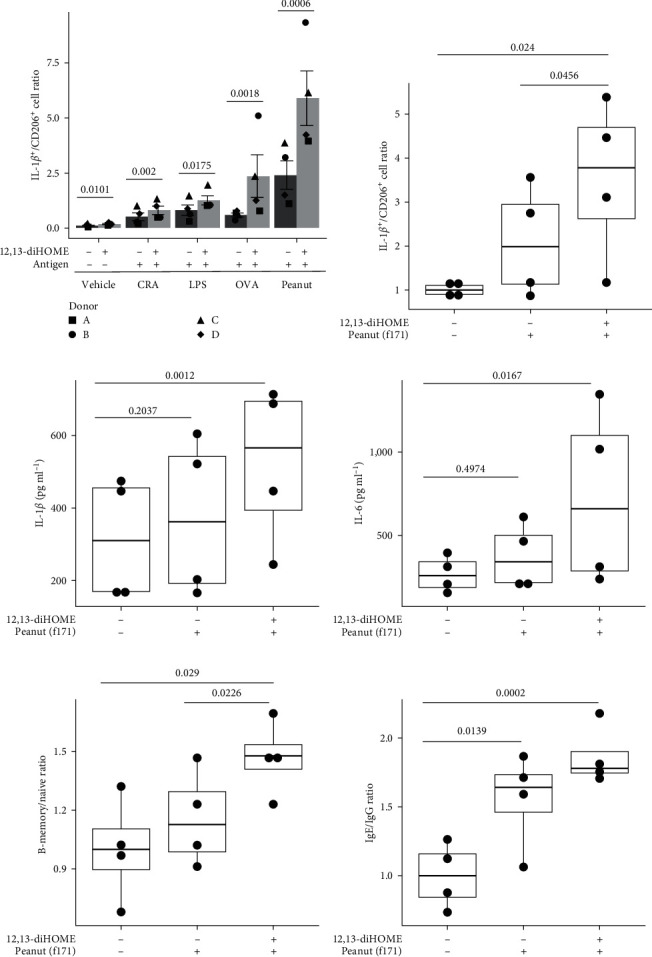
12,13-diHOME enhances inflammatory macrophages, IgE production, and cytokine secretion in the presence of antigenic stimulation. (a) 12,13-diHOME enhances allergen-induced a skew in IL-1*β*^+^/CD206^+^ cell ratio in primary human monocyte-derived macrophages from four adult donors (A, B, C, and D). Cells were treated with individual allergens, CRA, LPS, OVA, or peanut extract, in the absence or presence of 12,13-diHOME (37.5 *µ*M). Data represent the mean ± standard deviation. A linear mixed model was used to determine significant differences. In primary human macrophage/T/B cell (from two adult donors) coculture assays, macrophage stimulation with peanut allergen (f171) in the presence of 12,13-diHOME (50 *µ*M) increases (b) the elevated ratio of IL-1*β*^+^/CD206^+^ macrophages, secreted (c) IL-1*β*, and (d) IL-6 concentrations (pg ml^−1^) in the supernatant after 5 days of coculture (e) the ratio of memory-B to naïve-B cell populations, and (f) the ratio of secreted IgE to IgG (data listed in *Supplementary table 2*, and the bar plots of both absolute IgE and IgG concentrations listed as *Supplementary figure 1*) in the coculture supernatant (all box plots indicate the IQR and median; linear mixed model).

Given that elevated concentrations of fecal 12,13-diHOME are associated with allergic sensitization [6], we next tested whether exposure of macrophages to 12,13-diHOME in the context of allergen stimulation influenced their response to antigen. Using primary monocyte-derived macrophages from four adult donors and flow cytometry focused on IL-1*β* and CD206 markers, we demonstrated that common aero- and food-allergens (cockroach, lipopolysaccharide, OVA, and peanut) induced IL-1*β* expressing macrophages. Notably, antigen stimulation in the presence of 12,13-diHOME, significantly increased the frequency of IL-1*β*-expressing macrophages (Figure 2(a) and *Supplementary figure 1*). This was particularly evident in the context of peanut or OVA stimulation in the presence of 12,13-diHOME, which was associated with a more than twofold increase in the frequency of IL-1*β* expressing macrophages compared with antigen stimulation alone. These data suggest that 12,13-diHOME exacerbates inflammatory macrophage response to allergenic stimuli, particularly food antigens.

Macrophages play a key role in antigen-presentation [23] and oral tolerance [24], though little is known about their role in IgE-mediated food allergy [24]. Thus, using flow cytometry, we tested whether 12,13-diHOME enhanced the capacity of antigen-exposed macrophages to promote Immunoglobulin E (IgE) production by B cells in coculture assays that also included T cells (*Supplementary figure 1*). We focused on peanut antigen (f171) since our previous experiment indicated that 12,13-diHOME significantly exacerbated inflammatory macrophage expansion in the presence of peanut antigen stimulation (Figure 2(a)). Exposure of macrophages to peanut antigen (f171) in the presence of 12,13-diHOME increased the frequency of IL-1*β*-expressing cells (*p*=0.024, Figure 2(b)) and the concentrations of secreted IL-1*β* (*p*=0.0012, Figure 2(c)) and IL-6 (*p*=0.0167, Figure 2(d)) and also increased the ratio of memory to naïve B cells (*p*=0.029, Figure 2(e)). In addition, macrophages exposed to f171 in the presence of 12,13-diHOME increased B cell production of IgE at the expense of IgG (*p*=0.0002, Figure 2(f)). These findings were recapitulated when crude peanut flour was used as the antigenic stimulus (*Supplementary figure 1*).

### 3.3. 12,13-diHOME Rapidly Reprograms Macrophage Transcription

To more broadly understand how 12,13-diHOME promoted inflammatory macrophage expansion in the context of antigenic exposures, we performed bulk RNA-sequencing (RNA-seq) on monocyte-derived human macrophages from two donors exposed to either vehicle (DMSO), 12,13-diHOME or a combination of 12,13-diHOME and peanut flour (Pnut). Since our RT-PCR data indicated key cytokine gene expression changes between 4 and 24 hr, we focused RNA-seq analyses on 4, 8, and 24 hr postexposure. This permitted both repeated measures to increase power and identification of dynamic transcriptional responses that are associated with reprograming of macrophage phenotype. Analysis was performed by independently comparing treatment groups to the control (DMSO) after adjustment for both timepoint and donor effects. RNA-seq data, collected at 4-hr time point, identified gene expression changes in 270 genes following exposure to 12,13-diHOME (*Supplementary table 2*). These data indicate that 12,13-diHOME promoted upregulations of key genes associated with inflammatory macrophage activation including IL-1*α*, IL-1*β*, TNF, and CXCR5, all of which are recognized as pivotal players in the regulation of inflammatory responses (*Supplementary figure 1*) [25]. At 8-hr postexposure, a total of 20 (DMSO vs. 12,13-diHOME) and 1,308 (DMSO vs. 12,13-diHOME + Pnut) differentially expressed (DE) genes were identified, 12 of which were shared between treatments (FDR-adjusted *p* < 0.05, *Supplementary figure 1*; *Supplementary table 2*). Consistent with our initial qPCR-based observations indicating that 12,13-diHOME induced significant transcriptional changes 8 hr postexposure, hierarchical clustering of the top 100 DE genes (DMSO vs. 12,13-diHOME + Pnut) indicated that the most robust transcriptional changes occurred early following exposure of macrophages to 12,13-diHOME and peanut antigen with a peak transcriptional response at 8 hr (*Supplementary figure 1*). In addition, principal components analysis of all genes differentiated the three treatment groups (ANOVA, *p*=0.01 and 2.1 × 10^−4^ for PC1 and PC2, respectively; *Supplementary figure 1*), demonstrating that global transcription levels are altered by 12,13-diHOME alone and by the combination of 12,13-diHOME and peanut antigen.

**Figure 3:**
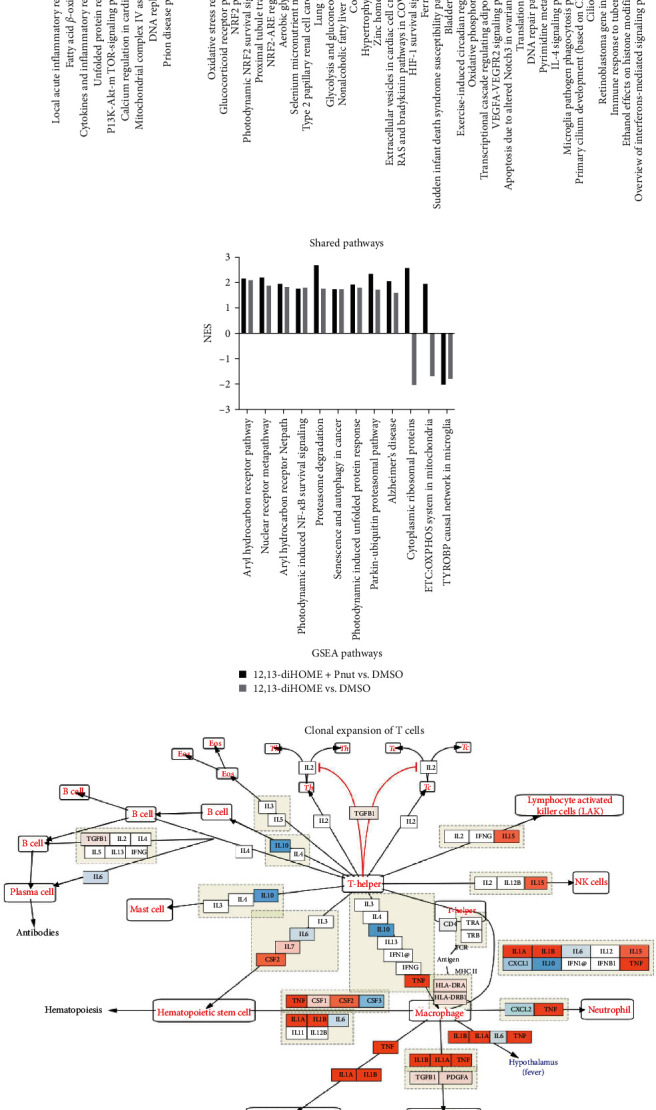
Pathway enrichment analysis of human macrophages 8-hr exposed to 12,13-diHOME and peanut (Pnut) allergen identifies pathways associated with inflammatory polarization. Pathways exclusively enriched in (a) 12,13-diHOME vs. DMSO (left) or 12,13-diHOME + Pnut vs. DMSO (right) treated macrophages. (b) Pathways enriched in both 12,13-diHOME vs. DMSO and 12,13-diHOME + Pnut vs. DMSO. NES, normalized enrichment score. Positive and negative NES indicate upregulated and downregulated pathways, respectively. (c) Cytokines and inflammatory response pathways induced by 12,13-diHOME. Genes in orange and blue show relatively higher and lower expression, respectively.

Gene set enrichment analysis (GSEA) identified transcriptional pathways associated with either exposure to 12,13-diHOME alone (Figure 3(a) left; *Supplementary table 2*, *n* = 20) or in combination with Pnut (Figure 3(a) right; *Supplementary table 2*, *n* = 50) compared with control (DMSO); 12 of these pathways were shared across both lipid-treated groups (Figure 3(b) and *Supplementary figure 1*). Pathways associated with 12,13-diHOME exposure included upregulation of local acute inflammatory response, PI3K-Akt-mTOR signaling, fatty acid beta-oxidation, and cytokine and inflammatory response pathways. The latter included upregulation of key pro-inflammatory genes related with inflammatory macrophages including *IL-1α*, *IL-1β*, and *TNFα*. Exposure to 12,13-diHOME in the presence of Pnut upregulated aerobic glycolysis and Parkin-ubiquitin proteasomal system pathways. The former represents an energy production pathway associated with inflammatory macrophage function [26], while the latter plays a role in innate defense against intracellular microbial pathogens [27, 28]. Consistent with transcriptional reprograming toward an inflammatory IL-1*β* macrophage state, exposure to 12,13-diHOME and Pnut downregulated IL-4 signaling, pathogen phagocytosis, and interferon-mediated signaling pathways (Figure 3(a), right panel). Transcriptional pathways shared by both lipid-exposed treatment groups (with or without Pnut exposure) included aryl hydrocarbon receptor, Parkin-ubiquitin proteasomal system and NF*κ*B survival signaling pathways (Figure 3(b)). The latter is related to LPS-induced TLR4 signaling and IL-1*β* production, a hallmark of classically activated inflammatory macrophages [29]. In contrast to the induced pro-inflammatory cytokines of the “Cytokines and inflammatory response pathway” (Figure 3(c), orange boxes), the pro-regulatory components of the pathway, such as *IL-10*, were suppressed by 12,13-diHOME and peanut treatment (Figure 3(c), blue boxes). Together these data confirmed that 12,13-diHOME exposure induces a pro-inflammatory transcriptional program in macrophages and more broadly appears to influence macrophage energy production and inflammatory cytokine gene expression in the context of peanut antigen stimulation.

### 3.4. Gut Bacterial-Derived 12,13-diHOME Induces Inflammatory Transcriptional Signatures in Mouse Airways

**Figure 4:**
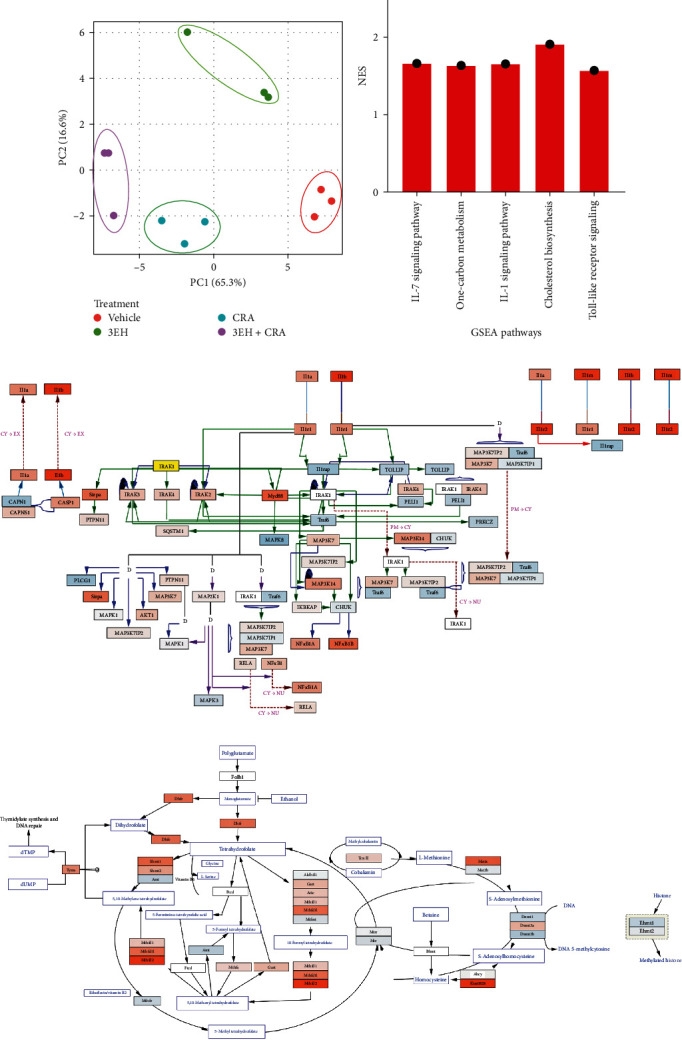
Gut microbial derived 12,13-diHOME exposure in context of airway allergen stimulation induces pathways related to inflammatory polarization and epigenetic modifications. (a) Principal components analysis of the normalized expression for all genes between mice treated with and without the combinatorial 3 epoxide hydrolase (3EH)-expressing *E. coli* and cockroach antigen (CRA) distinguishes the four treatment groups: no treatment (vehicle; red), 3EH alone (green), CRA alone (blue), and the combination of 3EH + CRA (purple). PC1 and PC2 were significantly associated with the four 3EH and CRA treatment groups (ANOVA, *p* = 4.9 × 10^−5^ and 5.8 × 10^−4^, respectively). (b) Five upregulated GSEA pathways after treatment with combinatorial 3EH + CRA compared with vehicle-group. (c) IL-1 signaling pathway. (d) One-carbon metabolism pathway. Genes in orange and blue show relatively higher and lower expression in mice treated with 3EH + CRA combinatorial group, respectively. NES, normalized enrichment score.

We previously reported that oral supplementation of mice with *Escherchia coli* engineered to produce 12,13-diHOME (3EH) increased serum and airway concentrations of this lipid and exacerbated airway allergic inflammatory response following sensitization with cockroach antigen (CRA; *Supplementary figure 1*) [6]. To determine whether the 12,13-diHOME mediated exacerbation of airway allergic response to CRA involved inflammatory pathways that were associated with macrophages expression signatures *in vivo*, airway transcriptomes from the four groups of animals in this experiment ((1) Control, (2) Cockroach-sensitized (CRA), or (3) 12,13-diHOME producing *E. coli* (3EH) supplementation alone, and (4) 12,13-diHOME producing *E. coli* (3EH) supplementation plus CRA (3EH + CRA); *n* = 3 per group) were assessed (*Supplementary table 2*). Differential expression analysis identified 480 and 614 DE genes for CRA or 3EH + CRA treated animals, respectively (compared with vehicle group; *Supplementary figure 1*); 397 overlapped. The top 100 DE genes for 3EH + CRA treated animals distinguished the four treatment groups (*Supplementary figure 1*). Consistent with this result, principal components analysis of all genes revealed distinct transcriptional responses across the four treatment groups (ANOVA, *p* = 4.9 × 10^−5^ and 5.8 × 10^−4^ for PC1 and PC2, respectively; Figure 4(a)). GSEA analysis identified 51 and 40 enriched pathways for CRA or 3EH + CRA treated animals, respectively (compared with vehicle group; *Supplementary table 2*); 35 of these pathways overlapped (*Supplementary figure 1*) indicating that, as observed in our *in vitro* peanut stimulation studies, allergen sensitization was the primary driver of airway transcriptional response in these animals. However, five pathways were uniquely enriched in the 3EH + CRA treated group (indicating induction by 3EH treatment), all of which were upregulated (Figure 4(b) and *Supplementary figure 1*). Three (IL-7 signaling, toll-like receptor, and IL-1 signaling pathways) are known to be involved in inflammatory macrophage states [30, 31, 32], the latter supporting our earlier findings of increased *IL-1β* expression in the presence of 12,13-diHOME (Figure 4(c)). A pathway involved in one-carbon metabolism, known to connect various metabolic pathways with epigenetic regulation including DNA methylation was also among the enriched pathways (Figure 4(d)) [33]. Notably, in our previous study, flow cytometry was employed on fresh murine lung tissue, revealing a nonsignificant yet evident trend toward increased alveolar macrophages in the airways of mice following treatment with either 3EH or 3EH + CRA (*Supplementary figure 1*). Importantly, the RNA utilized to generate the bulk-RNAseq dataset showcased in Figure 4 originated from these same well-preserved mouse lung tissues. These *in vivo* findings provide supplementary evidence that gut bacterial-produced 12,13-diHOME in combination with airway allergic antigen challenge promotes a distinct airway transcriptional program associated with inflammatory macrophage states and epigenetic modifications.

### 3.5. 12,13-diHOME Alters the Epigenetic Landscape of Macrophages

**Figure 5:**
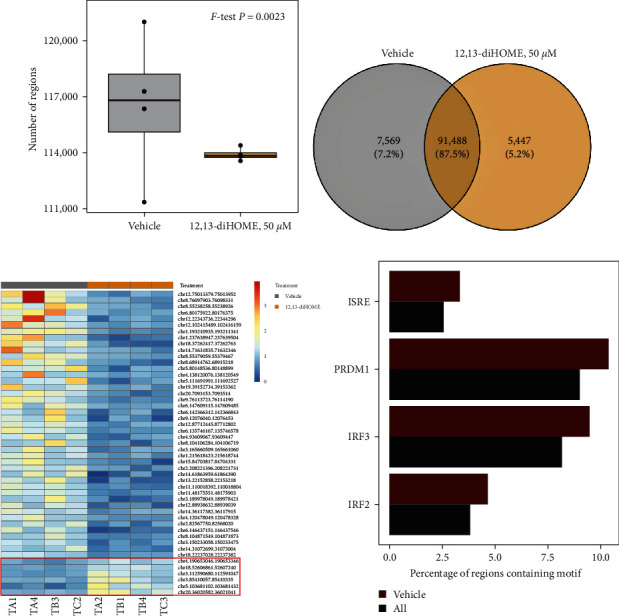
12,13-diHOME treatment reduced global chromatin accessibility specifically targeting interferon-response elements. (a) Compared to control (DMSO) exposure, human monocyte-derived macrophages exhibit a highly consistent number of chromatin accessibility regions impacted by exposure to 12,13-diHOME (50 *μ*M for 24 hr). Significance assessed using a two-sided *F*-test. (b) Numbers of chromatin accessibility regions that were present in all samples (CPM > 1) treated with either vehicle (DMSO; gray) or 12,13-diHOME (50 *μ*M for 24 hr) (orange). (c) Heatmap of the 49 regions present in 12,13-diHOME or vehicle-treated groups (detailed genomic annotations for all 49 regions are described in *Supplementary table 2*). Red box highlights the six regions that were present in all 12,13-diHOME-treated samples but absent in all vehicle-treated samples. (d) Four transcription factor binding sites (TFBSs) were exclusively identified 83 in the vehicle-treated samples indicating that they are inaccessible in the 12,13-diHOME treated samples. (magenta; vehicle; FDR-adjusted *p* < 0.05); all open areas of open chromatin were used as background (black; all).

Gene and epigenetic regulation represent key drivers of macrophage differentiation and polarization [34, 35]. We thus hypothesized that 12,13 di-HOME may promote epigenetic modifications to the macrophage genome that shape their function. We investigated global chromatin accessibility using ATAC-seq (assay for transposase-accessible chromatin with high-throughput sequencing) [18] on primary human macrophages treated with or without 12,13-diHOME. Compared to vehicle-treated cells that exhibited substantial variation in the number of chromatin accessibility regions, 12,13-diHOME treatment led to a relatively consistent number of accessible regions (Figure 5(a), *p*=0.0023, *F*-test), suggesting that this lipid exerts a highly specific effect on macrophage chromatin accessibility. We identified 7,569 (7.2% of total) chromatin accessibility regions uniquely present in vehicle-treated samples, whereas 5,447 (5.2% of total) regions were unique to 12,13-diHOME-treated samples (Figure 5(b)). Using stringent criteria (see Materials and Methods section), among the top 49 chromatin accessibility regions, six regions were present in all 12,13-diHOME-treated samples but absent in all vehicle-treated samples (highlighted in red box, Figure 5(c); *Supplementary table 2*), indicating that these regions became accessible following 12,13-diHOME treatment. The majority of the lost or gained chromatin accessibility regions were found in intronic and intergenic regions, indicating an important role for *cis*-regulatory elements in macrophage response to 12,13-diHOME (*Supplementary figure 1*).

Sequences within the vehicle-specific and the 12,13-diHOME-specific areas of chromatin were separately tested for enrichment of transcription factor binding sites (TFBSs) using HOMER motif analysis [36] to identify those TFBSs associated with altered chromatin accessibility in response to 12,13-diHOME. The 7,569 chromatin regions that were present only in the vehicle-treated samples were enriched for four TFBSs (Figure 5(d)); the top being interferon-stimulated response elements stimulated by type I interferons (IFNs). Consistent with this result, three other TFBSs enriched were positive regulatory domain zinc finger protein 1 (PRDM1) and IFN-regulatory factors IRF3 and IRF2; all of which regulate or are regulated by IFN and influence macrophage polarization phenotypes [37, 38]. These data further support our findings demonstrating downregulation of interferon-mediated signaling pathways in primary human macrophages exposed to both 12,13-diHOME and Pnut (Figure 3(a), the rightmost blue bar). No known TFBSs were enriched in chromatin regions that became accessible following 12,13-diHOME treatment. Overall, these data are consistent with 12,13-diHOME-mediated epigenetic reprograming of macrophages via altered global chromatin accessibility, including reduced access to binding sites of IFN-regulated transcription factors in regions of chromatin that shape macrophage effector phenotypes.

**Figure 6:**
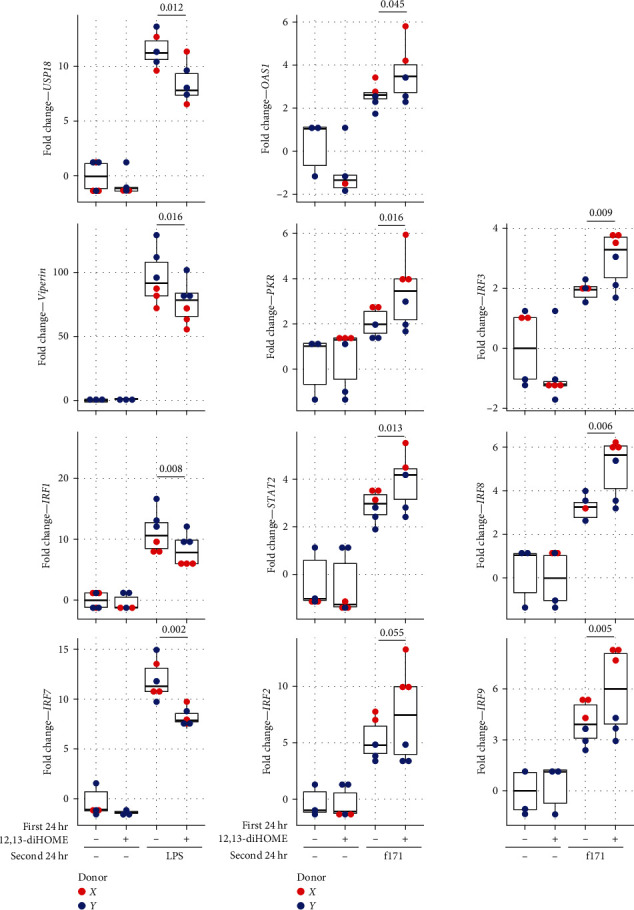
12,13-diHOME pretreatment influences IFN-regulated responses to either microbial (LPS) or peanut allergen (f171) stimulation. Pretreatment of primary human monocyte-derived macrophages from two donors (donor X, red dot; and Y, blue dot) with 12,13-diHOME (a) prior to LPS exposure significantly downregulates expression of IFN-regulated genes (*USP18* and *Viperin*) and factors (*IFR1* and *7*), (b) prior to peanut allergen (f171) exposure leads to upregulation of *OAS1*, *PKR*, *STAT2*, as well as *IRF 2*, *3*, *8*, and *9*. All box plots indicate the IQR and median.

IFN-mediated responses are critical to microbial clearance [39]. Thus, we rationalized that reduced access to IFN-regulated binding sites following 12,13-diHOME exposure may lead to muted IFN-mediated response to microbial or allergen exposures. We therefore tested whether 12,13-diHOME pretreatment of monocyte-derived primary human macrophages influenced subsequent IFN-regulated responses to microbial (LPS) stimulation, by assessing expression of a range of IFN-regulated genes (*STAT2*, *PKR*, *OAS1*, *USP18*, and *Viperin*) and interferon regulatory factors (*IRF1*, *2*, *3*, *5*, *6*, *7*, *8*, and *9*). Consistently, 12,13-diHOME pretreatment resulted in decreased expression of *USP18*, *Viperin*, *IRF 1*, and *7* in response to LPS stimulation (Figure 6(a)). To assess whether muted interferon responses in 12,13-diHOME-treated macrophages extended to nonmicrobial antigens, we repeated the experiment using peanut allergen (f171) stimulation. In contrast with LPS stimulation, peanut allergen stimulation of 12,13-diHOME pretreated macrophages resulted in upregulation of *STAT2*, *PKR*, *OAS1* as well as *IRF3*, *8*, and *9* (Figure 6(b)), indicating that bacterial and peanut antigens provoke distinct interferon-regulated macrophage responses that are significantly modified by 12,13-diHOME exposure.

## 4. Discussion

Delayed gut microbiome diversification and maturation [3, 40] as well as elevated fecal concentrations of 12,13-diHOME at 1 month of age are associated with an increased risk of allergic sensitization and asthma in childhood [4, 6]. That the infant gut microbiome and its metabolic products play an active role in the developmental origins of allergic disease is supported by the observation that cell-free products of high-risk infants promote canonical features of allergic inflammation in primary dendritic/T cell coculture assays [4, 6]. More specifically, exposure of DCs to elevated concentrations of 12,13-diHOME reduced their IL-10 production and the frequency of T-regulatory cells in coculture assays [4, 6], implicating this lipid as a driver of early-life immune dysfunction underlying allergic disease development.

Macrophages facilitate both antigen presentation and microbial clearance [8]. Thus, their functional state in early infancy likely plays a pivotal role in both allergic disease and microbiome development. It is well established that a Th2-inflammatory microenvironment characteristic of established allergic asthma promotes macrophage transition to an alternatively activated state that exacerbates allergic inflammation. However, tissue damage is known to promote the development of inflammatory macrophages [41, 42, 43]. Elevated fecal concentrations of 12,13-diHOME in infancy precede and predict allergic asthma development [4, 6]. Thus, we speculated that this lipid promotes an inflammatory macrophage phenotype with reduced bacterial clearance and antimicrobial response capacity that promotes the well-documented divergent early-life pathogenic microbiome development that precedes allergic asthma diagnosis [3, 4, 40]. Previous studies have demonstrated that macrophages are functionally polarized in response to microorganisms and host mediators [44], though the specific factors that facilitate polarization are less well understood. We showed that 12,13-diHOME elicits a rapid transcriptional response that includes upregulation of *IL-1β*, *TNFα*, *NFkB*, and *IL-6* whose expression is canonically associated with an inflammatory macrophage phenotype. These findings were corroborated by flow cytometry that indicated an expansion of IL-1*β* macrophages upon exposure to this lipid. Excessive or prolonged inflammatory macrophage polarization is known to promote tissue injury and pathogenesis [44], suggesting that early-life tissue destruction, promoted by the polarizing effect of 12,13-diHOME toward inflammatory macrophage function, may serve as a contributory factor to the developmental origins of allergy and asthma and a strong selective pressure on the microbial colonization landscape of the infant intestine. Notably, while 12,13-diHOME promoted expansion of inflammatory macrophages, antigen stimulation in the presence of this lipid further increased expansion of inflammatory macrophages. This suggests that as microbial, food, and aeroallergen antigenic loads increase in early life, elevated intestinal concentrations of 12,13-diHOME may further enhance inflammatory macrophage expansion and activity, accelerating tissue damage, and exerting a strong selective pressure on both the developing microbiome and immune memory.

To expand our understanding of how 12,13-diHOME promotes transcriptional programing of macrophages to facilitate phenotypic programing, we leveraged bulk RNA-sequencing. Consistent with an inflammatory macrophage phenotype, pathways upregulated following 12,13-diHOME exposure included inflammatory cytokine response, unfolded protein proteasomal system, fatty acid beta-oxidation, and PI3K-Akt-mTOR signaling. The PI3K-Akt-mTOR pathway mediates signals from multiple sources including insulin receptors, pathogen-associated molecular pattern receptors, cytokine receptors, adipokine receptors, and hormones. As a result, the PI3K pathway converges inflammatory and metabolic signals to regulate macrophage responses modulating their activation phenotype [45]. Arts et al. [46] previously demonstrated that glycolysis and glutamine metabolism, which are regulated by the Akt-mTOR pathway, are the primary mediators for induction of trained immunity by the tuberculosis vaccine Bacillus Calmette–Guerin in monocytes. Both P13K-Akt-mTOR signaling pathway as well as glycolysis and gluconeogenesis pathways were upregulated by 12,13-diHOME, implicating a plausible route by which this lipid may facilitate epigenetic modifications.

Though B-cell mediated immunoglobulin production is pivotal to allergy [47] and macrophage/B cell interactions are well described in the oncology field [48], little is known of interactions between macrophages and B cells in the origins or pathogenesis of allergic disease. Our observation that 12,13-diHOME exacerbated inflammatory macrophage expansion in the presence of a range of common food and aeroallergens prompted us to examine the downstream effect of 12,13-diHOME on B-cell activity using macrophages as the exposed antigen presenting cell. Using coculture assays, we provide evidence that 12,13-diHOME promotes expansion of memory B cells and an increased ratio of IgE to IgG in the presence of peanut antigen. Specific to food antigens, IgG can confer protection from food allergy either by blocking IgE binding or interfering with IgE function [49]. IgG isotypes are also known to block hypersensitivity reactions by competing with IgE and can inhibit IgE-mediated activation of effector cells by binding to the inhibitory receptor Fc*γ*RII-B [50]. Thus, our findings indicate that exposure of peanut-stimulated macrophages to elevated concentrations of 12,13-diHOME promotes immunoglobulin class switching toward IgE production and humoral immunity. These B cell features are known to promote allergy and facilitate rapid response to subsequent antigen re-exposure [51].

Epigenetic modifications are increasingly recognized as a key mechanism by which microbes influence host cell phenotypes, promoting longer term phenotypic programing of mammalian cells [52]. For example, recent studies have demonstrated that microbial-derived metabolites such as the short-chain fatty acids butyrate and acetate, inhibit histone deacetylases [53]. Gut microbiota may also regulate the concentration and/or activity of endogenously produced host metabolites that in turn may affect the host epigenome [52]. Indeed, Krautkramer et al. [54] demonstrated that microbial colonization regulates global histone acetylation and methylation in multiple host tissues in a diet-dependent manner. Our findings indicate that 12,13-diHOME alters the epigenetic landscape of macrophages via chromatin remodeling. Notably, macrophage genomic loci rendered inaccessible following 12,13-diHOME exposure were relatively specific and enriched for key transcription factors that regulate or are regulated by IFN, including ISRE, IRF3, and IRF2. Interferon regulatory factors (IRFs) further promote macrophage polarization [55] and are involved in apoptosis [56]. As importantly, these transcriptional factor binding sites also regulate antiviral and bacterial pathogen responses [57]. Notably, IRF2 is required for induction of TLR3 and other interferon-inducible genes critical to mount antiviral responses [58] and severe viral respiratory infection in early life is a risk factor for asthma development in later childhood [59]. Thus, our data suggest that gut bacterial derived 12,13-diHOME may mitigate interferon-regulated antimicrobial responses to intracellular pathogens, thus increasing susceptibility and severity of early-life viral infection and subsequent asthma risk in childhood. Our findings indicate that macrophages pretreated with 12,13-diHOME exhibit altered interferon-mediated responses upon subsequent microbial exposure. More specifically, pretreated macrophages exposed to LPS exhibited reduced expression of *IRF1* and *7*, as well as *Viperin* and *USP18* (negatively regulates inflammatory responses triggered by type I IFNs). IRF1 regulates Viperin which promotes TLR7 and TLR9-dependent production of IFN*β* [60], while IRF7 activates IFN*α* and *β*-mediated signaling. Thus, our data indicate that 12,13-diHOME reduces innate immune capacity to mount interferon-mediated responses to microbial stimulation both at the epigenetic and transcriptional levels. Indeed, an emerging concept in the field of childhood atopy and asthma implicates failed control of microbial pathogens in the developmental origins of the disease [61]. Our observations that 12,13-diHOME promotes inflammatory macrophages with reduced antimicrobial phagocytic and IFN response capacity further supports this model.

Taken together, our findings provide evidence that elevated concentrations of 12,13-diHOME increase the frequency of inflammatory macrophages, a phenotype that is exacerbated in the presence of antigenic stimuli. Our data also indicate that in addition to broadly reprograming macrophage transcription to support inflammatory macrophage polarization, 12,13-diHOME promotes epigenetic modifications that specifically render IFN-response elements inaccessible and downregulate interferon-regulated responses critical to control of microbial pathogens. These data coupled with evidence for 12,13-diHOME induced impairment of bacterial clearance, expansion of memory B-cell populations, and increased production of IgE offer evidence that this lipid promotes immune dysfunctional features pivotal to the origins or allergy and asthma. These observations suggest a model in which pathogenic infant gut microbiomes and more specifically bacterial production of 12,13-diHOME promotes an inflammatory environment with reduced capacity for pathogen clearance, thus permitting pathogenic microbiome development and promoting maladaptive immunity during a critical window of early-life immune maturation.

## Data Availability

The human RNA-seq, the mouse RNA-seq, and the human ATAC-seq datasets supporting the findings in this article are available in the Gene Expression Omnibus (GEO) repositories under the accession series GSE207529 (https://www.ncbi.nlm.nih.gov/geo/query/acc.cgi?acc=GSE207529). Additional code information used to analyze the data reported in this paper is available at the GitHub (https://github.com/dinlind1/12-13-diHOME-Macrophages). Requests for the *E. coli* strains used in this study and further information should be directed to and will be fulfilled by the corresponding author, Dr. Susan V. Lynch (http://susan.lynch@ucsf.edu).

## Supplementary Materials

Supplementary 1 Figure 1: THP-1 macrophages flow gating, macrophage phagocytosis, cell viability, and Gentamicin dose predetermination. Figure 2: antigens on THP-1 macrophage polarity, cell viability, and flow gating cascade of human primary macrophages. Figure 3: macrophage/T/B cell assays; peanut protein (f171) dose titration; and, flow gating cascade of human primary B cells. Figure 4: transcriptional landscape of human macrophages following treatment with 12,13-diHOME and peanut antigen. Figure 5: altered transcriptional landscape in an airway challenge mouse model. Figure 6: functional annotations of the lost and gained chromatin accessibility regions following 12,13-diHOME treatment.

Supplementary 2 Table 1: list of qRT-PCR primers. Table 2: linear mixed model analyses of time-course qRT-PCR. Table 3: differentially expressed genes in primary human macrophages after 4 hr treatment with 12,13-diHOME. Table 4: differentially expressed genes in primary human macrophages after 8 hr treatment with 12,13-diHOME. Table 5: differentially expressed genes in primary human macrophages after 8 hr treatment with combinatorial 12,13-diHOME and peanut. Table 6: enriched gene set enrichment analysis (GSEA) pathways in primary human macrophages after treatment with 12,13-diHOME. Table 7: enriched gene set enrichment analysis (GSEA) pathways in primary human macrophages after treatment with combinatorial 12,13-diHOME and peanut. Table 8: differentially expressed genes in murine airway cells after treatment with *E. coli*-expressing 3-epoxide hydrolase. Table 9: differentially expressed genes in murine airway cells after treatment with cockroach antigen. Table 10: differentially expressed genes in murine airway cells after treatment with combinatorial cockroach antigen and *E. coli*-expressing 3-epoxide hydrolase. Table 11: enriched gene set enrichment analysis (GSEA) pathways in murine airway cells after treatment with cockroach antigen. Table 12: enriched gene set enrichment analysis (GSEA) pathways in murine airway cells after treatment with combinatorial cockroach antigen and *E. coli*-expressing 3-epoxide hydrolase. Table 13: detailed ATAC-seq genomic annotations for 49 differentially accessible chromatin regions. Table 14: human IgE ELISA and IgG CBA data from the *ex vivo* coculture assay.
