# Supplementary material for: 12,13-diHOME Promotes Inflammatory Macrophages and Epigenetically Modifies Their Capacity to Respond to Microbes and Allergens
Source: J Immunol Res. 2024 Jun 29;2024:2506586. doi: 10.1155/2024/2506586 (PMC11227377; doi:10.1155/2024/2506586)

Table.S1

Huamn Gene	Forward	Reverse
β-Actin	AAGATGACCCAGATCATGTTTGAGACC	AGCCAGTCCAGACGCAGGAT
IL-10	AACAAGAGCAAGGCCGTGG	GAAGATGTCAAACTCACTCATGGC
IL-1b	AAACAGATGAAGTGCTCCTTCCAGG	TGGAGAACACCACTTGTTGCTCCA
IL-6	GATGGCTGAAAAAGATGGATGC	CTGCAGGAACTGGATCAGGACT
NFkB (RELA)	TCTGCTTCCAGGTGACAGTG	ATCTTGAGCTCGGCAGTGTT
TGFb	GCGTGCTAATGGTGGAAAC	CGGTGACATCAAAAGATAACCAC
TNFa	AGGCGGTGCTTGTTCCTCAG	GGCTACAGGCTTGTCACTCG
luciferase	GCAACACCCCAACATCTTC	CCACATAGTCCACGATCTCC
OAS1	TTGACTGGCGGCTATAAACC	TGGGCTGTGTTGAAATGTGT
PKR	GGAAAGCGAACAAGGAGTAAGG	CCAAAGCGTAGAGGTCCACT
STAT2	TATCACAGCCAGTGCCAGAG	CTGATTCCCATCCTTGGAGA
USP18	AACGTGCCCTTGTTTGTCC	GCAGTCTCTCCACCAAGTGC
Viperin	CCAACCAGCGTCAACTATCACTT	GGAAACAGAAGCCGCATTTG
IRF1	CCAAGAGGAAGTCATGTG	TAGCCTGGAACTGTGTAG
IRF2	AAAACATGGAAGGCGAATTTCAG	CCAGAGATGACTCAACTGGTTC
IRF3	TCGTGATGGTCAAGGTTGT	AGGTCCACAGTATTCTCCAG
IRF5	ATGCTGCCTCTGACCGA	GCCGAAGAGTTCCACCTG
IRF6	CTCATCTTGGTTCAGGTCATTC	CGGACACTGCCACTATCA
IRF7	CAGATCCAGTCCCAACCAAG	GTCTCTACTGCCCACCCGTA
IRF8	AATGCAAACTAGGCGTGGCA	TAATCGTCCACAGAAGGCTCC
IRF9	GTGCTGGGATGATACAGCTAAG	CAGGCGAGTCTTCCAGACAG

Table.S2

Linear mixed model fit by REML. t-tests use Satterthwaite's method ['lmerModLmerTest']

	Fixed effects:	Estimate	Std. Error	df	t value	Pr(>|t|)	
IL-1b	(Intercept)	5.077	4.267	8.867	1.19	0.26503	
	DiHOME1213	-0.147	6.462	27	-0.023	0.98202	
	as.factor(Hour)2	1.91	5.276	27	0.362	0.72018	
	as.factor(Hour)4	1.02	5.276	27	0.193	0.84809	
	as.factor(Hour)8	-2.677	5.276	27	-0.507	0.61608	
	as.factor(Hour)12	-5.442	5.276	27	-1.031	0.31155	
	as.factor(Hour)24	-14.9	5.276	27	-2.824	0.0088	**
	as.factor(Hour)48	-11.764	5.276	27	-2.229	0.0343	*
	DiHOME1213:as.factor(Hour)2	4.435	9.139	27	0.485	0.63141	
	DiHOME1213:as.factor(Hour)4	28.696	9.139	27	3.14	0.00407	**
	DiHOME1213:as.factor(Hour)8	12.354	9.139	27	1.352	0.18767	
	DiHOME1213:as.factor(Hour)12	17.943	9.139	27	1.963	0.05998	.
	DiHOME1213:as.factor(Hour)24	10.008	9.139	27	1.095	0.28314	
	DiHOME1213:as.factor(Hour)48	6.938	9.139	27	0.759	0.45434	
TNFa	Fixed effects:	Estimate	Std. Error	df	t value	Pr(>|t|)	
	(Intercept)	2.6287	0.5979	5.4931	4.397	0.00565	**
	DiHOME1213	1.34843	0.83531	27	1.614	0.11809	
	as.factor(Hour)2	6.51334	0.68202	27	9.55	3.78E-10	***
	as.factor(Hour)4	1.50328	0.68202	27	2.204	0.03622	*
	as.factor(Hour)8	-0.72806	0.68202	27	-1.067	0.2952	
	as.factor(Hour)12	-2.01133	0.68202	27	-2.949	0.00651	**
	as.factor(Hour)24	-4.80694	0.68202	27	-7.048	1.41E-07	***
	as.factor(Hour)48	-5.53451	0.68202	27	-8.115	1.02E-08	***
	DiHOME1213:as.factor(Hour)2	-0.80935	1.1813	27	-0.685	0.4991	
	DiHOME1213:as.factor(Hour)4	3.86867	1.1813	27	3.275	0.0029	**
	DiHOME1213:as.factor(Hour)8	1.41564	1.1813	27	1.198	0.24119	
	DiHOME1213:as.factor(Hour)12	0.25542	1.1813	27	0.216	0.83044	
	DiHOME1213:as.factor(Hour)24	0.74757	1.1813	27	0.633	0.53217	
	DiHOME1213:as.factor(Hour)48	-0.05368	1.1813	27	-0.045	0.96409	
IL-6	Fixed effects:	Estimate	Std. Error	df	t value	Pr(>|t|)	
	(Intercept)	6.7448	1.8041	2.7894	3.739	0.04136	*
	DiHOME1213	0.3198	2.1263	27	0.15	0.88212	
	as.factor(Hour)2	-0.275	1.7361	27	-0.158	0.87591	
	as.factor(Hour)4	0.7255	1.7361	27	0.418	0.68096	
	as.factor(Hour)8	-3.1502	1.8812	27	-1.675	0.11128	
	as.factor(Hour)12	-6.0536	1.8812	27	-3.218	0.00476	**
	as.factor(Hour)24	-12.4948	1.8812	27	-6.642	3.08E-06	***
	as.factor(Hour)48	-13.66	2.1739	27	-6.284	6.03E-06	***
	DiHOME1213:as.factor(Hour)2	-3.4948	3.007	27	-1.162	0.26033	
	DiHOME1213:as.factor(Hour)4	-2.6724	3.5015	27	-0.763	0.45519	
	DiHOME1213:as.factor(Hour)8	-3.9063	3.0931	27	-1.263	0.22272	
	DiHOME1213:as.factor(Hour)12	-1.3464	3.5566	27	-0.379	0.70942	
	DiHOME1213:as.factor(Hour)24	-2.5745	3.5566	27	-0.724	0.47843	
	DiHOME1213:as.factor(Hour)48	3.7465	3.7923	27	0.988	0.33611	
NFkB	Fixed effects:	Estimate	Std. Error	df	t value	Pr(>|t|)	
	(Intercept)	1.7687	0.2502	2.61236	7.069	0.00904	**
	DiHOME1213	-0.33835	0.28297	27	-1.196	0.24222	
	as.factor(Hour)2	0.77487	0.23105	27	3.354	0.00237	**
	as.factor(Hour)4	0.62553	0.23105	27	2.707	0.01162	*
	as.factor(Hour)8	0.58506	0.23105	27	2.532	0.01746	*
	as.factor(Hour)12	-0.34854	0.23105	27	-1.509	0.14304	
	as.factor(Hour)24	0.01761	0.23105	27	0.076	0.93982	
	as.factor(Hour)48	0.33705	0.23105	27	1.459	0.15616	
	DiHOME1213:as.factor(Hour)2	0.31015	0.40018	27	0.775	0.44507	
	DiHOME1213:as.factor(Hour)4	1.2374	0.40018	27	3.092	0.00458	**
	DiHOME1213:as.factor(Hour)8	0.57326	0.40018	27	1.432	0.16348	
	DiHOME1213:as.factor(Hour)12	0.16957	0.40018	27	0.424	0.67511	
	DiHOME1213:as.factor(Hour)24	0.19198	0.40018	27	0.48	0.63528	
	DiHOME1213:as.factor(Hour)48	2.15126	0.40018	27	5.376	1.11E-05	***
IL-10	Fixed effects:	Estimate	Std. Error	df	t value	Pr(>|t|)	
	(Intercept)	0.3543	0.952	7.997	0.372	0.7194	
	DiHOME1213	-0.2626	1.4197	27	-0.185	0.85465	
	as.factor(Hour)2	-1.3113	1.1592	27	-1.131	0.26793	
	as.factor(Hour)4	-1.3508	1.1592	27	-1.165	0.25408	
	as.factor(Hour)8	-0.3891	1.1592	27	-0.336	0.7397	
	as.factor(Hour)12	-0.3584	1.1592	27	-0.309	0.75955	
	as.factor(Hour)24	-0.402	1.1592	27	-0.347	0.73147	
	as.factor(Hour)48	0.3186	1.1592	27	0.275	0.7855	
	DiHOME1213:as.factor(Hour)2	-0.8945	2.0078	27	-0.446	0.6595	
	DiHOME1213:as.factor(Hour)4	-0.4338	2.0078	27	-0.216	0.83057	
	DiHOME1213:as.factor(Hour)8	-3.1246	2.0078	27	-1.556	0.13129	
	DiHOME1213:as.factor(Hour)12	-6.5614	2.0078	27	-3.268	0.00295	**
	DiHOME1213:as.factor(Hour)24	-1.4838	2.0078	27	-0.739	0.46627	
	DiHOME1213:as.factor(Hour)48	-0.3206	2.0078	27	-0.16	0.87431	
TGFb	Fixed effects:	Estimate	Std. Error	df	t value	Pr(>|t|)	
	(Intercept)	0.7151	1.1465	1.1685	0.624	0.63233	
	DiHOME1213	-0.5608	0.5718	27	-0.981	0.33542	
	as.factor(Hour)2	-0.3959	0.4669	27	-0.848	0.40388	
	as.factor(Hour)4	-0.5825	0.4669	27	-1.248	0.22287	
	as.factor(Hour)8	-0.7124	0.4669	27	-1.526	0.13867	
	as.factor(Hour)12	-2.8552	0.4669	27	-6.115	1.56E-06	***
	as.factor(Hour)24	-0.9142	0.4669	27	-1.958	0.06064	.
	as.factor(Hour)48	-1.5091	0.4669	27	-3.232	0.00323	**
	DiHOME1213:as.factor(Hour)2	0.485	0.8087	27	0.6	0.55367	
	DiHOME1213:as.factor(Hour)4	0.4328	0.8087	27	0.535	0.5969	
	DiHOME1213:as.factor(Hour)8	0.5182	0.8087	27	0.641	0.52707	
	DiHOME1213:as.factor(Hour)12	-1.3075	0.8087	27	-1.617	0.11756	
	DiHOME1213:as.factor(Hour)24	0.5607	0.8087	27	0.693	0.49401	
	DiHOME1213:as.factor(Hour)48	2.6308	0.8087	27	3.253	0.00306	**

Table.S3
Genes by 12,13-diHOME alone compared to DMSO at 4-hour

Gene	log2FC	Unadjusted P	Adjusted P
EREG	2.89153076	3.76748E-05	0.309497055
MT1X	3.96541951	7.70302E-05	0.309497055
GPR68	1.93834681	8.31748E-05	0.309497055
MT1F	2.7997946	0.000122556	0.309497055
ABTB2	3.08698529	0.000138252	0.309497055
RARG	2.96064395	0.0001499	0.309497055
MT1A	6.58806057	0.000158402	0.309497055
MT1L	4.77618601	0.000189306	0.309497055
ZFP36L2	-1.8958747	0.000226131	0.309497055
AL135818.1	2.02760092	0.000240353	0.309497055
RAB38	2.34807216	0.00025144	0.309497055
MT1M	4.82114063	0.000265872	0.309497055
MT2A	2.76215389	0.0003338	0.358680841
GRHL1	2.87922588	0.000390418	0.389553934
CDCA7L	1.74849899	0.000621399	0.575801529
THBD	2.13654981	0.000659519	0.575801529
MT1E	4.03235514	0.000737631	0.606115702
CYP1A1	5.27550784	0.000885452	0.68716027
AC010883.3	-1.7414144	0.001231795	0.905628708
TNF	1.28528347	0.001430735	0.975288893
C1orf115	3.05586658	0.00146618	0.975288893
MT1G	4.22841367	0.001613527	0.99992578
DNAH11	1.80131871	0.001702565	0.99992578
CNKSR3	2.93736528	0.001759462	0.99992578
CYP1B1	1.11656816	0.001980489	0.99992578
INPP5K	1.10688799	0.002251952	0.99992578
IL1A	1.66206614	0.002444569	0.99992578
CYP1B1-AS1	1.20047795	0.002608456	0.99992578
MACIR	1.5857079	0.002618666	0.99992578
SHF	3.10893586	0.002823909	0.99992578
MT1DP	5.08996302	0.002894769	0.99992578
CXCR5	1.58356551	0.003340996	0.99992578
HINFP	1.34244609	0.003411746	0.99992578
FCAR	1.3983728	0.003432148	0.99992578
BCL11A	-1.7746373	0.003676547	0.99992578
SAMD10	2.10771964	0.00375798	0.99992578
PRMT9	1.10373469	0.003815187	0.99992578
PLA2G4A	1.48087197	0.004157452	0.99992578
RAD51	1.66674774	0.004384366	0.99992578
GAB1	2.27057954	0.004390867	0.99992578
MT1H	3.88960356	0.004438675	0.99992578
ERCC6	1.27104468	0.00455252	0.99992578
FBXL20	1.14191727	0.004559565	0.99992578
LRG1	2.1708142	0.00457143	0.99992578
ORC6	1.70286524	0.004960409	0.99992578
ZBTB2	1.91300849	0.004986882	0.99992578
PRRG4	1.62303182	0.005046432	0.99992578
LHFPL6	2.03922049	0.0053223	0.99992578
RHOBTB1	1.84272252	0.005369886	0.99992578
CAVIN1	2.13277247	0.005401245	0.99992578
AC026333.3	1.80020802	0.005528373	0.99992578
CRYBG3	1.04587377	0.005660082	0.99992578
BCL9L	1.55355118	0.005724255	0.99992578
PAQR8	-1.3325102	0.005757507	0.99992578
AC005841.1	1.76342713	0.005916919	0.99992578
SERPINB2	2.60092978	0.005958007	0.99992578
PIF1	2.76969407	0.005980343	0.99992578
FKBP4	1.70650772	0.006013949	0.99992578
ASB13	1.1178597	0.006160468	0.99992578
ARHGAP20	3.47263551	0.0065125	0.99992578
POLH	1.13230151	0.006896168	0.99992578
SSBP2	1.13138458	0.007181712	0.99992578
AC110491.2	1.41126263	0.007202355	0.99992578
CLCF1	1.11468003	0.007268893	0.99992578
ZNF295-AS1	3.19328586	0.007489776	0.99992578
RAB29	1.07048576	0.007839814	0.99992578
LPAR2	1.03673487	0.007915528	0.99992578
GPR65	-1.2265423	0.007987153	0.99992578
AKR7A3	2.03934606	0.007987809	0.99992578
KLHL11	1.43396951	0.008420617	0.99992578
CREB5	1.64595198	0.008528986	0.99992578
SS18	0.89989585	0.008578047	0.99992578
PTGS2	2.07757749	0.008582832	0.99992578
ASB2	1.67564523	0.008619207	0.99992578
CDC42EP4	1.55950449	0.00940216	0.99992578
TPD52	1.13246012	0.010376778	0.99992578
BTG2	0.96640412	0.010507532	0.99992578
SLC38A1	1.57579156	0.01057408	0.99992578
NREP	1.31594455	0.010579658	0.99992578
SLC19A2	0.98694588	0.010804524	0.99992578
LPAR3	3.36125877	0.010904645	0.99992578
GALNT18	3.67296217	0.010994589	0.99992578
SOX4	-1.3057096	0.01102052	0.99992578
PLEKHA8P1	1.47619793	0.011209723	0.99992578
ZC3HAV1L	2.0114145	0.011692954	0.99992578
LINC02798	3.52768257	0.011779063	0.99992578
ARHGAP25	-0.996218	0.011844823	0.99992578
FAM102B	-2.226716	0.01191186	0.99992578
SPSB3	1.2311302	0.012094168	0.99992578
TERF2IP	0.93919122	0.012102737	0.99992578
RNF24	1.02028171	0.012327075	0.99992578
GPR35	2.48188404	0.012380385	0.99992578
AL390719.1	0.91127519	0.012534757	0.99992578
NDP	-1.5870514	0.012612119	0.99992578
ZNF362	0.92284283	0.012796069	0.99992578
KCTD15	2.6379265	0.013292604	0.99992578

TEX10	0.88372521	0.013320658	0.99992578
ANKRD36B	0.96668331	0.013444831	0.99992578
ECI2-DT	2.42082006	0.013695699	0.99992578
AC010969.1	1.99627049	0.013731808	0.99992578
SNX20	-0.9036514	0.01508868	0.99992578
CHST2	-1.6385539	0.015107844	0.99992578
CCDC134	1.94812712	0.015371792	0.99992578
AC022540.1	2.01274104	0.015670797	0.99992578
IL1RAP	1.21594279	0.015901534	0.99992578
RAB40B	1.97673775	0.015925752	0.99992578
ISCA1	0.85421146	0.015929769	0.99992578
FAM126B	0.80223722	0.01633735	0.99992578
HCG20	1.21336924	0.016360028	0.99992578
AC011611.3	0.78613301	0.01673902	0.99992578
KLHL23	4.05319723	0.016920481	0.99992578
CPNE8	0.768893	0.017205879	0.99992578
SGMS1	0.77161056	0.017579247	0.99992578
ATL1	1.40796762	0.017707181	0.99992578
CPT1A	0.85168609	0.018341894	0.99992578
IER3	1.13375693	0.018373596	0.99992578
FSIP1	4.33303255	0.018492074	0.99992578
RIPK3	0.93502628	0.018713076	0.99992578
P4HA2	0.8340686	0.018729788	0.99992578
RIMS3	1.99137182	0.018764751	0.99992578
SEMA6B	2.49642847	0.01910825	0.99992578
CDC42EP3	0.73017936	0.019552725	0.99992578
PDE4D	2.07961494	0.019641778	0.99992578
SCAMP1	0.74811097	0.019732882	0.99992578
INHBA	0.71347643	0.019810223	0.99992578
OSGIN1	0.86676103	0.020001043	0.99992578
SLCO4A1-AS	1.21149028	0.02001343	0.99992578
AC037198.2	3.68029981	0.020057734	0.99992578
FRMD6	1.32053097	0.020150173	0.99992578
STK26	0.83808651	0.020676674	0.99992578
TENT5A	-0.6998212	0.020847125	0.99992578
HSPA2	2.26490066	0.02133295	0.99992578
GXYLT1	0.71362589	0.021377396	0.99992578
ANKRD63	-4.8130673	0.021665308	0.99992578
AC015802.1	1.36342457	0.02208831	0.99992578
USP38	0.78714761	0.022267132	0.99992578
AC092919.3	2.00320712	0.022454918	0.99992578
IL1B	2.32678538	0.02246859	0.99992578
SOCS2	2.18523437	0.022566741	0.99992578
FAM78A	-1.292841	0.022849012	0.99992578
NQO1	0.75302907	0.022946449	0.99992578
SLC2A3	0.68709459	0.023053218	0.99992578
VAMP1	0.87471147	0.023310956	0.99992578
XYLT1	1.21726317	0.0235352	0.99992578
AC018755.4	-1.6836388	0.023747583	0.99992578
TXLNG	0.83585264	0.024629562	0.99992578
ZNF710	-1.3565415	0.024870196	0.99992578
DCBLD1	0.91743	0.024983836	0.99992578
E2F7	2.4448355	0.025441426	0.99992578
PAG1	1.20484145	0.02552353	0.99992578
HSPH1	1.42937785	0.025551397	0.99992578
TSKU	1.14010941	0.025729353	0.99992578
EHD1	0.64344707	0.026150051	0.99992578
IER3-AS1	1.3405647	0.026160456	0.99992578
CEBPB	-1.5002433	0.026292192	0.99992578
AC106782.1	3.00502043	0.026421656	0.99992578
THBS1	4.00417377	0.026501207	0.99992578
RNF216P1	0.86362434	0.026540014	0.99992578
BRMS1L	0.7979905	0.026553108	0.99992578
ADGRE4P	-1.1452732	0.026595403	0.99992578
HHEX	-0.9740597	0.026650655	0.99992578
TMCC1-AS1	-2.1589887	0.026750908	0.99992578
DGKE	1.30742638	0.026757678	0.99992578
AL157955.1	-0.9832318	0.027035632	0.99992578
PPP2R5B	1.30377037	0.027470047	0.99992578
SMOX	0.74416568	0.027625676	0.99992578
PGM3	0.78917616	0.027859697	0.99992578
LGALSL	-1.8599763	0.028048568	0.99992578
CDAN1	1.22226019	0.028199019	0.99992578
OVOL1	0.99955666	0.028233829	0.99992578
MFSD5	0.7113763	0.028371354	0.99992578
CCNA1	0.77313003	0.028841552	0.99992578
RAPGEFL1	2.26656942	0.029288476	0.99992578
F2RL1	1.95578744	0.029732074	0.99992578
ERO1B	0.85056518	0.03002426	0.99992578
MYCL	-0.9712111	0.030074729	0.99992578
PXYLP1	0.96165859	0.030197659	0.99992578
KIT	3.65538417	0.030246416	0.99992578
MYC	-0.6771493	0.030341059	0.99992578
KIAA1324L	3.4945665	0.030449554	0.99992578
PALLD	-0.6130998	0.030493516	0.99992578
BRI3	-1.0702867	0.031244739	0.99992578
ATAD2B	0.85204338	0.031365563	0.99992578
DNAJB6	0.70952712	0.031505804	0.99992578
LINC01355	-1.4174467	0.031550944	0.99992578
VLDLR	2.1890496	0.031616619	0.99992578
NR2C1	0.73213802	0.032888508	0.99992578
TOE1	0.71817722	0.033077418	0.99992578
MAP3K20	0.67517916	0.033720691	0.99992578
MSC-AS1	0.59867281	0.034170703	0.99992578
MAP3K5	-0.9284627	0.034612514	0.99992578
BLOC1S4	-0.9305143	0.035331549	0.99992578
CPEB4	0.67420646	0.035568815	0.99992578
CABLES1	-2.2878782	0.035724707	0.99992578
GOLGA7B	-1.8699372	0.035758281	0.99992578

CCL2	0.73398925	0.035941774	0.99992578
CDKN1A	0.58199943	0.036175418	0.99992578
MSC	0.64561014	0.03640647	0.99992578
PALM2AKAP2	0.96229304	0.036524	0.99992578
LPAR6	0.60716504	0.036525813	0.99992578
HCAR3	1.30682857	0.036842797	0.99992578
SLC30A1	1.23465857	0.037039496	0.99992578
IKZF1	-0.7444691	0.037168315	0.99992578
C9orf139	-1.3235233	0.037445343	0.99992578
RNF216	0.6541953	0.037750641	0.99992578
SNX9	0.58077709	0.038246596	0.99992578
CREB3L4	1.13051472	0.038979605	0.99992578
PHLDA1	0.71548077	0.03900499	0.99992578
RBMXL1	0.95397913	0.039051198	0.99992578
SLC25A38	0.75465901	0.039369489	0.99992578
SLC2A1	1.0439141	0.039664436	0.99992578
OSER1	0.84436194	0.039786425	0.99992578
CPEB3	-1.5658943	0.040002007	0.99992578
LINC01890	-0.8504324	0.040462601	0.99992578
CACYBP	0.61868617	0.040510816	0.99992578
CHPF	1.39029472	0.040813606	0.99992578
PHF8	1.03984332	0.040946894	0.99992578
RASL11A	1.66974255	0.041271483	0.99992578
ITGB7	0.89308023	0.041299713	0.99992578
BAG3	0.96744576	0.04138543	0.99992578
LRIG1	1.172003	0.041450891	0.99992578
SNUPN	-0.8463555	0.041606857	0.99992578
TNIK	0.86076238	0.041651298	0.99992578
AL353708.3	1.28942779	0.041686768	0.99992578
TAF1	0.94278003	0.041756811	0.99992578
RGS19	-0.5873672	0.042364096	0.99992578
SLCO4A1	0.76451009	0.042545324	0.99992578
TIMM8A	-0.9113	0.04280194	0.99992578
ZFAND2A	0.89174158	0.042849793	0.99992578
AL021707.5	-1.5232115	0.042961407	0.99992578
MT1JP	5.4969265	0.043050693	0.99992578
IL7	0.99388131	0.04347366	0.99992578
SPTY2D1OS	0.86612625	0.043489294	0.99992578
UBA2	0.87381597	0.043740251	0.99992578
MDFIC	-1.0359294	0.043764744	0.99992578
CERK	-0.9030231	0.044003832	0.99992578
SBDS	0.62482693	0.044722793	0.99992578
LINC00539	-1.3782209	0.045000611	0.99992578
SLC16A6	1.14502133	0.045103766	0.99992578
NEK4	0.59431509	0.045143063	0.99992578
DNAJA1	0.90013057	0.045269414	0.99992578
ICAM5	1.17680014	0.045287269	0.99992578
PPP2R5D	0.72200643	0.045502632	0.99992578
EPS15L1	0.82228762	0.045817973	0.99992578
HBEGF	0.77235237	0.045975405	0.99992578
TRIM36	0.93516513	0.046138745	0.99992578
FOXN3-AS1	-2.4589645	0.046195012	0.99992578
TRG-AS1	-0.9622248	0.046363224	0.99992578
SMAP2	1.00807907	0.046410722	0.99992578
ZBTB43	0.53795719	0.046510622	0.99992578
SGPP1	-0.9391693	0.046950833	0.99992578
PITPNC1	0.5859357	0.047021775	0.99992578
RNF217	-0.8970429	0.047450158	0.99992578
VEGFA	1.12737604	0.047498415	0.99992578
AHRR	1.95359239	0.047558261	0.99992578
HSPD1	0.70463404	0.047990628	0.99992578
TMEM170B	-1.4130876	0.048369996	0.99992578
NRBP1	0.52573415	0.048371844	0.99992578
FLT3	1.62588554	0.04891057	0.99992578
CDK5RAP2	0.55104107	0.04892948	0.99992578
FAM124A	1.13734884	0.049079174	0.99992578
DLEU1	-1.073452	0.049193223	0.99992578
MCM6	0.76303184	0.049306601	0.99992578
ARHGAP5	0.86520865	0.049441581	0.99992578
PDGFB	0.98342627	0.049574061	0.99992578
LMO2	-1.6217508	0.049592276	0.99992578
BRD4	-1.1088274	0.04977488	0.99992578
ZNF823	0.8019172	0.049841053	0.99992578
RPARP-AS1	-1.2026486	0.049842193	0.99992578
AIFM2	0.52103138	0.049905041	0.99992578

Table.S4

Genes by 12,13-diHOME alone compared to DMSO at 8-hour

Gene	log2FC	Unadjusted P	Adjusted P
GALNT18	4.35231379	1.58E-06	0.012354959
ATP6V1H	0.41232555	2.36E-06	0.012354959
LPAR2	0.83758331	3.10E-06	0.012354959
TIMP2	-0.4094372	5.85E-06	0.013099845
EREG	2.71291784	6.55E-06	0.013099845
PDLIM2	-0.7207847	6.80E-06	0.013099845
SERPINB2	2.69149865	7.67E-06	0.013099845
HGD	1.02479587	1.51E-05	0.022636585
STAG3	-0.6808309	1.95E-05	0.025952365
MAP1LC3B	0.27085539	2.38E-05	0.028459494
GPR68	1.94691736	3.35E-05	0.036376052
NQO1	0.9329612	4.44E-05	0.040192985
DIPK1A	0.7587444	4.55E-05	0.040192985
GARRE1	0.65497375	4.70E-05	0.040192985
EVI5	0.41823374	5.34E-05	0.04258491
CHMP4C	0.48158378	6.74E-05	0.046604843
SLC17A5	0.3673316	6.85E-05	0.046604843
CDKN1A	0.4992277	7.01E-05	0.046604843
LRG1	2.13976954	7.69E-05	0.047130001
TRIM39	0.30128656	7.88E-05	0.047130001

Table.S5

Genes by combinatorial treatment (diHOME + Pnut) compared to DMSO at 8-hour

Gene	log2FC	Unadjusted P	Adjusted P
TDP2	0.794021899	2.07E-06	0.012780547
LRG1	1.859471849	2.14E-06	0.012780547
CYP1A1	4.476561626	4.97E-06	0.0159944
CREB5	2.241951759	8.23E-06	0.0159944
FAM124B	-2.257347703	9.66E-06	0.0159944
MYL12B	0.229637405	9.99E-06	0.0159944
RPS19BP1	0.36320878	1.04E-05	0.0159944
RAD51D	-1.165015016	1.20E-05	0.0159944
GCLM	3.143909741	1.21E-05	0.0159944
USP16	0.607215646	1.43E-05	0.0159944
HSPA6	7.521842836	1.54E-05	0.0159944
NSMCE2	0.663547788	1.69E-05	0.0159944
CFAP99	2.342107793	1.86E-05	0.0159944
URB2	-1.03155722	1.87E-05	0.0159944
NQO1	0.682549688	2.16E-05	0.017219025
TNFSF9	3.212717498	2.31E-05	0.017239161
PHACTR1	1.414156847	2.76E-05	0.017802444
H2BC11	3.436546345	2.81E-05	0.017802444
IL10RB	-1.211024981	2.97E-05	0.017802444
SDHB	0.354822212	3.00E-05	0.017802444
TXN	1.087724156	3.13E-05	0.017802444
SRXN1	2.822811274	3.36E-05	0.017943688
RILPL2	0.637906213	3.74E-05	0.017943688
DUSP4	2.597302544	3.88E-05	0.017943688
PIK3CD	-0.727057571	3.98E-05	0.017943688
ATP6V0E1	0.355856378	4.00E-05	0.017943688
TMEM59	0.271131021	4.05E-05	0.017943688
ADM	2.337333345	4.28E-05	0.018280616
EIF1B	1.343247273	4.48E-05	0.018484565
SNRPD2	0.330424242	4.94E-05	0.019475796
GSTM3	2.05784616	5.05E-05	0.019475796
EREG	2.866331787	5.74E-05	0.020096359
CES2	-0.71403644	5.83E-05	0.020096359
UBE3D	0.408501072	5.93E-05	0.020096359
CLDND1	1.230691169	6.08E-05	0.020096359
VAMP1	1.794544293	6.20E-05	0.020096359
CCL20	4.243148025	6.22E-05	0.020096359
YPEL5	0.801031252	6.91E-05	0.021173455
FCAR	1.889089727	6.93E-05	0.021173455
TBC1D15	1.033486585	7.08E-05	0.021173455
INTS2	-0.819833369	7.87E-05	0.022357909
RIT1	1.613953177	7.87E-05	0.022357909
KCTD20	0.391518939	8.04E-05	0.022357909
ETS2	1.342196937	8.55E-05	0.022622829
OSER1	1.942556098	8.57E-05	0.022622829
CCDC93	0.622216648	8.72E-05	0.022622829
TRIM5	0.7988776	9.68E-05	0.022622829
CUL9	-0.691965344	9.80E-05	0.022622829
CRYM	2.318165387	9.83E-05	0.022622829
GALNT18	3.791699018	9.85E-05	0.022622829
COX17	0.705032962	0.000100815	0.022622829
RPL36AL	0.558365788	0.000101745	0.022622829
DPYSL3	2.178343504	0.000103538	0.022622829
SIGLEC9	-0.704502609	0.000104623	0.022622829
ATF4	1.010461497	0.000104931	0.022622829
ZNF800	0.417736341	0.000107339	0.022622829
PSMA3	0.868067705	0.000107818	0.022622829
RNF13	0.360645314	0.000111795	0.023052886
AIFM2	1.048670095	0.000117313	0.023092936
PSMC6	1.112298062	0.000119432	0.023092936
CDKN1A	1.192404313	0.000121718	0.023092936
ANKRD27	-0.391234099	0.000127447	0.023092936
BPGM	0.614062259	0.000127806	0.023092936
CHMP5	0.623427746	0.000129186	0.023092936
PKN3	-0.716029856	0.000130157	0.023092936
ARRDC4	2.109178105	0.00013161	0.023092936
LANCL1	-0.566300432	0.000132695	0.023092936
PPA2	0.278812584	0.000132761	0.023092936
MEI1	0.545478067	0.000135365	0.023092936
MT1X	3.466173944	0.00013574	0.023092936
IER3	1.546444636	0.000139393	0.023092936
ABTB2	1.700779153	0.000140877	0.023092936
CASP4	1.090007746	0.000140952	0.023092936
TRIM16	1.437923521	0.000144753	0.023156552
CDKN1C	4.065221114	0.000146194	0.023156552
PRDX1	0.549878581	0.000148001	0.023156552
AKR1B10	5.559569954	0.000150777	0.023156552
CLUAP1	-1.085184847	0.000151021	0.023156552
MT1G	3.598495992	0.000153416	0.023226032
RPS20	0.55033623	0.000159393	0.023390096
FRG1	0.289883473	0.000159672	0.023390096
HAVCR1	1.879850051	0.000160367	0.023390096
UFC1	0.504320616	0.000163135	0.02350713
UBE2D1	0.642652224	0.000165653	0.023585761
ANXA1	0.96982442	0.000168698	0.023587626
HBEGF	2.275106859	0.00016961	0.023587626
NUFIP2	0.661254595	0.000179991	0.024692167
GABARAPL2	0.377084134	0.000184052	0.024692167
CCDC30	1.986579179	0.000184114	0.024692167
NDE1	-0.847175574	0.00019336	0.024692167
NCAPD3	-0.489060042	0.000196358	0.024692167
SNX9	0.868831061	0.000197006	0.024692167
IL1A	2.387082003	0.000197523	0.024692167
TUBGCP6	-0.544344376	0.000197549	0.024692167
DDIT4	1.636727286	0.000198116	0.024692167
C2CD5	-0.85270064	0.000198198	0.024692167

ZFAND2A	4.478152481	0.000200643	0.024739107
KTN1	0.476456845	0.000208965	0.024846628
HINT1	0.650984053	0.000210214	0.024846628
DIPK2A	-0.740114094	0.000211983	0.024846628
TMPO	-0.582075443	0.000213267	0.024846628
ATP6V1F	0.281413733	0.000216956	0.024846628
HSPA1B	4.901569613	0.000218252	0.024846628
MTHFD2L	1.021102483	0.000222092	0.024846628
DNTTIP2	1.266474204	0.000223475	0.024846628
P4HA2	0.587007207	0.000224738	0.024846628
ZNF485	-1.69651886	0.000226154	0.024846628
PPP2R3C	0.518697377	0.000227875	0.024846628
DHODH	-1.183865736	0.000231563	0.024846628
ABCB5	1.872144975	0.000231668	0.024846628
SEM1	0.716105663	0.000240188	0.024846628
OCIAD1	0.20171838	0.000240825	0.024846628
H2AC6	1.533080943	0.00024248	0.024846628
PXDC1	0.859048087	0.000243345	0.024846628
WAC	0.718595066	0.000249123	0.024846628
BCS1L	-0.920564435	0.000252989	0.024846628
HUS1	0.610831877	0.000254616	0.024846628
BCL9L	1.424037283	0.000256565	0.024846628
HTRA3	4.923237799	0.000256646	0.024846628
H3C6	1.505275027	0.00025708	0.024846628
GBE1	1.049571877	0.000257815	0.024846628
HDAC7	-0.977221595	0.000263602	0.024846628
IKBKB	-0.703120917	0.000264686	0.024846628
ANKFY1	-0.583294732	0.000264916	0.024846628
ZFP90	-1.004003481	0.000268091	0.024846628
MYBBP1A	-0.583585208	0.00027282	0.024846628
DDHD1	0.977260731	0.000275409	0.024846628
KCNE1B	1.484454331	0.000277551	0.024846628
TIPARP	2.190220135	0.000281158	0.024846628
HSDL2	0.286426633	0.000283597	0.024846628
CYSTM1	0.30569483	0.000284041	0.024846628
ZNF691	0.892377599	0.000286133	0.024846628
ZNF710	-1.62755639	0.000286444	0.024846628
SLTM	0.306076877	0.000289486	0.024846628
NDOR1	-0.489381297	0.00028966	0.024846628
MAP1LC3B2	1.458648394	0.000289875	0.024846628
MMACHC	0.504596457	0.000290221	0.024846628
TXNRD1	1.201586691	0.000291208	0.024846628
DHX57	-0.793009908	0.000291598	0.024846628
POMP	0.697431844	0.000294555	0.024846628
NSUN3	1.449892739	0.000296578	0.024846628
PSRC1	-2.292431546	0.000297347	0.024846628
CAMTA1	0.419048214	0.00029746	0.024846628
PTH2R	3.709394262	0.000305958	0.024846628
PHF1	1.450805739	0.000308846	0.024846628
MT2A	2.492149777	0.00030931	0.024846628
MPC2	0.430292289	0.000313664	0.024846628
ARHGAP25	-1.598921527	0.000314431	0.024846628
CILP2	2.357214533	0.000315138	0.024846628
FAM161B	0.572868104	0.000315905	0.024846628
ATP6V1G1	0.885447791	0.000317063	0.024846628
DENND4B	-0.808329654	0.000319232	0.024846628
SOD1	1.080295773	0.0003229	0.024846628
PCSK7	-0.510675674	0.000326166	0.024846628
SSX2IP	-0.520590074	0.000326957	0.024846628
SNW1	0.569375033	0.000328324	0.024846628
CRYAB	5.102706835	0.000329608	0.024846628
RRN3	0.83292983	0.000332826	0.024846628
LOXL3	-0.632856517	0.000334664	0.024846628
SERTAD1	1.3190044	0.000335504	0.024846628
INPP5K	0.590747999	0.000335561	0.024846628
RAB3D	-0.902424274	0.000336551	0.024846628
TMEM135	0.859462035	0.000342479	0.025129128
IL36B	3.066437213	0.000346927	0.025137065
TIAM2	-0.764866295	0.000347131	0.025137065
CCL3	1.713701394	0.000348892	0.025137065
CXCR5	1.616668134	0.000358656	0.02546353
CYTH4	-0.618096417	0.000360959	0.02546353
ADK	0.698857041	0.00036135	0.02546353
BANP	1.523096193	0.00036194	0.02546353
BNIP3	1.05980175	0.000369648	0.025725899
BRCA2	1.471055628	0.000369971	0.025725899
CEBPZ	0.470340725	0.000374739	0.02590681
EMC3	0.83212641	0.000380184	0.026132194
VGLL4	-0.809148074	0.000387079	0.026454109
SUCLG1	0.445833957	0.000396597	0.026950595
OSGIN1	2.350935651	0.000399797	0.027007594
RAB3GAP2	-0.19615036	0.000406279	0.027007594
DUS2	-0.569580667	0.000410002	0.027007594
SKP2	-1.174974379	0.000411782	0.027007594
DEPDC5	-0.672241384	0.000412685	0.027007594
SKP1	0.637981485	0.000412976	0.027007594
TBCA	0.891553793	0.000415264	0.027007594
CSRNP1	2.277358835	0.000417921	0.027007594
FLI1	-1.491832503	0.000421518	0.027007594
TNPO1	1.050605963	0.000424133	0.027007594
H2BC4	2.551916469	0.000426786	0.027007594
POU2F2	-2.044127678	0.000429779	0.027007594
PELO	1.840162895	0.000430905	0.027007594
SHF	2.42381557	0.000432458	0.027007594
MT1A	5.147620203	0.000436186	0.027007594
PNPLA8	1.461261704	0.000437485	0.027007594
TALDO1	0.566863188	0.000438818	0.027007594
TBC1D7	1.064180053	0.000440577	0.027007594
CEP164	-0.622297684	0.000441708	0.027007594

ARID5A	0.661402079	0.000442599	0.027007594
HSPA1A	3.998916409	0.00044713	0.027145536
COX6B1	0.262391367	0.000450302	0.027154233
NACA	0.378210362	0.000451814	0.027154233
TNRC18	-0.669319072	0.000459314	0.027260735
PSMA4	0.76695915	0.000459887	0.027260735
MSH6	-0.351195452	0.000461712	0.027260735
TMEM70	0.608470507	0.00046684	0.027260735
HMOX1	2.936429132	0.000470911	0.027260735
NDUFA4	0.477611963	0.000471686	0.027260735
SEC61B	0.705082813	0.000477555	0.027260735
TMCO1	0.31249581	0.000477948	0.027260735
CDC25A	-1.418208218	0.000479706	0.027260735
DNAJC18	1.236996678	0.000480481	0.027260735
ZNF330	0.59452981	0.000483738	0.027260735
ALDOA	1.188144447	0.000488401	0.027260735
CAD	-1.307489156	0.00049011	0.027260735
KIF24	0.848423743	0.000490792	0.027260735
SDF2	0.628935342	0.000492183	0.027260735
C1orf21	-0.500464533	0.000494563	0.027260735
LRRC8E	1.629479166	0.000495884	0.027260735
MRNIP	1.230589279	0.000498365	0.027260735
TRIM66	-1.83960012	0.000500328	0.027260735
SPIN1	0.334042614	0.000501537	0.027260735
MZT2A	-0.225575827	0.000504204	0.027260735
ANAPC7	-0.422979467	0.000505694	0.027260735
ABCA5	0.508482463	0.000511458	0.027260735
C1D	0.38167967	0.000519406	0.027260735
ARL10	-0.631309699	0.000527816	0.027260735
HRH2	-1.847982854	0.000530451	0.027260735
RECQL5	-0.596522437	0.000534777	0.027260735
HGD	1.320765635	0.000536467	0.027260735
SVBP	0.902333178	0.000537015	0.027260735
CCL3L3	2.099388831	0.000542608	0.027260735
IFTAP	0.666007062	0.000544256	0.027260735
MMP1	3.906982698	0.000546907	0.027260735
MLLT11	2.586537907	0.000548021	0.027260735
RIN2	-2.357926105	0.000548924	0.027260735
KCMF1	1.094812065	0.000550117	0.027260735
SMYD3	0.212792705	0.000551862	0.027260735
ARHGAP1	-0.907187237	0.000552534	0.027260735
IRAK2	1.363643491	0.000553735	0.027260735
RABL2B	-0.647211534	0.000556611	0.027260735
TXNL1	1.418648293	0.000558785	0.027260735
LRRC3	-0.958347025	0.000559403	0.027260735
PIM1	1.79910845	0.000560011	0.027260735
NDUFS5	0.307172046	0.000560868	0.027260735
RTN3	0.472503988	0.000561961	0.027260735
CDC42SE1	0.850424898	0.00056382	0.027260735
ST3GAL5	-0.494453437	0.000564504	0.027260735
UBL5	0.515179638	0.000568106	0.027260735
AGBL5	0.317469924	0.000569726	0.027260735
PANK3	0.625671353	0.000570723	0.027260735
ERMAP	0.713084605	0.000572273	0.027260735
INKA2	-1.163656149	0.000573789	0.027260735
ZBTB1	0.642438403	0.000574695	0.027260735
CMC2	0.480341937	0.000576908	0.027260735
TPT1	0.832541506	0.000577697	0.027260735
PFDN2	0.720334059	0.000581817	0.027260735
MRPL17	-0.432387234	0.000583765	0.027260735
SBDS	0.769224123	0.000588756	0.027260735
ZNF143	1.110231021	0.000594118	0.027260735
SLC15A5	2.082509107	0.000597581	0.027260735
CDK5RAP2	1.5054532	0.000600017	0.027260735
NOP56	-0.405048748	0.000607852	0.027260735
EPC2	0.452336759	0.000611031	0.027260735
UBAP1	1.182300218	0.000612766	0.027260735
DNAJB1	3.53356498	0.000613451	0.027260735
XPC	-0.793041388	0.000613455	0.027260735
SERPINB2	4.018446075	0.000613978	0.027260735
RAB18	0.798990822	0.000620751	0.027260735
KLHL6	0.786490464	0.000622406	0.027260735
PLEKHO2	-0.446050697	0.000624915	0.027260735
TNFSF18	3.56277343	0.000627781	0.027260735
TRIT1	-0.534630298	0.000629245	0.027260735
OSTM1	0.274692573	0.000632494	0.027260735
TICRR	-1.289136844	0.000634637	0.027260735
BLVRB	0.718862028	0.000636028	0.027260735
CYB5R4	0.800898139	0.000637049	0.027260735
MTR	-0.539400392	0.000637937	0.027260735
PTPN18	-0.618948066	0.000639383	0.027260735
LBH	2.462849472	0.000646716	0.027260735
ZNF772	-1.074657199	0.000648116	0.027260735
ANGPTL4	2.241809626	0.000649505	0.027260735
PSMA6	1.092452462	0.000650669	0.027260735
PGBD5	-2.099247649	0.000651617	0.027260735
CPSF1	-0.380562123	0.000652907	0.027260735
MMS22L	-0.811159939	0.000655516	0.027260735
TNF	1.426975452	0.000657892	0.027260735
ERRFI1	2.006134353	0.000661819	0.027260735
SPTBN4	2.969077685	0.000663262	0.027260735
COPS5	0.38597546	0.00066842	0.027260735
FRMD6	0.923174192	0.000674654	0.027260735
FTCD	4.466657886	0.000675247	0.027260735
CDC42EP3	0.874115511	0.000675684	0.027260735
STK17A	1.226926819	0.000677535	0.027260735
GCNT1	-1.234597779	0.000677551	0.027260735
USP38	1.345642838	0.000678726	0.027260735
GFI1B	0.977015978	0.000678983	0.027260735

SEC61G	0.799436417	0.000680389	0.027260735
MORC3	1.098028621	0.000680513	0.027260735
FBF1	-1.116892027	0.000684806	0.027260735
MOV10	-0.63306755	0.000685732	0.027260735
STYX	-0.470154799	0.000686122	0.027260735
KIF1B	0.691454886	0.000688344	0.027260735
PSMC2	0.522152967	0.000688863	0.027260735
COPE	0.29300737	0.000691035	0.027260735
DNAJB4	2.612337327	0.000691941	0.027260735
RB1CC1	1.189029309	0.000693259	0.027260735
CRY1	1.281134859	0.000695194	0.027260735
ZNF888	-0.712731107	0.00069998	0.027287769
SLC12A8	2.24213576	0.000700447	0.027287769
SQSTM1	1.258967669	0.000708092	0.027496023
SNAPC2	-0.560683097	0.000711971	0.02750887
MKLN1	0.572995506	0.000714361	0.02750887
CLCF1	1.707510972	0.000715323	0.02750887
NBPF1	-0.728228922	0.000717823	0.027516531
URB1	-1.045478998	0.000725161	0.027680018
CHIC2	1.069129121	0.000727583	0.027680018
MED4	0.351272886	0.000729031	0.027680018
DNAH3	4.866443334	0.000736199	0.027863732
RNF227	-0.95878933	0.000744032	0.028071359
PCGF5	0.582233885	0.000752501	0.028301616
ANKRD12	0.988354067	0.000759206	0.028464283
CCDC88C	-1.192238798	0.000768761	0.028732449
PLXNB2	-0.897349696	0.000772486	0.02876155
OXER1	-1.067471719	0.000774349	0.02876155
SLC38A2	0.588339378	0.000780587	0.028903483
FTL	0.348286527	0.000785077	0.028925192
MT1E	3.160593145	0.000786011	0.028925192
SLC7A11	1.391838682	0.000790008	0.028983106
TSPAN13	0.924392034	0.000793181	0.02901052
NPLOC4	0.81072193	0.000796292	0.029035544
TBC1D14	-1.0693875	0.000799473	0.02904095
ALAS1	1.192327291	0.000802319	0.02904095
TFPI	1.140479481	0.000803725	0.02904095
SIGLEC14	-1.219344359	0.000813592	0.0291685
EEF2K	-1.680997338	0.000814191	0.0291685
COQ10B	0.929987052	0.000816523	0.0291685
CWF19L1	0.406236926	0.000818708	0.0291685
RPS13	0.498808994	0.00081945	0.0291685
NEU1	0.618394474	0.000830961	0.029453621
RNF24	0.969629424	0.000835955	0.029453621
PPP1R15B	1.208689172	0.000836117	0.029453621
FAM114A1	-0.753087094	0.000838323	0.029453621
CAMKK2	-0.898277402	0.000841053	0.029453621
PROK2	3.334017455	0.00084466	0.029453621
PSMB7	1.255796201	0.000844698	0.029453621
ADGRE5	-0.747419629	0.000856964	0.029740687
AKR1C1	3.758740191	0.000857904	0.029740687
IKZF1	-1.398686033	0.000876739	0.030305775
ZNF671	-1.138932674	0.000881118	0.030369363
PKMYT1	-1.138447806	0.000893975	0.030623604
RSL24D1	0.814910859	0.000900639	0.030623604
FAM177A1	0.566495026	0.000901424	0.030623604
E2F6	1.134863824	0.000902616	0.030623604
EID3	1.844900838	0.000909269	0.030623604
MAGOH	0.519999901	0.000909811	0.030623604
SSR2	0.552282727	0.000910846	0.030623604
STOX2	2.425131495	0.000911207	0.030623604
VEGFA	1.756108011	0.000911539	0.030623604
ZFP30	-1.083561839	0.000914566	0.030639251
SLC3A2	0.685056519	0.000927869	0.030765969
SBK3	4.116518077	0.000929253	0.030765969
GMPR2	0.238608863	0.00092988	0.030765969
PIGS	-0.608134408	0.0009303	0.030765969
SH3BP2	-0.525285969	0.000931211	0.030765969
UNC13C	3.036575782	0.000939241	0.030870267
STARD3NL	0.513533773	0.000940407	0.030870267
NLE1	-1.197536782	0.000949581	0.030870267
ACP6	-1.107626614	0.000952889	0.030870267
EGF	2.843700587	0.000955533	0.030870267
TAX1BP1	0.686325919	0.000956635	0.030870267
TVP23B	0.863442164	0.000957981	0.030870267
MTSS1	-0.978254755	0.000961599	0.030870267
GATA2	4.623931585	0.000968889	0.030870267
SARAF	0.406439408	0.000972387	0.030870267
EGLN3	2.323417571	0.000972957	0.030870267
ANXA5	0.359458095	0.000973926	0.030870267
LPXN	0.56610559	0.000977784	0.030870267
SEC62	0.467353901	0.000977845	0.030870267
AGO2	0.708615855	0.000979759	0.030870267
BTBD11	2.065007644	0.000980273	0.030870267
DDX42	-0.507191491	0.000980827	0.030870267
ADM2	1.030006296	0.000984421	0.030870267
TRAF2	0.497232966	0.000984577	0.030870267
SRP14	0.454297834	0.000987197	0.030870267
DCTN6	0.606005936	0.000988571	0.030870267
TSEN15	-0.406639191	0.000994295	0.030968152
RELT	1.038984203	0.001000528	0.031081329
IL21R	-1.483045497	0.001003203	0.031083707
FAH	0.399897958	0.001014912	0.031222201
CEP192	-0.887719623	0.001019082	0.031222201
FRMD4A	-1.378576406	0.001019509	0.031222201
RAB38	1.685480288	0.001020763	0.031222201
ST8SIA4	-1.863820694	0.001021275	0.031222201
FLCN	0.866704472	0.001023336	0.031222201
LMCD1	3.779600325	0.001032618	0.031425228

EHD4	-0.995685241	0.00105192	0.031931372
NSRP1	0.56039147	0.001057039	0.032005537
TANK	0.680473767	0.001061188	0.032015146
UBE2D3	0.593503257	0.001067748	0.032015146
MAFB	-1.486773531	0.001071886	0.032015146
RABL2A	-0.634078822	0.001072752	0.032015146
ZNF281	-0.367772561	0.001073031	0.032015146
SPHK1	0.740035604	0.00107549	0.032015146
USP15	0.754117747	0.001076618	0.032015146
CEP57	-0.438580623	0.001078771	0.032015146
NDEL1	0.84410465	0.001084418	0.032103067
WASF1	1.457780046	0.001092246	0.032254961
ZFAND5	1.136835218	0.001098443	0.032358084
ZC3H12D	-1.959044171	0.001105951	0.032499207
CASP2	-0.836747278	0.001110153	0.032523452
LYRM2	0.354569081	0.001112215	0.032523452
MYO1F	-0.559986375	0.001120566	0.032552905
ALDH3B1	-0.56208862	0.001123041	0.032552905
NQO2	0.889431738	0.001126921	0.032552905
ARMC7	-0.804738571	0.001128116	0.032552905
POLE4	0.594959145	0.001131862	0.032552905
CBX8	-1.538798199	0.001132362	0.032552905
IP6K2	0.596929349	0.0011368	0.032552905
PCNX3	-0.818539816	0.001139305	0.032552905
GOLPH3L	-0.787757674	0.001140213	0.032552905
PPM1F	-0.619858581	0.001145897	0.032552905
PPP2CA	0.946647781	0.001147443	0.032552905
MPHOSPH9	-0.534819776	0.001147967	0.032552905
NRDC	0.706688428	0.001150766	0.032552905
GABPB1	0.506822276	0.001151328	0.032552905
CSTB	0.304789799	0.001163313	0.032640947
THTPA	-0.767812695	0.001165425	0.032640947
TRAF3	0.592543288	0.00116739	0.032640947
DNAJA1	1.652021608	0.001168069	0.032640947
NECAB2	4.200804461	0.001170812	0.032640947
FBXO30	1.774025214	0.001172229	0.032640947
C1orf52	1.120178855	0.001173546	0.032640947
AKR1B1	1.256077288	0.001184686	0.032874352
SLC36A3	1.499327876	0.001192252	0.033007704
KLHL32	1.789834329	0.001201531	0.033187797
PRAM1	-1.055860067	0.001212361	0.033268988
ARHGAP30	-0.687323229	0.001219199	0.033268988
TFEC	0.773458093	0.001219616	0.033268988
FAM171B	3.117549141	0.001220673	0.033268988
ABL1	-0.665529086	0.001220911	0.033268988
FANCA	-0.4096282	0.001221161	0.033268988
GCNT4	1.719329256	0.001227225	0.03327052
JADE1	-0.839903143	0.001228538	0.03327052
CIR1	1.023587898	0.001232237	0.03327052
ATP8B1	-0.78850583	0.001236335	0.03327052
EPHB1	2.075243906	0.001239381	0.03327052
PIGM	-1.332617466	0.00124126	0.03327052
ATRIP	-0.901797776	0.001243988	0.03327052
PIGW	-0.749381786	0.001248376	0.03327052
CKS2	2.049243722	0.001250766	0.03327052
LIF	1.822598662	0.001253018	0.03327052
NUP93	-0.534408814	0.001255345	0.03327052
SEMA6B	2.412648579	0.001257036	0.03327052
FGFR1OP2	0.418119517	0.001257381	0.03327052
STX6	-0.823333604	0.001263236	0.033302299
RLF	1.101173021	0.001265176	0.033302299
HAGHL	-0.595227892	0.001269144	0.033302299
NUP160	-0.700314145	0.00126972	0.033302299
FXR2	1.121027445	0.001273958	0.033340353
STON2	2.989618126	0.001277689	0.033348804
C21orf58	-2.560982687	0.001279858	0.033348804
XRCC5	0.224169804	0.00128706	0.033413419
PPP1R21	-0.810943576	0.001294126	0.033413419
CDK7	0.687499218	0.001294144	0.033413419
UQCRH	0.402551665	0.001296114	0.033413419
PDRG1	0.893484301	0.001296307	0.033413419
RPL24	0.657423531	0.001300982	0.033452888
STK3	0.979186772	0.001303432	0.033452888
LGI2	-1.756425998	0.001306325	0.033455348
LYRM4	0.221990811	0.001315388	0.03345654
USP12	1.339235489	0.001319439	0.03345654
SIRT7	0.702338471	0.00132033	0.03345654
EIF1	0.892548367	0.001321923	0.03345654
KEAP1	0.870867493	0.001324682	0.03345654
MAFG	1.686647976	0.001325693	0.03345654
OCSTAMP	-1.328577885	0.001329605	0.03345654
STK36	-0.771048002	0.001337341	0.03345654
MYNN	0.718165407	0.001339672	0.03345654
ATP6V1C1	0.708195969	0.001341388	0.03345654
IL6ST	0.733162941	0.001343405	0.03345654
NBPF14	-0.447427075	0.001343653	0.03345654
CFLAR	0.503365255	0.001347011	0.03345654
PSMD14	1.114315221	0.001347648	0.03345654
PSMD12	1.202081378	0.001348332	0.03345654
FNIP2	0.850552085	0.001354036	0.033488706
JPT1	-0.449579607	0.001357514	0.033488706
DPYSL2	-0.529739815	0.001358859	0.033488706
H6PD	-0.566115357	0.001364037	0.033488706
PDCL	1.194029182	0.001366282	0.033488706
TOX2	2.057918315	0.001369584	0.033488706
NSMAF	0.589877249	0.001375572	0.033488706
CDC5L	0.410921325	0.001376959	0.033488706
SRGN	0.652747232	0.001377012	0.033488706
RCAN1	1.739646065	0.001377629	0.033488706

TANGO6	-0.529203141	0.00138072	0.033495762
CDS2	0.408544871	0.001386476	0.033498151
PLA2G4C	0.748115938	0.001388967	0.033498151
ATP6V1E1	0.561653957	0.001389221	0.033498151
C15orf40	0.334237505	0.001394821	0.033534393
KANSL3	0.62724402	0.001398386	0.033534393
BAMBI	2.681385543	0.001399136	0.033534393
ITPRIPL1	-1.195576684	0.001408682	0.033631486
TSGA10	1.353285476	0.001408811	0.033631486
TONSL	-1.094255212	0.001415415	0.033664911
SRP54	0.412759217	0.00141584	0.033664911
NAB2	-0.578026005	0.001427135	0.033849757
DNAJB9	1.650659339	0.001429316	0.033849757
PPIF	0.775993706	0.001432105	0.033849757
HMGA2	2.346972858	0.001439127	0.033880973
PHF5A	0.674907373	0.001439736	0.033880973
ATP6V1H	0.593412336	0.001445343	0.033880973
CCDC121	-1.063832889	0.001449032	0.033880973
SLC30A5	0.427601025	0.001449466	0.033880973
GPR18	-0.638612527	0.001450423	0.033880973
BRF2	1.784876794	0.001453924	0.033896556
ABCC10	-0.447426269	0.001456854	0.033898785
FKBP4	1.978872515	0.001464775	0.034006803
ATAD5	-0.879156488	0.001467183	0.034006803
BARD1	-1.326181515	0.00148451	0.034331948
ATP6V1D	0.609477628	0.001488005	0.034331948
MRI1	-0.725664726	0.001493872	0.034331948
CMTM8	1.16700497	0.001495357	0.034331948
OSBPL7	-2.05761614	0.001495564	0.034331948
PPP1R15A	1.987369782	0.001508529	0.034468592
TTC1	0.8975818	0.001509998	0.034468592
EIF5	1.160234094	0.001510184	0.034468592
COG5	0.385259163	0.001513044	0.034468592
PMPCB	0.194891433	0.001519455	0.034548814
SIVA1	-0.513491853	0.001525068	0.034590048
ACOT11	-0.715510426	0.001528189	0.034590048
AFF4	1.368239351	0.001531312	0.034590048
ETFDH	0.43994085	0.001532837	0.034590048
ATG12	0.600768498	0.001551013	0.034629055
DNAJC6	2.16291171	0.001556421	0.034629055
EFHB	1.291375431	0.001558516	0.034629055
ARL8B	0.518846447	0.001559116	0.034629055
CHMP2B	0.905197449	0.001560466	0.034629055
ATG5	0.825321178	0.001562923	0.034629055
DNAJA4	3.162828444	0.001566215	0.034629055
ME1	0.821310758	0.001568609	0.034629055
NEK3	-1.254342604	0.001572399	0.034629055
SEPHS2	0.486787291	0.001572547	0.034629055
ABHD3	0.780635789	0.001573905	0.034629055
GMIP	-0.51860793	0.001580033	0.034629055
GIMAP7	-2.55153257	0.001582173	0.034629055
CAPN3	-1.021210169	0.001585036	0.034629055
GOLGA4	0.782162497	0.001585573	0.034629055
C11orf58	0.310270423	0.001587306	0.034629055
BIN1	0.458185246	0.001589336	0.034629055
ADAT1	-0.631071117	0.001591303	0.034629055
ZNF641	-0.681221447	0.001592188	0.034629055
SAMD8	0.763158836	0.001592473	0.034629055
GAPDH	0.560350345	0.001596611	0.034656029
MRPL50	0.481259437	0.001601914	0.034708147
PAIP2	0.538733088	0.001607966	0.034776255
CHORDC1	2.555875491	0.001622295	0.035022829
PLEKHM1	0.812813816	0.001625839	0.035036103
MSTO1	-0.751923276	0.001632246	0.035061237
RORA	1.569761874	0.00163781	0.035061237
LACTB2	0.495700144	0.001637923	0.035061237
PFDN1	0.390597298	0.001638732	0.035061237
PRELID3B	0.370254847	0.001643073	0.035091353
CCDC170	-1.122277796	0.001647149	0.03511569
COPS2	1.061959989	0.001653178	0.035118693
LYPD1	-1.514618122	0.001653398	0.035118693
SMAP2	0.47169629	0.001656099	0.035118693
NOSIP	0.669965318	0.001659832	0.035135562
CASP8AP2	-0.970870247	0.001663728	0.035155817
APOBR	-0.543868254	0.001683957	0.035495745
GLRX3	0.621150414	0.001690601	0.035495745
ACTN2	3.824217326	0.001693101	0.035495745
TCTN1	-0.579288174	0.001695016	0.035495745
ZNF222	1.480415725	0.001698475	0.035495745
PNKD	-0.440892092	0.00169882	0.035495745
TRIP10	0.867588978	0.001701559	0.035495745
RHOBTB2	-0.716008946	0.00170808	0.035495745
KNOP1	-0.973387051	0.001712175	0.035495745
MRPS36	0.390249567	0.001714933	0.035495745
ECPAS	0.430422983	0.001716691	0.035495745
ALDH1B1	-1.553464363	0.001721582	0.035495745
STPG1	1.059646133	0.001722667	0.035495745
PLRG1	0.578590621	0.0017234	0.035495745
SGSM1	3.93546344	0.001724798	0.035495745
HIRIP3	-0.614510398	0.001736397	0.035495745
GALNTL6	3.400808562	0.001737384	0.035495745
PNLIPRP3	3.498846931	0.00174233	0.035495745
RCHY1	0.658359906	0.001742802	0.035495745
HBP1	0.757997426	0.001742844	0.035495745
SMIM3	0.860086129	0.001744189	0.035495745
ARID3B	1.220184247	0.001745109	0.035495745
PYCR3	-1.273572226	0.001749918	0.035533139
TBC1D32	-0.758420676	0.001761742	0.035593758
RASA1	-0.786576867	0.001761978	0.035593758

RAB3IP	0.487612837	0.001767322	0.035593758
DCAF7	-0.289445632	0.001769214	0.035593758
ZNF267	1.044112538	0.001770544	0.035593758
MMADHC	0.371530741	0.001772935	0.035593758
SPRY2	-1.230262653	0.001773736	0.035593758
CACFD1	0.648680701	0.001792824	0.03579861
LAIR1	-0.945321108	0.001794586	0.03579861
GTF2B	0.956378162	0.001797302	0.03579861
UBE2K	1.207677518	0.001798309	0.03579861
VDR	1.149103282	0.001808268	0.03579861
FCRL6	-1.374020366	0.001811495	0.03579861
PSMB4	1.15901815	0.001814412	0.03579861
BTF3	0.442364255	0.001816343	0.03579861
AHI1	0.970730096	0.001818734	0.03579861
PLEKHH1	0.594566062	0.001820788	0.03579861
GLG1	-0.236252187	0.001825691	0.03579861
SLCO4A1	1.062218355	0.001826009	0.03579861
BRPF1	-0.421367418	0.001829933	0.03579861
WDR3	-0.656278856	0.001831006	0.03579861
LRP12	0.825817876	0.001831017	0.03579861
SELENOK	1.208936248	0.001834244	0.03579861
PTGS2	2.961235057	0.001834828	0.03579861
ASB2	0.784325094	0.001839402	0.035829403
FN3KRP	-0.911326417	0.001843595	0.035852675
SPINK1	3.422911235	0.001847021	0.035860996
SPAG8	-1.763843964	0.001851633	0.035892266
ERO1B	0.78651043	0.001857887	0.035905674
EPC1	0.586867035	0.001864808	0.035905674
TMEM45B	0.822475155	0.001868212	0.035905674
SLC19A2	1.386598448	0.00187094	0.035905674
TARS1	0.50335308	0.001873802	0.035905674
H4C3	-1.019014338	0.001873896	0.035905674
TRIAP1	-0.689599841	0.001875238	0.035905674
ANKUB1	0.378520887	0.001876342	0.035905674
ISCA1	0.794122039	0.001883614	0.035963834
EMC2	0.333917274	0.001886992	0.035963834
SNX16	1.17732922	0.001890459	0.035963834
SLC39A8	-0.842345971	0.001891409	0.035963834
HMGXB3	0.326307537	0.001897527	0.036022898
ZNF577	-0.740664413	0.001907901	0.036043259
CCDC117	1.1412498	0.001908744	0.036043259
SETD1B	-0.373776482	0.001910113	0.036043259
NFE2L2	0.663904657	0.001913556	0.036043259
ZDHHC12	-0.500713188	0.001913668	0.036043259
SUGP2	-0.360726298	0.001927039	0.036238034
UBC	0.989094096	0.00193886	0.036347984
TTF2	-0.574042263	0.001940098	0.036347984
UHRF1BP1L	0.920962938	0.001944895	0.036347984
ARRDC1	-0.920995688	0.001945043	0.036347984
ANKRD1	5.795650073	0.00195	0.036353268
GSTP1	0.303624177	0.001951405	0.036353268
MRM1	-1.046419063	0.001967809	0.036601866
NLRX1	-1.509794474	0.001971671	0.036615458
ADGRA2	1.654132265	0.00197861	0.036615458
GIPR	3.9761842	0.00198172	0.036615458
RGS19	-0.996668862	0.001983307	0.036615458
PANX2	1.716171113	0.001984042	0.036615458
PSMB5	0.882011074	0.001989463	0.036615458
TATDN1	0.37500427	0.001989971	0.036615458
LRCH4	-0.558293202	0.001998385	0.036705946
HSPA9	1.095105811	0.002001026	0.036705946
SPTY2D1OS	1.183705948	0.002010857	0.036798806
AP3M2	-0.675534208	0.002012242	0.036798806
LBHD1	-0.651297273	0.002028765	0.036900946
KBTBD8	2.014494735	0.00204363	0.036900946
CKAP2	-0.77231303	0.002043711	0.036900946
FCHO1	-0.972946121	0.002044836	0.036900946
TEX10	0.973023699	0.002047218	0.036900946
MRPL47	0.42621696	0.002050189	0.036900946
OST4	0.424095035	0.002051737	0.036900946
GEM	1.393672653	0.002051969	0.036900946
UNG	-1.041555796	0.002052396	0.036900946
HSPH1	2.506582722	0.002054031	0.036900946
DNAJB12	0.591084076	0.002056452	0.036900946
IFI16	-0.912933127	0.002056491	0.036900946
TCEANC2	-0.540640272	0.002063211	0.036900946
AKIP1	-0.533972154	0.002069576	0.036900946
VMAC	-1.464706629	0.002070854	0.036900946
KBTBD7	-1.134748945	0.002071568	0.036900946
DUSP1	2.016651834	0.002072535	0.036900946
NTAQ1	-0.367049147	0.002073364	0.036900946
MAPK6	0.910721542	0.002082919	0.037015915
PDCD11	-0.461152564	0.002095862	0.037190667
IFT74	0.783375035	0.002110707	0.037366262
MAP1LC3B	1.065670757	0.002112006	0.037366262
TRANK1	-1.559708515	0.002118606	0.03739567
CHCHD2	0.497259189	0.002124233	0.03739567
SHOX2	-2.00033592	0.002125076	0.03739567
TTC32	0.630089491	0.002127638	0.03739567
ARID1A	-0.74165044	0.002129302	0.03739567
SPEF2	-1.377214814	0.002135497	0.037408803
ZEB2	-0.994309032	0.002138847	0.037408803
ELP4	-0.443036405	0.002144726	0.037408803
ZSCAN5A	1.078652529	0.002146021	0.037408803
SMG5	0.531732858	0.002147535	0.037408803
SLC45A4	1.751080921	0.002148817	0.037408803
ANK1	1.591547327	0.002154393	0.037411156
RBM48	0.803358462	0.002155208	0.037411156
CPEB4	1.129473321	0.002172505	0.037641204

ZNF862	-0.691812298	0.002174755	0.037641204
BNIP3L	0.396312472	0.002180869	0.037692475
HHEX	-1.457514276	0.002193025	0.037811239
ZNF195	0.579281192	0.002196489	0.037811239
FAM219A	1.679603372	0.00219813	0.037811239
MIEN1	0.509393289	0.002200386	0.037811239
ELOC	0.910033659	0.0022093	0.037909945
TPGS2	0.268366955	0.00222031	0.038033334
PRMT7	-0.55840352	0.002222851	0.038033334
GADD45A	2.183109397	0.002227724	0.038062255
PIGQ	-0.564631068	0.002241217	0.038238172
MMP10	4.267518935	0.002263165	0.038549219
PLCB1	1.193189272	0.002266405	0.038549219
PPP1R11	0.418795761	0.002271653	0.038549219
CEP20	0.663216895	0.002274413	0.038549219
DIPK1A	1.05391634	0.002279582	0.038549219
PAQR8	-2.129573578	0.002282883	0.038549219
ZNF365	2.940824148	0.002286701	0.038549219
HSPE1	2.019196023	0.002288287	0.038549219
MBOAT1	-1.508248003	0.002296423	0.038549219
TSPAN14	-0.604471618	0.002303582	0.038549219
VNN3	1.555321601	0.002310003	0.038549219
DNAJC2	1.01340121	0.002310986	0.038549219
PAM16	-1.460533635	0.002315217	0.038549219
KY	2.430690456	0.002321231	0.038549219
FXR1	1.189314618	0.002324702	0.038549219
PSMA1	0.752414174	0.002327197	0.038549219
NDUFB4	0.442207824	0.002331358	0.038549219
LYRM1	0.431783417	0.002333809	0.038549219
TNFRSF10C	-1.229523943	0.002334992	0.038549219
FCHO2	-0.887248828	0.002342189	0.038549219
BCL2L13	0.442688269	0.002343209	0.038549219
GIMAP1	-2.085513912	0.002343535	0.038549219
ARHGEF10L	-1.276314669	0.002343764	0.038549219
NAPEPLD	-0.768093536	0.002344402	0.038549219
TMEM43	-0.422834735	0.002344548	0.038549219
YLPM1	-0.478607045	0.002347888	0.038549219
GABARAPL1	0.998886977	0.002350879	0.038549219
TENT5C	2.553809545	0.00235352	0.038549219
CARD14	-0.578753293	0.002353533	0.038549219
TOX4	0.600594407	0.002357293	0.038549219
PSMG4	-0.940168532	0.002359367	0.038549219
PDCD6IP	0.506554666	0.002365102	0.038568
ARF4	0.761481132	0.002366966	0.038568
KLF7	-0.880073237	0.002370792	0.038570736
SUPT7L	0.561181357	0.002373584	0.038570736
SAP18	0.453727248	0.002379255	0.038610429
TCTA	0.447586799	0.002385209	0.038644218
TPI1	0.287581684	0.002392777	0.038644218
SLC9B1	0.433160644	0.002396582	0.038644218
UBTF	-1.339978704	0.002403872	0.038644218
PSTPIP1	-0.754467041	0.002409856	0.038644218
MEF2C	-1.471614222	0.002412979	0.038644218
RCCD1	-0.922806615	0.002414236	0.038644218
OSBPL3	-0.557541386	0.002415181	0.038644218
GPR65	-1.533431466	0.002416915	0.038644218
ZNF654	1.357262069	0.002417005	0.038644218
SNX3	0.455919622	0.002419619	0.038644218
TCTN3	-0.627378827	0.00242011	0.038644218
WDR35	-0.721548145	0.002425708	0.038666294
SNX17	-0.430697261	0.002431136	0.038666294
ACOT13	0.309575283	0.002431192	0.038666294
PSMD6	1.129795439	0.002443172	0.038805225
CCNK	1.097325701	0.002447304	0.03880609
TET2	0.605090518	0.002449716	0.03880609
GATD3B	-0.707889429	0.002458749	0.038897676
BAG3	3.31415199	0.002465011	0.038945224
EFCAB14	-0.353937667	0.002468514	0.038949119
UBE2D2	0.454013252	0.002475791	0.038961337
KLHL28	0.314885773	0.002479108	0.038961337
ALKBH4	-0.886136471	0.002481418	0.038961337
UBR2	0.520544725	0.002482319	0.038961337
IL1B	2.741882692	0.002491242	0.038977802
PPP3CC	0.737417829	0.002493347	0.038977802
DNMT1	-0.476552116	0.002496356	0.038977802
TMEM167B	0.664012083	0.002499196	0.038977802
TTC30A	-1.233222066	0.002504726	0.038977802
IL36G	1.680468996	0.002506378	0.038977802
PRDX2	0.409033772	0.002506904	0.038977802
NR3C1	0.733514229	0.00250944	0.038977802
ERLIN1	-0.632399552	0.002517244	0.039048301
ZNF253	-0.660402673	0.002537642	0.039213968
ATP6V0D2	1.067237679	0.002537725	0.039213968
CEBPG	0.479097046	0.00253776	0.039213968
GTF2H1	0.590380824	0.002546316	0.0392392
SCYL2	0.7600182	0.002549161	0.0392392
UFD1	1.428774242	0.002549236	0.0392392
LIN54	1.784710806	0.002555067	0.039278408
SLAIN2	0.524459883	0.002561572	0.039318087
HAUS5	-0.780515256	0.002568328	0.039318087
RABGGTB	0.884546452	0.002568946	0.039318087
GPR39	-1.297871625	0.002570798	0.039318087
INSIG1	0.899300549	0.002582402	0.039345855
H3-3B	0.581264489	0.002591349	0.039345855
RECQL4	-0.94771534	0.002593216	0.039345855
KLHL21	1.87488757	0.002599589	0.039345855
GHITM	0.519181822	0.00260377	0.039345855
MARCHF6	0.467798924	0.002605662	0.039345855
F2RL2	3.114551186	0.002607251	0.039345855

CDC37	0.707434119	0.002609821	0.039345855
TTC39B	0.815330037	0.002612349	0.039345855
ZNF786	-0.727817671	0.002612399	0.039345855
SNX5	-0.621026504	0.002613639	0.039345855
GCSAML	2.608208742	0.002614152	0.039345855
CNIH1	0.337561906	0.002615381	0.039345855
TCTN2	-0.619794664	0.002631358	0.039409502
EIF2A	0.824406779	0.002633705	0.039409502
DPH3	0.551866856	0.002634697	0.039409502
TPRG1	-0.695844741	0.002638916	0.039409502
PSMD2	0.940730188	0.002641449	0.039409502
ASTE1	-0.365284846	0.002642908	0.039409502
SMAP1	0.701972157	0.002644262	0.039409502
MGST1	0.544874359	0.002647631	0.039409502
NCSTN	-0.284606511	0.002651836	0.039409502
P2RX7	-1.238929092	0.002655705	0.039409502
KLF3	-1.064743914	0.002655858	0.039409502
STAT6	-0.558663345	0.002663825	0.039478738
PRRT3	1.26325582	0.002672208	0.039553973
NR2C2AP	-0.164714623	0.002678638	0.039574463
FAM160B2	-0.723843362	0.00268021	0.039574463
TIPIN	-0.50434581	0.002693335	0.039669907
RAB2A	0.427253812	0.002696227	0.039669907
S100Z	1.013244554	0.002698498	0.039669907
DOCK8	-0.543876038	0.002701087	0.039669907
SYF2	0.606449042	0.002703934	0.039669907
PSMA7	0.582208807	0.002709578	0.039669907
ADPRHL1	2.77933416	0.002711791	0.039669907
RHEB	0.808354998	0.002713209	0.039669907
SLC17A5	0.564855149	0.002739844	0.039938957
PLEKHA2	-1.096590034	0.002741042	0.039938957
CCT8	0.342946336	0.00274507	0.039938957
NOP16	-0.319609534	0.002745989	0.039938957
KANSL2	-0.65983042	0.002748308	0.039938957
MBLAC2	-2.092655694	0.002761419	0.040017554
PCSK5	1.431912804	0.002767323	0.040017554
DAPP1	-1.616721412	0.002772174	0.040017554
C1orf43	0.442590676	0.002772339	0.040017554
CCDC77	1.20564553	0.002774057	0.040017554
TIFAB	-3.344963067	0.002782437	0.040017554
PCDH9	2.517111673	0.002785901	0.040017554
ENO2	0.963391409	0.00278642	0.040017554
TTI1	-0.365395155	0.002786773	0.040017554
GPBP1	0.753826533	0.002787905	0.040017554
STRN	0.5114612	0.002790522	0.040017554
HADHB	0.27164472	0.002797561	0.040070451
HACD2	-0.370563806	0.002812071	0.040179918
TENT5A	-0.609061801	0.002816709	0.040179918
STARD13	-1.179492322	0.002818666	0.040179918
BUD31	0.554964945	0.002822675	0.040179918
VCP	0.905752814	0.002824861	0.040179918
MRPL27	0.657245592	0.00282536	0.040179918
POR	0.867472696	0.002830714	0.040208236
DYRK3	1.198121875	0.002845933	0.040257049
MARS2	-0.909709287	0.00284622	0.040257049
FGD2	-1.397891319	0.002847505	0.040257049
EIF4A3	0.844845998	0.002848261	0.040257049
CSGALNACT2	0.598514609	0.00285098	0.040257049
RARG	1.446833812	0.002860399	0.040258636
PPP6R3	0.620416608	0.00286132	0.040258636
NPPC	1.608861829	0.002863208	0.040258636
PYCARD	-1.21787603	0.002870546	0.040258636
TRAF3IP3	-1.192853212	0.002871034	0.040258636
CUL7	-0.708989715	0.002871289	0.040258636
RABGEF1	0.838264706	0.002875019	0.040263738
MAD2L1BP	0.646536423	0.002886931	0.040366715
PANK1	-0.726832871	0.002889676	0.040366715
GPRC5C	1.820634268	0.002892832	0.040366715
PYCR2	-0.613989241	0.002895873	0.040366715
DHDH	0.969094055	0.002901042	0.040391696
ADAP2	-0.910840797	0.002913633	0.040464563
SDHAF2	0.937554649	0.00291477	0.040464563
CCDC134	0.972091612	0.002916426	0.040464563
DNAJC5B	1.37671208	0.002925358	0.040541457
ATF7IP2	2.417270996	0.002939095	0.040659843
PRKCH	0.920758247	0.002940699	0.040659843
PTK2B	-0.680981669	0.002947418	0.040668445
ZNF677	-0.838405449	0.002948122	0.040668445
ENTHD1	1.837313726	0.002960974	0.040751771
TEP1	-0.709311877	0.00296402	0.040751771
CLEC2B	0.512865901	0.002964385	0.040751771
SF3B6	0.337016613	0.002969219	0.040771367
MAML2	-0.977997113	0.002986879	0.040877298
GIMAP6	-1.54335192	0.002989371	0.040877298
INPP5D	-0.856848797	0.002989801	0.040877298
HAS3	-0.430606009	0.002990959	0.040877298
RNF185	0.563079463	0.002996565	0.040877298
RBM7	0.699995839	0.002999853	0.040877298
UBE2H	1.111425145	0.003000859	0.040877298
ZBTB43	1.207011644	0.003009292	0.040891044
GPX4	0.311157844	0.003011156	0.040891044
CCDC200	-0.747574278	0.003012125	0.040891044
DGKG	2.068199929	0.003016715	0.040906926
TUBD1	0.509536924	0.003020448	0.040911166
STUM	-1.952642075	0.003028642	0.040920941
ADD1	-0.610018312	0.00303139	0.040920941
OLFML2B	-1.734671636	0.003031434	0.040920941
ACBD3	0.77187634	0.0030442	0.041035516
CLEC12A	0.663066811	0.003050364	0.041035516

ELAC1	-0.610865038	0.003058941	0.041035516
GPRIN3	-0.897282954	0.003060652	0.041035516
RAG2	0.74830933	0.003060849	0.041035516
CNST	0.903330011	0.003063586	0.041035516
BABAM2	0.393740878	0.003064835	0.041035516
SUGT1	0.442700781	0.003070561	0.041035516
GARS1	0.861422996	0.003070802	0.041035516
TSEN2	-0.908855371	0.00307661	0.041067254
LPCAT4	-1.393753257	0.003083878	0.041118374
KANSL1L	-0.282352003	0.003097107	0.041169269
CNIH3	-1.294173533	0.003097633	0.041169269
RC3H1	0.885747163	0.003100956	0.041169269
NPM1	0.779556644	0.003112101	0.041169269
FLVCR1	-0.5321587	0.003114061	0.041169269
PLEKHG4	-0.8340592	0.003114093	0.041169269
MCRIP2	0.368704632	0.00311714	0.041169269
EGFL7	0.700765124	0.003120565	0.041169269
LARP4	1.012893084	0.003121616	0.041169269
IRAG2	-0.898010685	0.003122683	0.041169269
HSD17B1	-1.301579148	0.003127026	0.041169269
SCRN2	-0.681698824	0.00312916	0.041169269
LEKR1	1.096761009	0.003132444	0.041169269
RAB5IF	0.832462881	0.003140734	0.041232901
MED13	1.098693864	0.00315122	0.041285801
GSR	0.992607338	0.003151667	0.041285801
BRAT1	-0.695718114	0.003155373	0.041289127
SMU1	0.322152518	0.003174849	0.041498567
VAPA	0.369977747	0.003178491	0.041500825
PLCG2	-0.18392019	0.003195347	0.04166137
CAB39L	0.813413014	0.003200076	0.04166137
SCLT1	-0.750676499	0.003205327	0.04166137
RSAD2	1.3586277	0.003205439	0.04166137
BTBD19	0.74977837	0.003215821	0.04166137
ROM1	0.494816641	0.003218395	0.04166137
MRPS6	0.637122892	0.003221422	0.04166137
PGD	0.379665044	0.00322166	0.04166137
HOOK3	0.534135807	0.003222138	0.04166137
SLU7	0.834072791	0.00323222	0.04170773
ITGB3	0.821042764	0.003233376	0.04170773
DOP1A	0.587112584	0.003238304	0.04170773
PSMB2	0.887336455	0.003239672	0.04170773
IMPA1	0.52694861	0.003256262	0.041876231
PAK1	-0.894001576	0.003260726	0.041888592
TACC2	1.507084974	0.003269356	0.041953884
PUS7	-0.580013802	0.003272824	0.041953884
PNPLA3	-1.568564446	0.003282419	0.042031829
TRIM39	0.369787222	0.003291163	0.042098729
C2orf42	0.757755716	0.003296971	0.042113903
GTF2F2	0.587333933	0.003299426	0.042113903
AMZ2	0.168927352	0.003305884	0.042113903
KLHL8	-0.409144528	0.003308475	0.042113903
SENP5	0.661754151	0.003309956	0.042113903
CRYBG1	-0.97461261	0.003316889	0.04215727
FBXO4	-0.961836068	0.003329173	0.042164999
PGM3	0.936611087	0.003330234	0.042164999
RSRC2	1.140330192	0.003333656	0.042164999
ARL6IP1	0.380605675	0.003334427	0.042164999
RHNO1	-0.636720882	0.003335124	0.042164999
CSF2	4.090186672	0.00336273	0.042352205
PCBD2	-0.386907444	0.003363171	0.042352205
C19orf25	0.471669492	0.003364244	0.042352205
MAPKBP1	-0.701664995	0.00336819	0.042352205
RPL41	0.34760667	0.003372101	0.042352205
GPATCH3	0.989505631	0.003372816	0.042352205
CD109	1.175881746	0.003375256	0.042352205
NEDD4L	1.256856575	0.003378261	0.042352205
EIF3E	0.484962644	0.003383187	0.042369548
CCDC80	1.338591482	0.003393467	0.042453838
DDX43	1.848129851	0.00339978	0.042475279
BTN3A3	-0.795290364	0.003402602	0.042475279
SECISBP2	0.69026206	0.003405835	0.042475279
SNAI1	3.541684296	0.003417786	0.042549423
POLM	-0.934666475	0.003423229	0.042549423
ACTL6A	-0.612239458	0.003424562	0.042549423
KCNJ2	-1.478355415	0.003426011	0.042549423
WDR81	-0.840582258	0.003438666	0.042662284
PSMC1	1.208064151	0.003450168	0.042760636
MCM4	-1.284151297	0.00346888	0.042933146
EXOC8	0.983233931	0.003480429	0.042933146
C17orf67	1.741223702	0.003482731	0.042933146
CHRNG	-0.836626671	0.003486348	0.042933146
SH3BP4	2.253239068	0.003489156	0.042933146
JSRP1	0.312157172	0.003490398	0.042933146
NRDE2	1.142527173	0.003490722	0.042933146
METAP2	0.52742482	0.003492805	0.042933146
ZFP37	2.053547456	0.003497486	0.042938617
PSMA2	0.933381348	0.003500431	0.042938617
NUDT19	-0.559553933	0.003511869	0.0429839
RPS3A	0.417106739	0.003514235	0.0429839
DOLK	-0.878048048	0.003518581	0.0429839
UCK2	-0.720181816	0.003526083	0.0429839
PFDN5	0.274147605	0.003527186	0.0429839
NPAS2	2.964955121	0.003527804	0.0429839
UQCRC2	0.296663088	0.00352928	0.0429839
METTL3	0.518816928	0.0035438	0.04311684
FAM214A	-0.536159206	0.003549667	0.04312168
KBTBD6	-0.972563771	0.003552759	0.04312168
SNRPB2	0.587724492	0.003555015	0.04312168
SPDYE2	-0.961655925	0.0035661	0.04321231

ZNF585A	-0.658524823	0.003572036	0.04324044
CLEC17A	4.002423948	0.003579959	0.043292524
CBX6	-0.952252815	0.003597015	0.043454849
CNTLN	-0.780434881	0.003616247	0.043610357
SETMAR	0.689577837	0.003617876	0.043610357
JMJD6	1.058305697	0.003620826	0.043610357
HIGD1A	0.31100234	0.003642442	0.04382657
CNNM2	0.443029349	0.003654793	0.043895222
ABHD17B	1.103766121	0.003658196	0.043895222
NKX3-1	2.370096223	0.003659159	0.043895222
EIF2S2	0.347162095	0.003664284	0.043912662
RPS27	0.50707233	0.003676765	0.04400011
GADD45B	2.210146909	0.003678939	0.04400011
CXCL8	3.997218125	0.003682779	0.044002039
PSMD11	1.161876698	0.003695555	0.044066886
CD274	1.356309258	0.003703917	0.044066886
CCDC78	-0.783115266	0.003706601	0.044066886
KLHDC10	0.573280974	0.003715621	0.044066886
MARK3	0.481174498	0.003716169	0.044066886
FKBP5	-0.620194117	0.003728669	0.044066886
EEF2KMT	-0.558860852	0.003729789	0.044066886
H4C9	1.239752326	0.003734076	0.044066886
AK8	1.058718047	0.003735614	0.044066886
SPDYE2B	-1.734455227	0.003735972	0.044066886
NR4A2	2.914995611	0.003737453	0.044066886
HECW2	2.530101968	0.003738082	0.044066886
MPZL1	0.411806382	0.003738489	0.044066886
WDFY2	-0.640751212	0.00373979	0.044066886
CMKLR1	-2.134708993	0.003747834	0.044118198
GNA13	0.448252536	0.003757146	0.044138991
PSMC5	0.776225156	0.003757435	0.044138991
CCDC59	0.698185688	0.003763556	0.044138991
RNF144A	0.921316714	0.003765859	0.044138991
ATP1B3	0.538176273	0.003768053	0.044138991
KCNE1	1.277056701	0.003775027	0.044151433
ZBTB9	-0.725658465	0.003776868	0.044151433
LBR	-0.582785636	0.00378019	0.044151433
MSH2	-1.191150421	0.003792438	0.044239033
LGALS3	0.283226686	0.003800511	0.044239033
SLA2	0.605096298	0.003805633	0.044239033
RBL1	-0.916092875	0.003807048	0.044239033
STX3	0.485692559	0.003810866	0.044239033
RNF26	-0.978191665	0.003814303	0.044239033
MRPL44	0.551039048	0.003814904	0.044239033
C9orf64	-0.74560678	0.003817542	0.044239033
C11orf54	0.402168468	0.003823794	0.044239033
ADGRF3	1.310145653	0.003824679	0.044239033
FAM53C	0.498587889	0.003837086	0.04433966
ATP6V0B	0.353598787	0.003848618	0.044401044
TMEM38B	0.631712628	0.00385656	0.044401044
ZNF33B	-1.241345293	0.003856898	0.044401044
STARD5	-0.379564599	0.00385995	0.044401044
KIAA0319L	-0.292394796	0.00386096	0.044401044
HAUS8	1.203804469	0.003868524	0.044445295
IFRD1	1.02338063	0.003880676	0.044542117
TAOK2	-0.434955695	0.003887677	0.044579695
CHAC1	1.52419326	0.003893839	0.044607585
MCM3	-1.53587083	0.003914083	0.044796586
UBE2V2	0.23156797	0.003925733	0.044801386
ZDHHC24	-0.278247506	0.003928748	0.044801386
B3GNT7	-1.204710936	0.003931957	0.044801386
SH3TC2	4.136542812	0.003933739	0.044801386
ADCY7	-0.931446571	0.003936026	0.044801386
PDP1	-0.334227947	0.003936978	0.044801386
CEBPZOS	0.577746387	0.003954407	0.044956943
CTDSP2	-1.334032882	0.003960742	0.044960633
ECHDC3	-0.45000749	0.003964627	0.044960633
TAB2	-0.620809798	0.003966009	0.044960633
CREM	1.171249017	0.003974253	0.044969453
TK2	-0.915202709	0.003974307	0.044969453
NLN	-0.44040802	0.003979713	0.044988058
PAXIP1	-0.19458905	0.003985973	0.045016281
LIX1L	-0.885138833	0.003998982	0.045076213
NEK6	-0.609790027	0.00400092	0.045076213
RPL9	0.439075344	0.004002587	0.045076213
ZDHHC17	0.474721562	0.004006608	0.045079049
LIMS2	1.109689273	0.004024143	0.045197737
TRPS1	-1.56746139	0.004024732	0.045197737
FOSL1	2.032382964	0.004033736	0.045197737
IPO9	-0.106966566	0.00403675	0.045197737
ZFP62	-0.912424795	0.004039209	0.045197737
UBA6	0.553907308	0.004041202	0.045197737
LAMA5	1.214313399	0.00404361	0.045197737
UBTD2	0.848643636	0.00408682	0.045628466
ZNF621	-0.512859172	0.004093249	0.045628466
SH2B3	-0.319964005	0.00409982	0.045628466
BACH1	0.594343108	0.004106796	0.045628466
SRGAP2C	-0.629517408	0.004107715	0.045628466
PCMTD2	-0.915343084	0.004109556	0.045628466
BRK1	0.257149116	0.00411129	0.045628466
ACADS	-0.364757074	0.00411799	0.045628466
PDK1	0.719691348	0.004120453	0.045628466
OTULINL	-1.401508029	0.004125232	0.045628466
MT1M	3.384550293	0.00412792	0.045628466
SAP30BP	0.693663449	0.004131639	0.045628466
SCAMP1	0.276995338	0.004131742	0.045628466
B3GALNT2	-0.210619238	0.004141483	0.045655499
TEDC2	-1.004497364	0.004144525	0.045655499
LY6K	2.702119127	0.004145641	0.045655499

TMEM116	0.589137312	0.004153636	0.045701459
CHST10	-0.805146067	0.004170353	0.045751484
CMTM7	-0.556812773	0.004171952	0.045751484
ADRA2B	-1.716601505	0.004174656	0.045751484
STEAP1	-0.926417437	0.004177057	0.045751484
CSK	-1.608999546	0.004179	0.045751484
SMC6	-0.718203183	0.004181135	0.045751484
TRAM2	-1.086109934	0.004200047	0.045846035
ATR	-0.783681027	0.004203704	0.045846035
HADHA	0.208288716	0.004206074	0.045846035
TBC1D4	-0.999600648	0.004209781	0.045846035
DOK3	-0.650692396	0.004211266	0.045846035
ZBTB14	-1.215291092	0.004212775	0.045846035
HSP90AA1	1.742281211	0.004228616	0.045946424
GABRR2	2.488164413	0.004229683	0.045946424
ADRM1	1.179647857	0.004233756	0.045948928
UNKL	0.594461759	0.004243533	0.046003007
DCUN1D1	0.391676438	0.004246431	0.046003007
ZFAND6	0.591362145	0.004257668	0.04606265
ARMC8	-0.602148467	0.00425964	0.04606265
UBA52	0.514611913	0.004265315	0.046068585
ARC	5.359721207	0.004268251	0.046068585
OAZ1	0.294652998	0.004271744	0.046068585
RMC1	0.500211804	0.004279363	0.046074189
TXNRD3	-0.76519884	0.004279969	0.046074189
MRPL32	0.538896023	0.004289487	0.046077206
B3GNT5	1.372294408	0.004289537	0.046077206
LAPTM4B	1.268065177	0.004292164	0.046077206
ZNF268	-0.537321244	0.004295659	0.046077206
FAAP100	-0.445160194	0.004302267	0.046106733
AHSA1	1.53549344	0.004314403	0.046150361
RAF1	0.298463163	0.004316827	0.046150361
SRGAP2	-0.769124479	0.004320731	0.046150361
BFSP1	3.395871064	0.004325365	0.046150361
HVCN1	-0.717704061	0.004325632	0.046150361
ZNF561	-0.579808108	0.004336755	0.046221331
PSMD7	0.777409556	0.004340013	0.046221331
HSP90AB1	1.220299484	0.004351135	0.046297419
TIMP3	-1.222408876	0.004354899	0.046297419
TMEM106A	-0.583961647	0.004365271	0.046310491
RAB11FIP1	-0.617773418	0.004365419	0.046310491
CD1D	-1.769293372	0.004367745	0.046310491
TMEM217	0.991691812	0.004390226	0.046464491
FAM186B	2.204559161	0.004390472	0.046464491
MYD88	-1.048749695	0.004397683	0.046464491
ZNF554	-1.658756629	0.00439781	0.046464491
TENT5B	4.014657489	0.00441254	0.046570778
TMEM161B	0.495562732	0.004416245	0.046570778
ZNF529	0.711125461	0.004424708	0.046570778
ARRB1	-1.150933592	0.004428144	0.046570778
TBC1D22A	0.257988738	0.004428465	0.046570778
ETV5	-1.156782639	0.004431233	0.046570778
EFHC1	-0.849824348	0.004445776	0.046600701
TBC1D23	0.43460158	0.004448367	0.046600701
RNMT	0.474108957	0.004449886	0.046600701
CSNK2A1	0.587704179	0.004451936	0.046600701
IDH3B	0.500368412	0.004453562	0.046600701
KIAA0232	0.711242153	0.004460836	0.046636017
CGGBP1	0.370814916	0.004475343	0.046746816
TNRC6B	-0.541151382	0.004480038	0.046755018
GPR108	0.346733827	0.004487023	0.04678709
H1-2	1.233115637	0.004497978	0.046860469
EDEM2	-0.310670787	0.004506061	0.046874597
CXCL2	1.737666135	0.004510657	0.046874597
ZNF213	-0.356186871	0.004512546	0.046874597
PXMP4	-1.19251902	0.004515011	0.046874597
PIK3R6	-0.640084316	0.004520358	0.046889406
GAPDHS	-1.72813075	0.004532436	0.046938084
HIKESHI	0.383203894	0.004535549	0.046938084
SHPRH	-0.804462752	0.004536825	0.046938084
BLOC1S2	0.637677114	0.004551224	0.047046359
BORCS5	-0.821858595	0.004570787	0.047075184
TMPRSS5	0.688793293	0.004571194	0.047075184
IMPACT	-0.50523389	0.004572009	0.047075184
XBP1	0.798578595	0.004573269	0.047075184
NSD1	-0.807542082	0.004573693	0.047075184
SMARCC2	-0.545215674	0.004586478	0.047117788
DDIT3	1.357340683	0.00459137	0.047117788
DLD	0.468459152	0.004595455	0.047117788
SNX20	-1.093220366	0.004596914	0.047117788
SLC16A5	-0.84453017	0.00459753	0.047117788
EIF3L	0.433694621	0.004608035	0.047185014
EPS15L1	0.754540339	0.004615904	0.047225155
PLCE1	-0.865484559	0.004625174	0.047244357
ZMAT2	0.485892332	0.004625681	0.047244357
HSPA13	1.040044973	0.00463834	0.047282169
CTNNBL1	0.559423449	0.004642886	0.047282169
STK40	0.670537257	0.004645583	0.047282169
SMOX	0.974210422	0.004647376	0.047282169
TAF1C	-0.402128119	0.004649711	0.047282169
HSPA12A	2.086831575	0.004653176	0.047282169
FAU	0.309831622	0.004657056	0.047282169
GPAM	-0.703510356	0.004669942	0.047309983
H2BC5	2.035370405	0.004679426	0.047309983
ZBTB2	1.416760923	0.004683842	0.047309983
ZNF483	2.243921316	0.004684626	0.047309983
CALU	0.629578801	0.004691344	0.047309983
TKT	0.250170881	0.004692666	0.047309983
FNIP1	0.774439599	0.004694628	0.047309983

SNX10	0.37306128	0.004698743	0.047309983
APEX1	-0.885357267	0.004699755	0.047309983
LILRB2	-1.098070863	0.004701242	0.047309983
PPP1R12A	0.512705545	0.004703308	0.047309983
TP53BP1	-0.310825933	0.004707954	0.047316915
PPP1R16A	-0.852163676	0.004715085	0.047348793
TAF2	0.454624226	0.004725294	0.047411509
YOD1	0.806011161	0.004730938	0.047422832
QSER1	-0.840857078	0.004738261	0.047422832
EIF3I	0.666640932	0.004741023	0.047422832
CLN6	-0.683578057	0.004742283	0.047422832
GFPT1	0.58294013	0.004754611	0.047459219
GOLIM4	-0.493315492	0.004756609	0.047459219
ACAD10	-0.673091378	0.004758584	0.047459219
ENDOV	0.973255981	0.004761795	0.047459219
TPPP	1.289420549	0.00478344	0.047536603
POPDC3	2.880385694	0.004784387	0.047536603
ZNF189	0.651754926	0.004785209	0.047536603
ZC3H7B	-0.672680145	0.004785457	0.047536603
TPD52	0.848317809	0.004795457	0.047592662
ATXN7L1	-0.733627085	0.004799059	0.047592662
PDK2	-1.042971558	0.00480629	0.047624874
MAP1A	1.78978688	0.004820468	0.047725821
CD2AP	-0.453104541	0.004824675	0.047727968
TMEM123	0.336020003	0.004832277	0.047763661
TRIM38	0.445378915	0.004856988	0.047862559
KDM3B	-0.712535567	0.004857315	0.047862559
SLC38A10	-0.404301633	0.004858396	0.047862559
EIF2AK3	1.281676252	0.004863099	0.047862559
PPIH	0.487016199	0.004863868	0.047862559
SIMC1	-0.575324247	0.004866294	0.047862559
GNAL	-0.350803254	0.004873666	0.047895681
VMP1	0.557710971	0.004887309	0.047990321
EFCAB2	0.385675607	0.004894912	0.048008753
HMG20A	-0.573937275	0.004897214	0.048008753
PTAFR	-1.294637024	0.004902392	0.048020151
NRP2	-0.518680775	0.00491336	0.048057185
MAP4K5	0.604970341	0.004914209	0.048057185
RABGAP1L	0.673061691	0.004922476	0.048098705
CTNND2	1.456638213	0.004932259	0.048126255
CCT3	0.537945248	0.00493901	0.048126255
FICD	0.651115651	0.004939798	0.048126255
SUOX	-1.131921262	0.004941391	0.048126255
RPL19	0.284499711	0.004957427	0.048212766
H2BC12	1.130893741	0.004958945	0.048212766
EXTL3	-0.203186772	0.004967763	0.048212766
DUSP7	-1.460381108	0.00496828	0.048212766
MED22	-0.77000327	0.00497043	0.048212766
FAM32A	0.652012084	0.004978239	0.048229142
PSMB1	0.613031992	0.004980506	0.048229142
RPL21	0.363653576	0.004984216	0.048229142
GINS1	-1.04219172	0.004994515	0.048256555
GTPBP3	-0.8937182	0.004995873	0.048256555
FASTKD5	0.966802346	0.005002404	0.048256555
MRPS17	-0.789909004	0.005008451	0.048256555
DSC2	-0.657193631	0.005008871	0.048256555
HEATR5B	-0.577890786	0.005014506	0.048256555
ODR4	0.497922973	0.005015292	0.048256555
CNOT4	0.80939751	0.005021978	0.048280401
COX6C	0.315504258	0.005025948	0.048280401
PLCD1	1.108158225	0.005029881	0.048280401
GLS	0.843445515	0.005036263	0.048285166
BTBD7	0.683452778	0.005038452	0.048285166
ABI1	0.33030557	0.00505606	0.048415115
PRKAG2	0.403770654	0.005062064	0.048433827
TGFBI	-1.014131615	0.005074082	0.048510011
IFNLR1	-1.312294589	0.005084754	0.048573205
UQCC3	-0.691790844	0.005100205	0.048598522
RIOK3	0.898485653	0.005109301	0.048598522
DZIP1L	-1.421906553	0.005110391	0.048598522
CNBP	0.518703789	0.005111275	0.048598522
SLC27A1	-0.585322918	0.005113048	0.048598522
LRRCC1	-0.693724441	0.005114367	0.048598522
LRRC25	-1.390028041	0.005119995	0.048598522
UBN1	0.491714037	0.005120134	0.048598522
CWC25	1.165170745	0.005123975	0.048598522
IKBIP	-0.545877796	0.005128886	0.048606561
NANS	0.692528973	0.005146566	0.048735492
PSMB8	-0.419841128	0.005161201	0.048835416
TBK1	0.561843822	0.00519031	0.049072024
ASPH	0.701062961	0.005203492	0.049078894
SLF2	-0.822710103	0.005205528	0.049078894
UBE2J2	0.368519677	0.005212601	0.049078894
RREB1	-0.755994764	0.005213068	0.049078894
TRMT112	0.434451185	0.005213112	0.049078894
SH3BP5	0.727775472	0.005216762	0.049078894
PSMB3	0.534872902	0.005224843	0.049078894
SGCG	0.683042877	0.00522928	0.049078894
PIK3C3	0.42352116	0.00523711	0.049078894
MBOAT4	3.694074507	0.005238353	0.049078894
PIK3AP1	-0.259595694	0.005239444	0.049078894
LIMD2	-1.008726329	0.00524028	0.049078894
DNAAF1	-0.545422897	0.0052492	0.049123973
CABP7	0.488351939	0.005260371	0.049190021
DNAJC8	0.42238389	0.005267151	0.049214938
SAMD14	-0.963297916	0.005281324	0.049308851
CAPS	-0.718400447	0.005296823	0.049349207
UQCRC1	0.203273416	0.005300025	0.049349207
PTGR1	0.913324117	0.005300724	0.049349207

CLDN14	2.367681814	0.005303358	0.049349207
WDR77	-0.544034042	0.005309623	0.049349207
LRRC45	-1.132574716	0.005311279	0.049349207
DNAJC3	0.661572407	0.00531453	0.049349207
PCYOX1L	-0.971088215	0.005322778	0.04938745
DOP1B	-0.755399462	0.00532712	0.049389424
NAA60	-0.188328762	0.0053321	0.049397299
PSKH1	-0.695229301	0.005344376	0.049472711
RAE1	0.647747134	0.005360847	0.049531567
MAFK	1.194906132	0.005363727	0.049531567
TNFSF14	1.079110273	0.005365287	0.049531567
KLHL42	-1.142237798	0.005369109	0.049531567
TNFRSF1A	-0.50555281	0.005371442	0.049531567
ZNF248	-0.928878223	0.005381844	0.049570206
GPATCH2L	0.671543065	0.005386597	0.049570206
PDCD10	0.471356055	0.005391257	0.049570206
PTRH1	-0.401827392	0.005392211	0.049570206
GPT2	0.61097742	0.005398518	0.04959007
TRAPPC2B	1.012407669	0.005410663	0.049648271
BLCAP	-0.37130009	0.005413156	0.049648271
NOD1	-1.141104151	0.005424789	0.04971684
ZNF783	-0.960654811	0.005434002	0.049763146
PDE6B	-0.744772988	0.005438732	0.049766409
USP14	0.861499653	0.005442681	0.049766409

Table.S6

GSEA Pathways by 12,13-diHOME alone compared to DMSO

ID	Description	Enrichment Score	NES	Unadjusted P	Adjusted P	Core Enrichment Genes
WP2882	Nuclear receptors meta-pathway	0.450576096	1.88872813	3.80E-07	0.00017831	SERPINB2/NQO1/CYP1B1/ABCB4/SLC2A3/GSTM3/IL1B/SCD/TXNRD1/TNF/NFE2L2/SQSTM1/PTGR1/PGD/CPEB4/TNFAIP3/HBEGF/ANGPTL4/SLC7A11/SLC6A6/SLC5A3/BHLHE40/CYP1A1/CPT1A/CDC42EP3/NCOA3/BIRC3/PDGFB/THBD/VDR/JUNB/KEAP1/PRRG4/SLC2A1/PDK4/CCL20/ARL5B/CUL1/NR3C1/MGST1/ANKRD1/ABCB1/ALAS1/SLC2A14/HSP90AB1/CDC37/HSPA1A/MYOF/PTGS2
WP477	Cytoplasmic ribosomal proteins	-0.528454031	-2.040317	5.08E-06	0.00119014	UBA52/RPL19/RPS17/RPL23A/RPS6KB2/RPLP2/RPL27A/RPS14/MRPL19/RPS5/RPS27A/RPL21/RPS6KA2/RPL41/RPS19/RPS15/RPS13/RPS15A/RPL36/RPL17/RPL22/RPS18/RPL38/RPL23/RPS11/RPL10A/RPL32/RPL30/RPL9/RPS12/RPL37/RPS16/RPL34/RPL35A/RPS25/RPL31/RPL37A/RPL5/RPS26/RPL35/RPS27/RPL27/RPS21/RPS29/FAU/RPL11/RPS24/RPS23/RPL29/RPS8
WP2873	Aryl hydrocarbon receptor pathway	0.705141094	2.0861572	2.22E-05	0.0032502	SERPINB2/NQO1/CYP1B1/IL1B/TNF/NFE2L2/CYP1A1/JUNB/MGST1/CDC37/MYOF
WP3932	Focal adhesion: PI3K-Akt-mTOR-signaling pathway	0.409121444	1.72089168	2.77E-05	0.0032502	LPAR2/CDKN1A/THBS1/RAF1/SLC2A3/ATF4/OSMR/ITGB7/PIK3R5/LPAR6/PDGFB/IFNAR1/PIK3CB/ITGAE/PIK3R4/DDIT4/PPP2R5B/CREB5/SLC2A1/EIF4E/ITGAL/PIK3C2A/GNG11/PPP2R1B/COL6A2/ITGB3/VEGFA/IL2RA/PELO/HSP90AB1/ITGA11/PPP2R5D/ATF2/CDC37/LAMA5/TSC1/EIF4E2/GNGT2/PDPK1/PTEN/FOXO3/LPAR3/PPP2CA/RPTOR/NRAS/PDGFRA/RAB10/ITGB8/HSP90AA1/GNG3/COL5A3/PIK3C2B
WP3945	TYROBP causal network in microglia	-0.495344805	-1.7975344	0.000375808	0.02464707	CD37/GAL3ST4/TGFBR1/LOXL3/RNASE6/LYL1/IL18/NRROS/MAF/SLC7A7/ZFP36L2/APBB1IP/PYCARD/ADAP2
WP615	Senescence and autophagy in cancer	0.460762873	1.72639843	0.000397283	0.02464707	SERPINB2/MAP1LC3B/CDKN1A/THBS1/RAF1/IL1B/SQSTM1/IRF5/RB1CC1/ATG13/IL1A/PIK3C3/SERPINE1/IL6ST/GABARAPL1/SMAD3/CXCL8/IRF1/RB1/PTEN/CCL3/IGFBP7/ATG14/SLC39A1/ATG16L1/SH3GLB1/KMT2A/GSK3B/BECN1/RSL1D1/HMGA1/IL6R
WP4493	Cells and molecules involved in local acute inflammatory response	0.800442712	1.95295663	0.000410052	0.02464707	TNF/IL1A/C3/ITGAL/CXCL8/ICAM1
WP3995	Prion disease pathway	-0.597983031	-1.895284	0.000420419	0.02464707	NFKB1/BATF/BCL2/CTCF/MAPK3/RXRA/POU2F2/IRF4/MEF2C/BCL11A
WP2059	Alzheimer's disease	0.378466894	1.59082778	0.000502431	0.02618221	RAF1/IL1B/TNF/ATF4/PSMC1/RB1CC1/ADAM17/PSMD13/ATG13/CASP7/KIF5B/IL1A/IDE/PIK3CB/TRAF2/PIK3C3/PLCB1/PIK3R4/CHUK/KLC2/TUBA4A/PSEN1/PSMD1/GAPDH/DDIT3/CSNK2A1/ITPR3/RTN3/TUBB6/PTGS2/PSMB7/CSNK1A1/CASP3/ADRM1/NRAS/CHPF2/FZD6/ATG2B/WNT11/ATG14/PSMC4/PSMD7/TUBB4A/PSMB2/PSMC6/CTNNB1/ATG101/PPP3CC/NAE1/PSMC3/RYR3/GSK3B/MAPK8/BECN1/PSMB4/PSMD8/ITPR2/PSMD14/PIK3R1/PSMD4/MCU/PPID
WP143	Fatty acid beta-oxidation	0.653135066	1.89054118	0.000568811	0.02650037	CPT1A/HADHB/GPD2/HADHA/ACSS2/TPI1/ACSL3/ACADVL/GCDH/SLC25A20
WP2586	Aryl hydrocarbon receptor Netpath	0.557614428	1.8275226	0.000621544	0.02650037	NQO1/CDKN1A/CYP1B1/RAF1/TNF/NFE2L2/CYP1A1/VEGFA/CDC37/RB1/PTGS2/NRAS/HSP90AA1
WP536	Calcium regulation in cardiac cells	-0.432252346	-1.7103092	0.000680378	0.02659146	ADCY9/GJB2/ADCY7/RGS9/GNB5/PRKCB/GNA11/GRK6/FKBP1A/STIM2/CALM1/ATP1B3/GNAS/PRKACB/PRKCA/GNB4/RYR1/PRKACA/KCNJ5/GNB1/RGS2/CAMK2D/PRKCD/CALM3/RGS3/GNAQ/GNB2/ANXA6/GNAI2/PRKAR1B/YWHAH/RGS18/ATP1B1/SLC8A1/ARRB1/ADRB2/RGS19/ARRB2
WP111	Electron transport chain: OXPHOS system in mitochondria	-0.437464361	-1.6924244	0.000925836	0.03333911	SURF1/UQCR11/ATP5PF/COX6C/ATP5MC3/ATP5F1E/NDUFA3/NDUFA9/NDUFS3/NDUFB3/NDUFB6/NDUFS5/NDUFC1/COX5B/UQCR10/UCP2/NDUFA2/SDHC/NDUFV2/NDUFA4/NDUFC2/ATP5MF/NDUFB10/NDUFS6/ATP5MC2/NDUFA12/NDUFAB1/COX7C/ATP5PO/NDUFB8/ATP5MC1/ATP5MG/COX6B1/COX8A/UQCRFS1/UQCRQ/UQCRH/NDUFB9/COX6A1/ATP5F1D/COX7A2/ATP5IF1/NDUFB1/ATP5ME/NDUFB2/COX5A
WP3617	Photodynamic therapy-induced NF-kB survival signaling	0.590758638	1.79722277	0.000995197	0.03333911	IL1B/TNF/BCL2L2/IL1A/BIRC3/CHUK/MMP1/CXCL8/ICAM1/CFLAR/VEGFA/PTGS2
WP183	Proteasome degradation	0.500505708	1.75027654	0.001143104	0.03350247	PSMC1/PSMD13/UBE2D3/PSMD11/PSMD1/UBB/PSMB7/PSMC4/PSMD7/PSMD5/PSMB2/PSMC6/PSMC3/HLA-G/PSMB4/PSMD8/RPN2/PSMD4/PSMD6/PSMD2/PSMA3/PSMD12/UBC
WP3613	Photodynamic therapy-induced unfolded protein response	0.615632709	1.79557196	0.001199195	0.03350247	NFE2L2/ATF4/PPP1R15A/DNAJC3/ATF3/DNAJB9/DDIT3/WARS1
WP466	DNA replication	-0.52592362	-1.7935706	0.001214375	0.03350247	ORC1/RFC3/CDC7/POLD1/MCM4/RPA1/MCM7/POLD4/CDC45/RPA3/RFC5/CDC6/POLE2/MCM10/GMNN/RFC4/MCM5/PRIM1/RFC2/POLD3/CDT1/MCM3
WP4922	Mitochondrial complex IV assembly	-0.563452607	-1.7858411	0.001824908	0.04754899	TACO1/SURF1/COX6C/COX18/SMIM20/COX5B/COA6/NDUFA4/COX14/COA3/COX7C/COX6B1/COX8A/SCO2/COX6A1/COX7A2/TMEM177/COX5A/PNKD/COX20
WP530	Cytokines and inflammatory response	0.692544489	1.85510583	0.002077794	0.0489412	IL1B/TNF/IL1A/IL15
WP2359	Parkin-ubiquitin proteasomal system pathway	0.487657976	1.70534782	0.002087045	0.0489412	PSMC1/PSMD13/HSPA9/PSMD11/CUL1/TUBA4A/PSMD1/HSPA1B/HSPA1A/TUBB6/PSMC4/PSMD7/PSMD5/TUBB4A/PSMC6/HSPA2/PSMC3/HSPA6/HSPA1L/UBE2J1/HSPA4/HSPA8/PSMD8/PSMD14/PSMD4/PSMD6/CASP1/PRKN/UBE2G1/PSMD2

Table.S7
GSEA Pathways by combinatorial treatment (diHOME + Pnut) compared to DMSO

ID	Description	Enrichment Score	NES	Unadjusted P	Adjusted P	Core Enrichment Genes
WP183	Proteasome degradation	0.72383578	2.68365889	1.00E-10	1.56E-08	PSMA3/PSMC6/UBE2D1/PSMA4/PSMA6/PSMC2/PSMB7/UBE2D3/PSMD12/PSMB4/UBC/PSMB5/PSMA1/PSMD6/UBE2D2/PSMD2/PSMA7/PSMB2/PSMC1/PSMA2/PSMD11/PSMC5/PSMD7/PSMB1/PSMB3/PSMB6/PSMC4/UBB/PSMD1/PSMD8/PSMD13/PSMA5/UCHL1/UBE2B/PSMC3/H2AZ1/PSMD9/PSMD3
WP2882	Nuclear receptors meta-pathway	0.49164913	2.19959481	1.00E-10	1.56E-08	CYP1A1/GCLM/NQO1/TXN/SRXN1/GSTM3/CCL20/CDKN1C/PRDX1/HBEGF/KTN1/TXNRD1/HSPA1A/HMOX1/DNAJB1/SERPINB2/BLVRB/ANGPTL4/TNF/CDC42EP3/SQSTM1/FTL/SLC7A11/ALAS1/KEAP1/MAFG/SRGN/ME1/VDR/PTGS2/SLC19A2/NFE2L2/ANKRD1/GSTP1/CPEB4/IL1B/NR3C1/CDC37/MGST1/GSR/PGD/GADD45B/PSMC5/HSP90AA1/B3GNT5/HSP90AB1/PTGR1/IRS2/CYP1B1/SLC2A1/SLC2A3/THBD/PTGES3/ARL5B/ABCC2/PLK2/PDGFB/CPT1A/SLC5A10/JUN/TGFA/SLC39A14/POLK/TGFB2/DNAJC7/GGT1/SCD/SLC5A3/SLC2A14/SLC7A5/CUL1/FTH1/GCC1
WP477	Cytoplasmic ribosomal proteins	0.6593373	2.58456746	1.00E-10	1.56E-08	RPS20/RPS13/RPL24/RPL41/RPS3A/RPS27/RPL9/UBA52/FAU/RPL19/RPL21/RPS21/RPS26/RPL35A/RPS28/RPL7/RPL27/RPL14/RPL37/RPLP1/RPL30/RPL34/RPL12/RPL11/RPS25/RPS7/RPS3/RPS23/RPS11/RPL38/RPL28/RPL23/RPS9/RPL8/RPL31/RPL18/RPL23A/RPS12/RPS27A/RPLP2/RPS19/RPS6/RPLP0/RPS5/RPS29/RPL27A/RPL6/RPL4/RPL32/RPSA/RPL18A/RPL17/RPL13/RPL13A/RPS15A/RPS8/RPL37A/RPS2/RPL3/RPS14/RPS15/RPL7A/RPL36
WP2059	Alzheimer's disease	0.45966215	2.05637521	2.07E-09	2.42E-07	ATF4/PSMA3/PSMC6/IL1A/SEM1/PSMA4/RTN3/PSMA6/TNF/PSMC2/RB1CC1/PSMB7/TRAF2/PSMD14/PSMD12/GAPDH/PSMB4/PTGS2/PSMB5/PLCB1/PSMA1/PSMD6/IL1B/PPP3CC/PSMD2/PSMA7/PSMB2/PSMC1/PSMA2/PSMC5/ADRM1/RAF1/PSMD7/CSNK2A1/XBP1/DDIT3/EIF2AK3/VMP1/PSMB1/PSMB3/PIK3C3/PSMB6/CYCS/PSMC4/IRS2/MAP2K1/PSMD1/PSMD8/PSMD13/ATG101/PIK3R3/TUBA4A/CDK5R1/CHUK/PSMA5/CSNK1A1/PPID/TUBA1C/APC2/PSMC3/MCU/MAPK8/ATP5MD/NRBF2/IDE/PSMD9/ERN1/PSMD3/ITPR1/ATG14/ADAM17/APP/KIF5B/TUBB2A/TUBB4A
WP2359	Parkin-ubiquitin proteasomal system pathway	0.63309973	2.34725024	1.49E-08	1.40E-06	HSPA6/PSMC6/HSPA1B/HSPA1A/PSMC2/PSMD14/PSMD12/HSPA9/PSMD6/PSMD2/PSMC1/PSMD11/PSMC5/PSMD7/UBE2J2/HSPA2/PSMC4/PSMD1/PSMD8/HSPA8/PSMD13/HSPA1L/TUBA4A/HSPA4/CASP1/UBE2L3/TUBA1C/PSMC3/UBE2J1/PSMD9/PSMD3/HSPA5/CUL1/TUBB2A/TUBB4A/PSMD4
WP4558	Overview of interferons-mediated signaling pathway	-0.8562584	-2.4163617	6.88E-08	5.38E-06	TYK2/IFNAR2/IFNGR2/JAK2/STAT2/IRF9/PIK3R1/IFNGR1/IFNLR1/IL10RB
WP2884	NRF2 pathway	0.5231473	2.08608623	7.21E-07	4.83E-05	GCLM/NQO1/TXN/SRXN1/GSTM3/PRDX1/HBEGF/TXNRD1/HSPA1A/HMOX1/DNAJB1/BLVRB/SQSTM1/FTL/SLC7A11/KEAP1/MAFG/ME1/NFE2L2/GSTP1/GSR/PGD/HSP90AA1/HSP90AB1/PTGR1/SLC2A1/SLC2A3/ABCC2/PDGFB/SLC5A10/TGFA/SLC39A14/TGFB2
WP2880	Glucocorticoid receptor pathway	0.58738808	2.13454338	1.40E-06	7.86E-05	CCL20/CDKN1C/KTN1/ANGPTL4/CDC42EP3/SRGN/PTGS2/SLC19A2/ANKRD1/CPEB4/NR3C1/GADD45B/HSP90AA1/B3GNT5/THBD/PTGES3/ARL5B/PLK2/JUN
WP3888	VEGFA-VEGFR2 signaling pathway	0.3528992	1.67341168	1.51E-06	7.86E-05	TXN/ATF4/ANXA1/HBEGF/P4HA2/INPP5K/TBCA/HSPA1A/ALDOA/DNAJB4/VEGFA/GATA2/WASF1/PPP2CA/DNAJA1/FXR2/RCAN1/ATP6V1E1/SRP54/DNAJB9/BIN1/GAPDH/PTGS2/ELOC/MMP10/ARF4/PRDX2/EIF2A/PGD/ITGB3/CXCL8/PSMD11/NR4A2/CHAC1/HSP90AA1/RAF1/CALU/TKT/EIF2AK3/LRRFIP2/CYCS/MAP2K3/MAP2K1/RND1/EWSR1/RPL7/CSRP2/RPL27/SARS1/NR4A1/RHOA/LUC7L/DPM1/PDE4DIP/RPS6KA5/SDCBP/CDC42/JUN/TUBA1C/NDRG1/ACP1/SPIRE1/MAPK8/RPS11/CCT7/STAM/EIF4E/RACK1/HERPUD1/ZC3H15/ITGAV/CRK/FRS2/LARP7/EIF3F/SSR3/ERN1/CTNNA1/LDHA/CREBBP/HSPB1/NAPA/ITCH/HGS/EIF3H/IER5/PRRC2C/SLC2A14/JAG1/ICAM1/RAP1B/RPLP2/BSG/RPS6/TRIP4/PLAUR/DUSP5/IGFBP7/ACTG1/RAB5A/RBM39/AFDN/STIP1/PSMD4/SCUBE2/LRRC59/NAP1L1/ARPC5L/PPM1G/IQGAP1/TMEM170A/MAPKAPK2
WP408	Oxidative stress response	0.74250825	2.22973819	2.27E-06	0.00010649	CYP1A1/NQO1/MT1X/TXNRD1/SOD1/HMOX1/NFE2L2/MGST1/GSR
WP4396	Nonalcoholic fatty liver disease	0.43447045	1.85262634	5.09E-06	0.00021696	SDHB/ATF4/IL1A/COX6B1/NDUFA4/NDUFS5/TNF/TRAF2/UQCRH/NDUFB4/IL1B/UQCRC2/CXCL8/XBP1/DDIT3/EIF2AK3/VMP1/COX6C/PRKAG2/UQCRC1/CYCS/IRS2/NDUFS4/PIK3R3/COX7A2L/UQCRB/CDC42/UQCRHL/JUN/ADIPOR1/MAPK8/NDUFS6/COX4I1/COX7A2/ERN1/ITCH/NDUFA6/COX5A/NDUFA10/NDUFB9/SOCS3/EIF2S1/NDUFA3/NDUFS1/UQCRQ/NDUFB7
WP2873	Aryl hydrocarbon receptor pathway	0.68869164	2.1647453	9.73E-06	0.00038022	CYP1A1/NQO1/SERPINB2/TNF/NFE2L2/IL1B/CDC37/MGST1/HSP90AA1/CYP1B1/PTGES3/JUN/POLK
WP2446	Retinoblastoma gene in cancer	-0.4649619	-1.9823317	1.21E-05	0.00041769	TFDP1/PCNA/CDC7/POLE/SMC3/TFDP2/HLTF/CDK6/CDC25B/TOP2A/MCM7/CDK2/STMN1/TP53/DHFR/CCDC6/SIN3A/TYMS/RPA1/POLE2/MYC/CCNA2/CCND1/RFC5/NPAT/PRKDC/FANCG/RFC4/CCNB2/POLD3/E2F2/MCM3/MCM4/DNMT1/BARD1/ABL1/CDC25A/MSH6/SKP2
WP111	Electron transport chain: OXPHOS system in mitochondria	0.49569893	1.94873235	1.25E-05	0.00041769	SDHB/COX17/COX6B1/NDUFA4/NDUFS5/UQCRH/NDUFB4/UQCRC2/COX6C/UQCRC1/NDUFS4/COX7A2L/UQCRB/ATP5PF/ATP5F1B/NDUFS6/COX4I1/COX7A2/NDUFA6/COX5A/NDUFA10/NDUFB9/ATP5F1E/NDUFA3/NDUFS1/UQCRQ/NDUFB7/SDHD/COX6A1/NDUFAB1/COX7C/NDUFA8/NDUFA9/ATP5PO/NDUFB5
WP3945	TYROBP causal network in microglia	-0.5153081	-2.0213055	2.93E-05	0.00091542	PPP1R18/IGSF6/CREB3L2/RNASE6/CD37/C1QC/ITGAM/GAPT/GAL3ST4/SAMSN1/MAF/TGFBR1/TCIRG1/IL10RA/GIMAP2/ZFP36L2/TNFRSF1B/LHFPL2/RPS6KA1/LYL1/CD84/APBB1IP/SLC7A7/STAT5A/NRROS/TMEM106A/SH2B3/ADAP2/PYCARD/LOXL3
WP4803	Ciliopathies	-0.3773653	-1.7503548	3.40E-05	0.00099576	CCDC151/BBS1/PKD2/B9D1/PLK4/IFT52/NEK2/WDR34/TMEM216/DNAAF2/BBS5/PDE6D/IFT81/CEP78/CEP83/CEP41/KIZ/CCDC28B/NME9/SDCCAG8/PKD1/HYLS1/DYNC2H1/NPHP4/TMEM231/TTC8/CEP19/NEK9/IFT57/SPATA7/ALMS1/UNC119/TTLL5/C8orf37/TMEM237/BBS2/ICK/IFT122/POMGNT1/SPAG1/NPHP1/CEP120/CFAP298/PIK3R4/ANKS3/CPLANE1/INPP5E/KIAA0586/TRIM32/NEK8/RSPH3/MKS1/IFT172/TMEM107/BBS10/RAB23/DNAAF1/EFHC1/SCLT1/TCTN2/WDR35/TCTN3/TBC1D32/TCTN1/CEP164/CLUAP1
WP3612	Photodynamic therapy-induced NFE2L2 (NRF2) survival signaling	0.69179502	2.0774473	5.21E-05	0.0014368	GCLM/NQO1/SRXN1/HMOX1/NQO2/KEAP1/NFE2L2/GSTP1/ABCC2/JUN/MAPK8
WP4917	Proximal tubule transport	0.61780724	1.98386775	6.61E-05	0.00172341	ATP6V0E1/ATP6V1F/ATP6V1G1/SLC3A2/ATP6V1C1/ATP6V1E1/ATP6V1H/ATP6V1D/ATP6V0D2/ATP6V0B/SLC2A1/ABCC2/ATP6V1A
WP15	Selenium micronutrient network	0.51871655	1.91603472	9.74E-05	0.0024037	TXN/PRDX1/TXNRD1/SOD1/TNF/SEPHS2/SELENOK/PTGS2/IL1B/PRDX2/GPX4/GSR/CTH/SELENOS/SELENOI/PRDX3/SELENOT/GGT1/LDLR/KYNU/ICAM1/SELENOF/SELENOW/PRDX5
WP2586	Aryl hydrocarbon receptor Netpath	0.56233496	1.93554921	0.00011779	0.00276212	CYP1A1/NQO1/CDKN1A/TNF/VEGFA/PTGS2/NFE2L2/CDC37/HSP90AA1/RAF1/CYP1B1/MAP2K1
WP4629	Aerobic glycolysis	0.78422412	1.92445786	0.00021025	0.00468734	ALDOA/GAPDH/TPI1/SLC2A1/HK1/ENO1/LDHA/PKM
WP615	Senescence and autophagy in cancer	0.42912204	1.73212588	0.00021988	0.00468734	CDKN1A/GABARAPL2/IL1A/CCL3/SERPINB2/RB1CC1/SQSTM1/IL6ST/ATG12/ATG5/MAP1LC3B/GABARAPL1/IL1B/CXCL8/RAF1/PIK3C3/THBS1/MAP2K3/MAP2K1
WP4357	NRF2-ARE regulation	0.65719415	1.97354156	0.00030924	0.00630591	GCLM/NQO1/HMOX1/SLC7A11/KEAP1/NFE2L2
WP3996	Ethanol effects on histone modifications	-0.6279468	-2.0213496	0.00038755	0.00750506	ELP3/HDAC5/HDAC2/ACSS2/ALDH1A2/HDAC3/DHFR/ALDH1A3/TYMS/MTHFR/EHMT2/SLC19A1/MTR/HDAC7
WP3	Transcriptional activation by NRF2 in response to phytochemicals	0.7370291	1.94258454	0.00040292	0.00750506	GCLM/NQO1/HMOX1/SLC7A11/KEAP1/NFE2L2
WP3624	Lung fibrosis	0.53548963	1.86191897	0.00041683	0.00750506	MT2A/CCL3/HMOX1/TNF/EGF/NFE2L2/IL1B/CSF2/CXCL8/CXCL2/SPP1/PDGFB/CSF3/TGFA
WP706	Sudden infant death syndrome (SIDS) susceptibility pathways	0.43266662	1.72223127	0.00043206	0.00750506	IL1A/ALDOA/TNF/VEGFA/GATA2/GAPDH/RORA/IL1B/NR3C1/HADHB/NKX3-1/CXCL8/CREM/HADHA/TPPP/YWHAQ/CPT1A/JUN/MBD1/VAMP2/CREBBP/HSPD1/YWHAZ/HIF1A/YWHAG/TSPYL1
WP4536	Genes related to primary cilium development (based on CRISPR)	-0.3702148	-1.6580698	0.00045567	0.00760404	BBS2/TTC30B/ICK/IFT122/FUZ/IQCE/DYNLL1/TXNDC15/CEP120/CPLANE1/INPP5E/MKS1/CBY1/IFT172/TMEM107/TEDC1/BBS10/RAB23/TEDC2/SCLT1/TCTN2/WDR35/TCTN3/TCTN1/RABL2A/CEP192/FBF1/CLUAP1
WP4197	Immune response to tuberculosis	-0.647047	-2.0206863	0.00047019	0.00760404	MX1/IFIT1/OAS1/PTPN2/TYK2/IFNAR2/IFNGR2/JAK2/STAT2/IRF9/IFNGR1/PSMB8
WP534	Glycolysis and gluconeogenesis	0.56892817	1.85972302	0.00061112	0.00955377	MPC2/ALDOA/GAPDH/TPI1/ENO2/DLD/SLC2A1/SLC2A3/HK1/LDHAL6B/ENO1/PFKP/HK2/LDHA/PKM
WP3613	Photodynamic therapy-induced unfolded protein response	0.62380172	1.92454431	0.00077376	0.01170619	ATF4/DNAJB9/PPP1R15A/NFE2L2/EIF2A/XBP1/DDIT3/EIF2AK3/DNAJC3/TRIB3/ERN1/HSPA5
WP1946	Cori cycle	0.77613346	1.84915201	0.00113062	0.01657065	TALDO1/ALDOA/GAPDH/TPI1/SLC2A1/HK1/PFKP/LDHA
WP4241	Type 2 papillary renal cell carcinoma	0.586989	1.87426756	0.0018917	0.0268851	CDKN1A/VEGFA/EGLN3/ELOC/SLC2A1/PDGFB/TGFA/PRCC/TGFB2/ELOB/SFPQ/CREBBP/CTSK/HIF1A
WP410	Exercise-induced circadian regulation	0.49265682	1.71298758	0.0023377	0.03224651	GSTM3/PSMA4/CRY1/DNAJA1/GSTP1/VAPA/HSPA8/NCOA4/H2BC15/IDI1/NCKAP1/PPP1R3C/HERPUD1/SUMO1/AZIN1/CRY2/ZFR
WP4313	Ferroptosis	0.53653689	1.73631331	0.00253414	0.03395754	GCLM/HMOX1/FTL/SLC7A11/SLC3A2/ATG5/MAP1LC3B/GPX4/VDAC2/VDAC3/NCOA4/ACSL3/PRNP/SLC39A14
WP623	Oxidative phosphorylation	0.4783811	1.71086349	0.00270568	0.03520336	NDUFA4/NDUFS5/NDUFB4/NDUFS4/ATP5PF/ATP5F1B/NDUFS6/NDUFA6/NDUFA10/NDUFB9/ATP5F1E/NDUFA3/NDUFS1/NDUFB7/NDUFAB1/NDUFA8/NDUFA9/NDUFA11/ATP5PO/NDUFB5
WP4969	RAS and bradykinin pathways in COVID-19	0.68974646	1.7781691	0.00286137	0.03520336	IL1A/TNF/VDR/IL1B/RHOA
WP4946	DNA repair pathways full network	-0.3363875	-1.5398216	0.00289252	0.03520336	DDB2/FEN1/PCNA/POLE/LIG1/FANCC/PMS2/CCNH/MSH3/GTF2H2C/BRIP1/GTF2H2/SMUG1/ERCC2/BRCA1/RPA1/ERCC4/USP1/RFC2/POLI/POLE2/ATM/REV1/RFC5/POLD1/FANCF/MPG/NTHL1/PRKDC/FANCE/PARP1/FANCG/RFC4/XPA/POLD3/APEX1/FAAP100/ATR/MSH2/POLM/UNG/FANCA/XPC/MSH6
WP3617	Photodynamic therapy-induced NF-kB survival signaling	0.55217357	1.76611873	0.00301824	0.03520336	IL1A/MMP1/TNF/VEGFA/CFLAR/PTGS2/IL1B/CSF2/CXCL8/CXCL2/CHUK
WP2864	Apoptosis-related network due to altered Notch3 in ovarian cancer	0.45843509	1.65955222	0.00309123	0.03520336	NQO1/CDKN1A/IER3/TNF/SQSTM1/ANXA5/THBS1/RNF7/IL7R/RIPK2/ERN1/CTNNA1/HSPA5/HSPB1/HSPD1/CUL1/APP/SOCS3
WP2828	Bladder cancer	0.52513415	1.7165683	0.00315919	0.03520336	CDKN1A/HBEGF/MMP1/VEGFA/EGF/CXCL8/RAF1/THBS1/MAP2K1/ERBB2/RPS6KA5
WP516	Hypertrophy model	0.65918376	1.82082699	0.00322348	0.03520336	HBEGF/IL1A/VEGFA/ANKRD1/IFRD1/EIF4E/ZEB1
WP3614	Photodynamic therapy-induced HIF-1 survival signaling	0.55053873	1.76088973	0.0032276	0.03520336	BNIP3/VEGFA/PTGS2/BNIP3L/SLC2A1/SLC2A3/HK1/PMAIP1/TGFA/LDHA/MCL1/PKM/SLC16A1/HIF1A
WP3529	Zinc homeostasis	0.57804155	1.81694193	0.00337331	0.0359564	MT1X/MT1G/MT2A/MT1A/MT1E/SLC30A5/MT1M/MT1F/SLC30A1/MT1H/SLC39A14
WP3937	Microglia pathogen phagocytosis pathway	-0.4570856	-1.6468784	0.00411091	0.04284487	C1QB/VAV3/NCF4/FCGR1A/LYN/ARPC1B/RAC2/C1QC/ITGAM/TREM2/SIGLEC7/HCK/VAV1/SYK/PIK3R1/PIK3CG/PIK3R6/PLCG2/PIK3CD
WP4211	Transcriptional cascade regulating adipogenesis	0.69632567	1.7087582	0.00428656	0.04358054	GATA2/CEBPG/DDIT3/KLF5/PPARG
WP4022	Pyrimidine metabolism	-0.3757226	-1.5696991	0.00436735	0.04358054	RRM2/DCK/TK1/NME4/NT5M/TYMP/POLR3C/POLD4/POLD2/UMPS/POLR2D/POLR3GL/NME3/PRIM1/POLR3B/UCKL1/CTPS1/POLR3K/DTYMK/POLE/NME6/POLR2I/POLR1E/TYMS/POLE2/TWISTNB/DUT/DCTD/POLD1/ENTPD1/DCTPP1/PNPT1/POLR1B/POLD3/POLR2J2/POLR1A/TK2/UCK2/CAD/DHODH
WP395	IL-4 signaling pathway	-0.4373457	-1.6400002	0.00466627	0.04493779	JAK3/IL4R/FES/TYK2/JAK2/PIK3R1/STAT5A/INPP5D/STAT6/IKBKB/PIK3CD
WP107	Translation factors	0.4682161	1.64216563	0.00469499	0.04493779	EIF1/EIF5/EIF3E/EIF2S2/EIF3I/EIF2AK3/EEF1D/EIF6/ETF1/EIF4E/EIF4H/EEF1A1/EIF3F/EIF3H/EIF2S1/EIF3J/EIF2B2/EEF2/EIF3G
WP4300	Extracellular vesicles in the crosstalk of cardiac cells	0.65698622	1.78602856	0.00492305	0.04617821	ETS2/SOD1/EGF/SPP1/CD63/HSPB1

Table.S8

Genes by 3EH vs vehicle

Gene	log2FC	Unadjusted P	Adjusted P
Rln3	2.56535718	9.06E-07	0.01281168
9830107B12Rik	3.86906732	2.27E-06	0.01607123

Table.S9
Genes by CRA vs vehicle

Gene	log2FC	Unadjusted P	Adjusted P
Il4i1	2.74822806	7.64E-08	0.00096354
Slc6a20a	3.58889924	1.36E-07	0.00096354
Rnase2a	10.1545731	2.10E-07	0.00099149
Rln3	2.78363587	4.22E-07	0.00108419
Chia1	4.12818468	5.18E-07	0.00108419
9830107B12Rik	4.53172892	5.25E-07	0.00108419
Ctla4	4.39053374	6.43E-07	0.00108419
Fcer2a	2.7428842	6.52E-07	0.00108419
Clec4a3	1.94928743	6.90E-07	0.00108419
Lat	2.13026354	7.78E-07	0.00110017
Ptgdr	5.55280167	1.32E-06	0.00150565
Lemd1	4.88558486	1.35E-06	0.00150565
Lck	1.70202731	1.38E-06	0.00150565
Ptprcap	1.75786231	1.55E-06	0.00156474
F10	2.63933384	1.83E-06	0.00172531
Serpind1	2.51688661	2.33E-06	0.00205645
Msx3	6.87944178	2.65E-06	0.00212147
Il10	4.76720637	2.70E-06	0.00212147
Chil3	2.17049286	3.28E-06	0.00236269
Cxcl3	5.45936594	3.34E-06	0.00236269
Prg2	6.0382576	3.76E-06	0.00249002
Slc26a4	6.938268	3.91E-06	0.00249002
Tarm1	6.82456463	4.05E-06	0.00249002
Ly6i	4.03058206	4.37E-06	0.00256605
Il27ra	1.59831916	4.54E-06	0.00256605
Bex6	3.74155822	4.89E-06	0.0026067
H2-DMb1	1.14042157	4.98E-06	0.0026067
Scimp	2.18052621	5.17E-06	0.00261262
Septin1	1.40001968	5.61E-06	0.00261378
Cd209e	7.12956435	5.71E-06	0.00261378
Fcgr2b	2.31083899	5.73E-06	0.00261378
Havcr2	1.42840355	6.88E-06	0.00303876
Cd3e	2.25336935	7.29E-06	0.00309358
Ikzf3	1.15259454	7.44E-06	0.00309358
Ltb	1.72502008	8.44E-06	0.00316159
Ifi30	2.18720176	8.48E-06	0.00316159
Pde6h	1.18298007	8.52E-06	0.00316159
Cd3g	1.97353366	8.63E-06	0.00316159
Tnfsf13b	1.58147544	9.11E-06	0.00316159
Icos	2.37827792	9.36E-06	0.00316159
Tigit	3.45622206	9.37E-06	0.00316159
Gm29695	3.83899968	9.60E-06	0.00316159
Pdcd1	3.31157511	9.62E-06	0.00316159
Il1b	2.958247	9.94E-06	0.00319441
Rac2	1.57359699	1.02E-05	0.00320599
Sla	1.37025234	1.25E-05	0.00385648
Aqp9	2.2289136	1.30E-05	0.00390427
Tnfrsf9	5.38184332	1.33E-05	0.00390506
Lag3	2.16257038	1.35E-05	0.00390506
Gimap7	2.01893256	1.41E-05	0.00390506
Ctss	1.47607452	1.42E-05	0.00390506
Ccl8	5.42106624	1.44E-05	0.00390506
Gm21188	1.6976695	1.47E-05	0.00392277
Nfkbiz	1.26801704	1.60E-05	0.00420123
Gm8113	2.93362521	1.65E-05	0.00423618
Cd3d	2.10063864	1.71E-05	0.00431277
Cst7	2.83869653	1.92E-05	0.00469236
Chil5	2.69769866	1.93E-05	0.00469236
Arhgdib	1.21621964	2.03E-05	0.00486002
Gapt	2.66932369	2.07E-05	0.004874
Cxcl2	3.48824423	2.36E-05	0.0054804
Ms4a4c	1.53553129	2.42E-05	0.00552753
C3	1.91641476	2.62E-05	0.00560091
Tnfrsf4	2.7479263	2.62E-05	0.00560091
Saa1	5.41651119	2.66E-05	0.00560091
Oas1g	2.36385346	2.66E-05	0.00560091
Gpr84	5.00808408	2.68E-05	0.00560091
Tfec	2.16041539	2.69E-05	0.00560091
Ms4a8a	1.34166497	2.84E-05	0.00570334
Oas1a	1.91820981	2.86E-05	0.00570334
Tbxas1	1.5208866	2.91E-05	0.00570334
Capn9	6.04394331	2.95E-05	0.00570334
Matk	2.53279606	2.96E-05	0.00570334
Gm525	2.9914257	2.99E-05	0.00570334
Ccl24	7.12898658	3.13E-05	0.00589362
Htr7	2.31058548	3.26E-05	0.0060599
Sdf2l1	2.39202853	3.60E-05	0.00660624
Chil1	1.76676535	3.73E-05	0.00675513
Fgr	1.29580262	3.81E-05	0.00675513
Depp1	-1.3819988	3.82E-05	0.00675513
Ms4a4a	2.98743398	3.89E-05	0.00678483
Mmrn1	-1.6233955	3.97E-05	0.00685135
Samsn1	1.6352775	4.03E-05	0.00685733
Cideb	1.8346795	4.11E-05	0.0069134
Ly86	1.51065942	4.26E-05	0.007012
Socs1	2.64195526	4.27E-05	0.007012
Il21r	1.86931997	4.41E-05	0.00715775
Prkcb	1.02503397	4.86E-05	0.0078123
Spi1	1.45083595	4.92E-05	0.0078123
Scin	3.67967235	5.06E-05	0.00792992
Esyt3	-2.0186214	5.13E-05	0.00792992
Chst13	3.9960939	5.16E-05	0.00792992
Lair1	1.43884185	5.30E-05	0.00798516
Selplg	1.10591323	5.31E-05	0.00798516
Lilra6	1.47538597	5.44E-05	0.00802005
Cyp4f18	1.73180543	5.45E-05	0.00802005

Cd200r1	1.28109853	5.52E-05	0.00803933
Ccdc88b	1.09147364	5.73E-05	0.00826006
Mefv	3.14474096	6.00E-05	0.00839029
Lpxn	1.91253065	6.03E-05	0.00839029
Cd48	1.11981105	6.03E-05	0.00839029
Olfm1	1.1269172	6.05E-05	0.00839029
Slc5a1	2.9433194	6.16E-05	0.00843042
Pla2g4c	5.44131012	6.25E-05	0.00843042
Kynu	1.61618106	6.29E-05	0.00843042
Tnip3	2.5704767	6.38E-05	0.00843042
Klhl23	-0.684608	6.38E-05	0.00843042
Zc3h12d	1.69966079	6.74E-05	0.0088159
Marchf1	0.91969436	7.19E-05	0.00932276
Lilr4b	1.68870602	7.58E-05	0.00973932
Izumo1r	3.10912404	7.87E-05	0.01000124
Gpr183	1.18480826	7.95E-05	0.01000124
Pkib	1.34417436	7.99E-05	0.01000124
Ccr3	4.17444216	8.16E-05	0.01001475
Ncf2	1.1599798	8.22E-05	0.01001475
Hcls1	1.41724997	8.24E-05	0.01001475
Mmp12	4.44899361	8.29E-05	0.01001475
Skap1	1.30200138	8.50E-05	0.01015957
Ccl17	4.16078012	8.59E-05	0.01015957
Fcgr3	1.7170187	8.66E-05	0.01015957
Ccl11	4.18363272	8.81E-05	0.01015957
Slamf6	1.7756457	8.87E-05	0.01015957
Ahsg	2.72617222	8.99E-05	0.01015957
Serpina3f	4.19213588	9.05E-05	0.01015957
Vrk1	0.92052711	9.08E-05	0.01015957
Susd3	1.30702318	9.12E-05	0.01015957
Dok2	2.38474227	9.13E-05	0.01015957
Nfil3	1.62971903	9.34E-05	0.01030731
Lrmp	0.93844545	9.41E-05	0.01030731
Cd84	1.23794125	9.68E-05	0.01041132
Fxyd4	6.06620252	9.71E-05	0.01041132
Ppp2r2b	-1.217032	9.79E-05	0.01041132
Serpina3g	4.42562955	9.85E-05	0.01041132
Cd68	1.25838005	9.87E-05	0.01041132
Lcp2	1.35406505	0.000100809	0.01045481
Il21	4.43412498	0.000101785	0.01045481
Pdcd1lg2	5.21533177	0.000101805	0.01045481
Cd6	1.82016189	0.000102063	0.01045481
Gm32742	-2.4230147	0.000103714	0.01049811
Ccl19	1.87769118	0.000103971	0.01049811
Ccl3	3.22390964	0.000106239	0.0106424
Fcrlb	2.75891914	0.000106906	0.0106424
Clec4a2	1.80052439	0.000112633	0.0111341
Spib	1.42712392	0.000114213	0.01116741
Vav1	1.11262357	0.00011455	0.01116741
Derl3	3.57319772	0.000116055	0.0112367
Gpr65	2.209275	0.000118281	0.01137431
Ly9	1.22726835	0.000121381	0.01159353
Phf24	-2.2850629	0.000129265	0.01226371
Ncf4	1.62312235	0.000131187	0.0123631
Cldn8	-1.6571273	0.00013398	0.01254269
Nfkb2	0.96769892	0.000135077	0.01256215
Sh2d2a	1.90950284	0.000137105	0.0126667
Ptpn6	0.88837492	0.000138359	0.0126667
Chil4	10.4901021	0.000138889	0.0126667
Myo1g	0.84747747	0.000139799	0.01266796
Apol7c	2.64489167	0.000140894	0.01268588
Irf4	1.30993995	0.000142095	0.01271304
Pdia6	0.97693788	0.000143867	0.01273688
Cxcl5	5.78163846	0.000144164	0.01273688
Tgtp1	1.70317371	0.000145538	0.01277846
Cd28	1.66401222	0.000147694	0.01288771
Fmo3	-2.5484573	0.000150728	0.01306752
Zap70	1.35461369	0.000151943	0.01306752
Acaa1b	-2.7756274	0.000152528	0.01306752
Il33	1.97213166	0.000153575	0.01307792
Hpx	1.95054096	0.000156033	0.01318867
Cd52	1.72986623	0.000157068	0.01318867
F7	1.2938701	0.000157674	0.01318867
Spaar	0.88192785	0.000160533	0.01325094
Fcer1g	1.46177881	0.000160718	0.01325094
Slfn2	1.47260984	0.000161231	0.01325094
Rhoh	1.12047744	0.000162793	0.01330202
Cxcr6	1.85318063	0.000166425	0.0135013
Fbln2	-2.3575347	0.000167143	0.0135013
Ccr8	2.32181265	0.000170664	0.01370745
Ctsz	1.27924164	0.000176463	0.01408003
Cd209d	2.86216361	0.000177295	0.01408003
Ccr5	2.33471355	0.000182794	0.01431749
Cd79b	1.07796636	0.000183654	0.01431749
Tnfrsf8	2.39195877	0.000185198	0.01431749
H2-M2	3.53891751	0.000185359	0.01431749
Ccl4	3.09101884	0.000185546	0.01431749
Evi2a	0.9935892	0.000186821	0.01431749
Psmb8	1.07296218	0.000187375	0.01431749
Epsti1	1.39735771	0.000195248	0.01483888
Ccr2	1.04616372	0.000200028	0.01511405
Csf3r	2.04167438	0.000201007	0.01511405
Slc2a6	1.46376287	0.000205544	0.01537336
Birc3	0.74724088	0.000207503	0.01539201
Pigr	2.39178069	0.000207971	0.01539201
Ccl12	3.88118791	0.000211567	0.01544094
Bhlha15	2.22591907	0.000212349	0.01544094
Dmp1	4.7528822	0.000212426	0.01544094
Muc5ac	5.01472646	0.000213117	0.01544094

Batf3	3.40222644	0.000214093	0.01544094
Tmem213	3.19793417	0.000217442	0.01560281
Sele	1.71103374	0.000219067	0.01564004
Aif1	2.10797443	0.000221853	0.01564033
Gimap3	1.21324778	0.00022304	0.01564033
Cebpe	4.76519665	0.000223226	0.01564033
Tagap	0.89395393	0.00022441	0.01564033
Slamf8	2.09491865	0.000224603	0.01564033
Coro1a	1.20723671	0.000228208	0.01581349
Noxo1	1.29485747	0.00023075	0.01591165
Csf2rb	1.07732101	0.000233538	0.01602572
Slc7a2	1.48520869	0.000242952	0.01659115
Mmp25	1.55919567	0.000245981	0.01671723
Ms4a7	2.01493069	0.000251121	0.01698494
Mgl2	1.7517596	0.000254181	0.01705798
Serpina3c	1.44231204	0.000254625	0.01705798
Fgfbp1	-2.4808878	0.000255821	0.01705798
Prkar2b	-0.9717725	0.000259869	0.0172465
Ms4a6d	2.68664518	0.000267102	0.01762601
Ccl6	1.54823334	0.000268081	0.01762601
Map4k1	1.39530155	0.000270637	0.01763875
Tspan32	1.07587849	0.00027077	0.01763875
Acad10	-0.7492122	0.000275029	0.01766961
Kcnn4	1.65754475	0.000275081	0.01766961
Tnfrsf18	1.28120785	0.000276198	0.01766961
AA467197	5.62265092	0.000276244	0.01766961
Slc38a4	-1.7216027	0.000279458	0.01779465
Fcgbp	3.31974616	0.000280922	0.01780767
Pcsk6	-2.2936122	0.000284744	0.01796936
Acod1	5.37560319	0.000287029	0.01803306
Jak3	0.79544542	0.000289075	0.01808127
Jaml	1.1695395	0.000292944	0.01824063
Mcoln2	2.14813407	0.000294204	0.01824063
Prkcd	0.69530766	0.00029677	0.01831938
Tifab	1.04101783	0.000298373	0.01833826
Snx20	1.38067039	0.00031267	0.01913381
Tspan14	0.64238298	0.000314538	0.01916516
AB124611	1.08535952	0.000316037	0.01917379
Lcn2	2.96707828	0.000321779	0.01932875
Cd247	0.95171597	0.000322984	0.01932875
Creld2	1.60622807	0.000323027	0.01932875
Ccr1	2.45113641	0.00032406	0.01932875
C4bp	3.07067719	0.00032913	0.01954867
Fbp1	6.82192322	0.000332643	0.01959355
Olfm4	3.95591823	0.000332713	0.01959355
Cish	1.08030188	0.000334044	0.01959355
Slfn4	3.06477825	0.000337603	0.01972047
Retnlg	3.3874002	0.000344474	0.02001113
Tmem171	0.69733874	0.00034541	0.02001113
Il2rb	1.55060077	0.000350236	0.02012649
Saa3	6.9312757	0.000350249	0.02012649
Cd53	1.15504502	0.000355712	0.02035768
Cdk2ap2	0.91081192	0.000359392	0.02045162
Ubd	4.14564782	0.000360247	0.02045162
St3gal4	0.99575836	0.000371542	0.02100846
Lat2	1.2983636	0.000375461	0.02108546
Tmem26	2.24791534	0.000375887	0.02108546
Gdnf	-1.0389977	0.000384453	0.02141204
Tep1	0.53702486	0.000384738	0.02141204
Kcnf1	-2.8882159	0.000393417	0.02180917
Il2ra	2.41298826	0.000395776	0.02185427
P2rx1	1.89504697	0.000402802	0.02215567
Bcap29	1.14265043	0.000405302	0.02219765
Cd79a	1.30207598	0.000406706	0.02219765
Banp	0.67895852	0.000411558	0.02225746
Slc27a3	0.96034466	0.000412333	0.02225746
Ubxn11	0.79911583	0.000412525	0.02225746
Cd69	1.52481469	0.00042228	0.02262597
Ceacam10	2.56030605	0.000422556	0.02262597
Calhm6	2.06596591	0.000425255	0.02268452
Retnlb	6.18738012	0.000436925	0.02321758
Cd300lb	1.50765526	0.000438532	0.02321758
Batf	1.50459573	0.000442101	0.02330238
Ces1g	-2.3422197	0.000443431	0.02330238
Zfp961	0.71356208	0.000445421	0.02332024
Dclk1	1.60967978	0.000448101	0.02337403
Col6a5	4.85262777	0.000453356	0.02356115
Gm4951	0.79099763	0.000455195	0.0235701
Abca8b	-1.4262543	0.00046702	0.02409415
Bcl2a1a	1.03594397	0.000469093	0.02411307
Sec11c	0.78827732	0.00047123	0.02413516
Cd7	1.38641485	0.000474865	0.0241559
Pon1	-2.519027	0.000475052	0.0241559
Vwf	0.89238611	0.000483097	0.02447692
Ms4a6c	1.63420718	0.000485507	0.02451116
5730507C01Rik	0.92946769	0.000491824	0.0247417
Dpyd	-1.9147023	0.000495797	0.02476831
Tmem198	0.68903269	0.000495857	0.02476831
Slc17a9	1.46832739	0.000498761	0.02476949
H2-DMb2	1.42039537	0.000499385	0.02476949
Hk3	2.13458193	0.00052373	0.02588618
Gfra1	-1.3083847	0.000527902	0.02600145
Ggt5	1.06870844	0.000530589	0.02601189
Slc6a15	-1.8363012	0.000531794	0.02601189
Manf	1.78866431	0.000540628	0.02619329
Il7r	1.04814144	0.000541862	0.02619329
Nfe2l3	-1.9722546	0.000544092	0.02619329
Fyb	1.3265712	0.000544795	0.02619329
Calr	0.87239411	0.000544918	0.02619329

Rgs19	0.89876779	0.000548275	0.02619329
Nrros	1.15001819	0.000548473	0.02619329
Ptafr	1.7836919	0.000551064	0.02622843
Arl4a	-0.5745012	0.0005575	0.0263924
Tmem106a	0.96848627	0.000558243	0.0263924
Ptpn7	0.96355604	0.000569265	0.02673588
Gpr171	1.55150847	0.000571612	0.02673588
Armc7	0.71337565	0.000573	0.02673588
Trpv2	0.61490546	0.000573074	0.02673588
Ubash3a	1.84120678	0.000583055	0.02711206
Fermt3	0.86468849	0.000587803	0.02719721
Gpr132	1.25469957	0.000588734	0.02719721
Itgbl1	-1.9524257	0.000592794	0.02729555
Laptm5	0.82686779	0.000595781	0.02734402
Rnf19b	0.91198622	0.000600471	0.02747008
Malt1	1.10109473	0.000612389	0.02792494
Aldh1a3	3.67047304	0.00061796	0.02808839
Pglyrp2	1.15306426	0.000622084	0.02818519
Treml2	1.79014223	0.000625929	0.02826881
Sting1	1.68101161	0.00062894	0.02831434
Dnajb11	0.98814839	0.000635253	0.02850771
Pdia3	0.8324738	0.00063948	0.02860662
Nkg7	1.35758993	0.000645332	0.02877734
Arg2	0.93950573	0.000651812	0.02897488
Cd37	0.89825887	0.000659871	0.02924118
Pola2	0.89113758	0.000664008	0.02933254
Ube2l6	0.73762873	0.000666218	0.02933318
Ccl20	3.95330752	0.00066947	0.02933318
Ptpn22	1.01390347	0.000670247	0.02933318
Acvr2b	-0.8716009	0.000677974	0.02957975
Dbp	-2.3552739	0.000681993	0.02966354
Nlrp12	2.30890423	0.000686826	0.02967691
Nfkbie	1.10995488	0.000687464	0.02967691
Kcnj15	1.59706005	0.000689852	0.02967691
Serpina3n	2.78148751	0.000691457	0.02967691
Hmgcs2	-1.8273712	0.000692797	0.02967691
Pdia4	1.05644918	0.000706732	0.0300946
Galnt6	0.92284577	0.000706806	0.0300946
Cd300ld	1.43028478	0.000710637	0.03016685
Snx8	1.02719284	0.000717366	0.03036134
Magi2	-0.8338669	0.000721473	0.03044401
Ch25h	3.18513232	0.000724003	0.03045985
Gadd45g	1.95468497	0.000745776	0.03128277
Bcl2a1d	1.35763285	0.000751191	0.03141666
Slc35g1	-0.9941418	0.000755276	0.03149435
Gatm	3.14038641	0.000762222	0.03169051
Ngef	-0.9289835	0.000770458	0.03191601
Zfp385b	1.36583012	0.000772162	0.03191601
Ntn5	1.1725109	0.000778282	0.03192809
Il18bp	1.41484448	0.000778501	0.03192809
Tnfsf15	1.18089051	0.000780132	0.03192809
Kmo	1.13798103	0.000781488	0.03192809
Relt	1.23363021	0.000788636	0.03212728
Fgf23	5.27504178	0.000795731	0.0322691
Tnf	1.91187978	0.000796683	0.0322691
H60c	-3.3395688	0.000799347	0.0322845
Rhpn2	-0.7554267	0.000803245	0.0323495
Wdr86	1.62526857	0.000806393	0.03238402
Ktn1	-0.6368156	0.000811796	0.03250865
Cd4	2.10901908	0.000819521	0.03270151
Spn	0.63951507	0.000822288	0.03270151
Cyrib	0.78646631	0.000823553	0.03270151
Lta	1.47294572	0.00082749	0.03276581
Cd19	0.79361603	0.000841689	0.03309344
Col6a2	1.14110623	0.000842754	0.03309344
Zc3h12a	1.45521896	0.000843557	0.03309344
Ccl7	4.61315393	0.000845128	0.03309344
Arhgap30	0.77516422	0.000854951	0.03331557
Cxcr2	2.6877331	0.000855515	0.03331557
Myh7	-2.0246246	0.000876541	0.03404063
Isg20	0.82933878	0.000879243	0.034052
Cd300lf	1.7902189	0.000892089	0.03445512
Angptl2	-1.0550085	0.000905341	0.03487166
Cyp39a1	-0.7392298	0.000910543	0.034952
Mfap4	0.76592597	0.000915124	0.034952
Nfkbid	1.06467054	0.000915836	0.034952
Sgcb	-0.5854802	0.000917317	0.034952
Cdc42ep2	0.75026853	0.000920767	0.03498913
Tm4sf5	1.87352699	0.000934812	0.03542761
Hsh2d	1.27751031	0.000955336	0.03610862
Zfp667	-1.044885	0.000958659	0.03613408
Galnt15	-2.1378378	0.000961122	0.03613408
Lcp1	0.66417761	0.000965374	0.0361436
Retnla	4.76769675	0.000967444	0.0361436
Scara5	-3.3829364	0.000970341	0.0361436
Vldlr	-1.2312842	0.000973496	0.0361436
Fcrl5	2.44446704	0.000976447	0.0361436
Msc	1.30790028	0.000976716	0.0361436
Il4	1.4603914	0.000982699	0.0362247
Clec4b1	1.08045801	0.000984033	0.0362247
Ly6c2	1.12515467	0.000994109	0.0365006
Map3k21	-1.1750545	0.000997666	0.03653627
Wfdc17	2.66970072	0.001005823	0.03654977
Relb	0.90072383	0.001008047	0.03654977
Oas3	1.66368856	0.0010083	0.03654977
Gm8369	1.03431574	0.001008377	0.03654977
Tnfrsf26	1.06466669	0.00101383	0.03665345
Gal	-1.3922758	0.001035576	0.03734413
Cd72	0.49193116	0.001041498	0.03746212

Mfsd4b4	-0.7518688	0.001045894	0.03752477
Disp1	-0.7114036	0.001062336	0.03801817
Mob3a	0.57012594	0.001065254	0.03802635
Ncf1	0.76551402	0.001072953	0.0382047
H2-DMa	0.89247577	0.001080968	0.03834276
Foxl1	-1.0586171	0.001083009	0.03834276
Tnfsf8	2.46961491	0.001086309	0.03834276
Ctsk	1.73661254	0.0010901	0.03834276
Pim1	1.43483428	0.001090393	0.03834276
Ccl9	3.24772524	0.001098132	0.03851909
Uevld	-0.6407207	0.001105514	0.03868206
Nfe2	1.19007129	0.001116831	0.03898155
Orai1	0.9390534	0.001130739	0.03936976
Ctse	1.23148299	0.001146996	0.03978792
Rab3il1	1.57768787	0.001149549	0.03978792
Pnpla5	-4.9632913	0.001151193	0.03978792
H2-T23	0.60308912	0.00116156	0.03984696
H13	0.63491682	0.001162934	0.03984696
Cp	0.72229517	0.001163863	0.03984696
Irf8	0.61645886	0.00116583	0.03984696
Rab37	1.38396956	0.001167526	0.03984696
Efhd2	0.90826966	0.001170279	0.03984696
Wfdc13	2.3298244	0.001172633	0.03984696
Rgs14	0.78609141	0.001186527	0.04015964
Ffar2	1.88228881	0.001187516	0.04015964
Mblac2	-0.6925342	0.001205344	0.04041243
Macrod1	-0.9857927	0.001210018	0.04041243
Hspa5	0.94396257	0.001210278	0.04041243
Camk4	1.47379571	0.001210396	0.04041243
Lst1	1.62145836	0.001212375	0.04041243
Mrgprg	4.51272263	0.001212403	0.04041243
Pafah2	-0.6664537	0.001219396	0.04041243
Zc3h6	-0.5101449	0.001220493	0.04041243
Cyba	1.29138995	0.001222472	0.04041243
Azgp1	-2.2650488	0.001223579	0.04041243
Steap4	1.62885454	0.001232749	0.04062037
Zranb1	-0.559609	0.001238444	0.04071313
Tnfsf14	1.82710984	0.001252996	0.04109595
Rbm14	0.69992942	0.001258074	0.04116698
Ccl22	1.47783726	0.001264168	0.04125412
Rhog	0.6582808	0.001267399	0.04125412
Zbp1	1.63440386	0.001269492	0.04125412
Nid2	-0.9263101	0.001274722	0.04132907
Adamts1	-1.038916	0.001281954	0.04137589
H2-Ab1	1.00734858	0.00128202	0.04137589
Adora3	1.98163416	0.001287485	0.04145762
Apol7e	1.23193716	0.001293146	0.04147255
Neurl3	0.79492141	0.001293817	0.04147255
Arhgap9	0.71382039	0.00131747	0.04213519
Sftpd	1.1693582	0.001328995	0.04232329
Phyhipl	-0.4704627	0.001329339	0.04232329
Ppp1r14d	1.18953408	0.001337828	0.04244514
Ear1	-1.7787105	0.001339172	0.04244514
Cd2	0.97525753	0.001372877	0.04341607
Fcgr1	1.50865477	0.001376153	0.04342255
Phf11b	1.02859815	0.001380916	0.04347578
Enpp6	-1.2780018	0.001391214	0.04370265
Rhobtb1	-0.937633	0.001398767	0.0438425
Atp9a	-0.4594748	0.00140262	0.04385808
Grap2	0.98669362	0.001405469	0.04385808
Irf5	0.80969262	0.001416034	0.04409045
Ctsc	0.77309131	0.001429566	0.04441394
Slc35e3	-0.6868218	0.001455688	0.04504965
Mrc1	0.74226613	0.001456805	0.04504965
Me3	-1.2347045	0.001459588	0.04504965
Gm5150	1.93450528	0.0014674	0.04507998
Pou6f1	-0.7994	0.001468486	0.04507998
Naaa	0.90607602	0.001470138	0.04507998
Cnpy3	0.6082066	0.001479291	0.04526247
Samhd1	0.53200793	0.00148465	0.04532833
Mkrn3	-0.6979067	0.001506243	0.04588849
Ms4a4b	1.13499252	0.001510853	0.04592994
Iars2	-0.5236433	0.001520008	0.04610909
Rasal3	1.17016039	0.001526943	0.04622028
Synj2	-0.8106004	0.001535048	0.04636631
Mapk12	-0.4918506	0.001571799	0.04737516
Sh3bgrl3	0.96748912	0.00158005	0.04752251
Tmc8	1.09153242	0.001599561	0.04779173
Aqp4	-1.8192185	0.00160069	0.04779173
Ccr7	1.343272	0.001604993	0.04779173
Ntn1	-0.5024125	0.00160502	0.04779173
Gpr35	1.44281789	0.001605905	0.04779173
Cep70	-0.8695	0.001620001	0.04810996
C1qb	2.0553212	0.001625038	0.04815836
Slamf1	1.55436043	0.001635046	0.04835359
Kifap3	-0.568804	0.001667501	0.04917661
4921507P07Rik	-1.6285511	0.001669834	0.04917661

Table.S10

Genes by 3EH+CRA vs vehicle

Gene	log2FC	Unadjusted P	Adjusted P
Il4i1	2.90555669	4.47E-08	0.00063244
Slc6a20a	3.74006703	9.19E-08	0.00064005
Rln3	3.13753224	1.36E-07	0.00064005
Fcer2a	3.08118417	2.18E-07	0.00077063
Rnase2a	8.79086496	8.17E-07	0.00191519
Chia1	3.91851095	8.43E-07	0.00191519
Lemd1	5.02521812	1.04E-06	0.00191519
F10	2.77857635	1.14E-06	0.00191519
9830107B12Rik	4.14013874	1.22E-06	0.00191519
Ctla4	4.03454475	1.41E-06	0.00199429
Gm29695	4.69518302	1.56E-06	0.0019993
Ly6i	4.41506268	1.91E-06	0.00224413
Clec4a3	1.72976787	2.09E-06	0.00226789
Msx3	6.87417492	2.66E-06	0.00246103
Chil3	2.1987886	2.91E-06	0.00246103
Il27ra	1.6731895	3.00E-06	0.00246103
Slc26a4	7.13687419	3.03E-06	0.00246103
Nfkbiz	1.5082178	3.43E-06	0.00246103
Scimp	2.2705271	3.59E-06	0.00246103
Il10	4.61272553	3.64E-06	0.00246103
Sla	1.57288418	3.66E-06	0.00246103
Tarm1	6.86707951	3.83E-06	0.00246103
Cxcl3	5.31569676	4.26E-06	0.00261547
Ptgdr	4.83306444	4.69E-06	0.00272397
Fcgr2b	2.35397152	4.85E-06	0.00272397
Ptprcap	1.54537816	5.01E-06	0.00272397
Lck	1.46816484	5.32E-06	0.00278763
Septin1	1.39757179	5.70E-06	0.00287873
Havcr2	1.44447513	6.22E-06	0.0028948
Fgr	1.59180887	6.33E-06	0.0028948
Ltb	1.78094011	6.35E-06	0.0028948
Il1b	3.06919089	7.17E-06	0.00316175
Pde6h	1.19280015	7.92E-06	0.00316175
Gpr84	5.75410166	7.96E-06	0.00316175
Selplg	1.37481463	8.10E-06	0.00316175
H2-DMb1	1.07732213	8.28E-06	0.00316175
Cd200r1	1.59344139	8.40E-06	0.00316175
Lat	1.63881793	8.50E-06	0.00316175
Csf3r	2.941585	9.37E-06	0.00320289
Tbxas1	1.72812088	9.58E-06	0.00320289
Noxo1	1.89311263	9.62E-06	0.00320289
Aqp9	2.29858381	9.90E-06	0.00320289
Mmp25	2.29009652	9.94E-06	0.00320289
Rac2	1.57777673	9.97E-06	0.00320289
Cd3g	1.93046655	1.05E-05	0.00329499
Cd68	1.6145524	1.19E-05	0.00365746
Chil1	2.00697817	1.24E-05	0.00373049
Htr7	2.56474861	1.32E-05	0.00383327
Serpind1	2.06878407	1.35E-05	0.00383327
Pdcd1	3.18488784	1.36E-05	0.00383327
Ccdc88b	1.28419041	1.43E-05	0.00395185
Cd209e	6.3876781	1.51E-05	0.00410051
Ctss	1.46084607	1.55E-05	0.00410051
Tnfrsf9	5.28155014	1.57E-05	0.00410051
Spi1	1.65480668	1.60E-05	0.00410051
Ikzf3	1.04994233	1.69E-05	0.00427152
Matk	2.68011313	1.82E-05	0.00434843
Prg2	5.0511888	1.83E-05	0.00434843
Cxcl2	3.58449069	1.87E-05	0.00434843
Ncf2	1.38192273	1.87E-05	0.00434843
Mefv	3.605098	1.88E-05	0.00434843
Myo1g	1.07325787	1.94E-05	0.0043875
Gm21188	1.64301662	1.96E-05	0.0043875
C3	1.97160349	2.05E-05	0.00451515
Tagap	1.19187294	2.09E-05	0.00451515
Lilr4b	1.96483345	2.11E-05	0.00451515
Csf2rb	1.43117538	2.25E-05	0.00475415
Cd3e	1.96186742	2.45E-05	0.00510026
Ifi30	1.93300522	2.50E-05	0.0051182
Arhgdib	1.18220615	2.59E-05	0.00520192
Lilra6	1.60892301	2.61E-05	0.00520192
Tnfsf13b	1.38928343	2.82E-05	0.00540252
Vav1	1.3151047	2.84E-05	0.00540252
F7	1.59012587	2.87E-05	0.00540252
Ms4a8a	1.34019614	2.87E-05	0.00540252
Capn9	6.00105675	3.13E-05	0.00578861
Cxcr6	2.26749462	3.15E-05	0.00578861
Blk	1.58071659	3.21E-05	0.00582479
Oas1a	1.88392381	3.33E-05	0.00596301
Ccl8	4.91026281	3.37E-05	0.00596301
Tfec	2.09897214	3.45E-05	0.00596717
Prkcb	1.06717367	3.46E-05	0.00596717
Pla2g4c	5.81458136	3.58E-05	0.00609807
Oas1g	2.27543452	3.69E-05	0.0062024
Ncf1	1.17297423	3.86E-05	0.00635062
Cd84	1.38250435	3.86E-05	0.00635062
Bex6	2.947427	3.94E-05	0.00640375
Trpv2	0.85635538	4.14E-05	0.00660461
Ms4a4a	2.96375524	4.16E-05	0.00660461
Mmrn1	-1.5988072	4.52E-05	0.00710009
Slc5a1	3.04222155	4.67E-05	0.0072586
Tep1	0.6959332	4.84E-05	0.0074375
Tmem171	0.8887255	4.94E-05	0.0075122
Cyp4f18	1.74977604	5.00E-05	0.00751278
Cd300lb	1.96256034	5.39E-05	0.00794921
Lair1	1.4356646	5.40E-05	0.00794921

Sele	2.03135257	5.46E-05	0.00795752
Mob3a	0.83565337	5.56E-05	0.0080058
Zc3h12d	1.7328621	5.74E-05	0.0080058
Scin	3.6203893	5.80E-05	0.0080058
Saa1	4.94055138	5.80E-05	0.0080058
Gpr183	1.23057545	5.81E-05	0.0080058
Ptpn6	0.98736471	5.83E-05	0.0080058
Ccl24	6.58384602	6.11E-05	0.0080898
Susd3	1.37145125	6.13E-05	0.0080898
Tcte2	-1.1369906	6.32E-05	0.0080898
Cst7	2.46664595	6.33E-05	0.0080898
Clec4a2	1.93145039	6.33E-05	0.0080898
Lpxn	1.8994749	6.38E-05	0.0080898
AB124611	1.32570005	6.39E-05	0.0080898
Lcp1	0.9430996	6.44E-05	0.0080898
Kif21b	1.2507411	6.46E-05	0.0080898
Fcgr3	1.77897089	6.47E-05	0.0080898
Nfe2	1.71914534	6.63E-05	0.00820658
Nfam1	1.02673965	6.68E-05	0.00820658
Icos	1.88950036	6.74E-05	0.00821452
Slfn4	3.73617822	6.98E-05	0.0083736
Il21r	1.76838902	7.00E-05	0.0083736
Slfn2	1.6307451	7.05E-05	0.0083736
Tbl3	0.98666421	7.41E-05	0.00870058
Hcls1	1.43487822	7.45E-05	0.00870058
Arl4a	-0.7386367	7.81E-05	0.00897453
Olfm1	1.09238059	7.83E-05	0.00897453
Ceacam10	3.16830087	7.88E-05	0.00897453
Ms4a4c	1.33342501	7.96E-05	0.00897453
Slc2a6	1.64609838	8.00E-05	0.00897453
Malt1	1.42196383	8.37E-05	0.00916913
Mcoln2	2.51435171	8.41E-05	0.00916913
Gapt	2.2591382	8.45E-05	0.00916913
Evi2a	1.09496174	8.55E-05	0.00916913
Gm8113	2.41210484	8.64E-05	0.00916913
Cd3d	1.73275346	8.70E-05	0.00916913
Skap1	1.29810268	8.71E-05	0.00916913
Scart1	4.28688687	8.72E-05	0.00916913
Sdf2l1	2.14952527	8.78E-05	0.00916913
Ptafr	2.25599761	8.82E-05	0.00916913
Coro1a	1.35594942	9.02E-05	0.00922798
Tigit	2.64984308	9.05E-05	0.00922798
Chst13	3.7326746	9.07E-05	0.00922798
Pigr	2.64472738	9.30E-05	0.00938855
Tnfrsf4	2.3593729	9.39E-05	0.00939233
Tfrc	0.92571268	9.43E-05	0.00939233
Fcrlb	2.79201267	9.70E-05	0.00959142
Arhgap9	1.00427026	0.00010021	0.00983729
Ncf4	1.66724442	0.000105639	0.01025648
Ntn5	1.52039236	0.000105931	0.01025648
Socs1	2.35264591	0.000111318	0.01070467
Cd300lf	2.343465	0.000114497	0.01093598
Fcer1g	1.52303445	0.000115643	0.0109713
Vrk1	0.89088962	0.000118509	0.01116832
Snx20	1.55853767	0.00012051	0.01128161
H60c	-4.2589907	0.000124872	0.0115907
Gimap3	1.30451588	0.000125451	0.0115907
Slc7a2	1.61226774	0.00012689	0.01164749
Nlrp12	2.86690896	0.000130755	0.01191459
Irf4	1.32236345	0.000131739	0.01191459
Brinp2	1.35050753	0.000132726	0.01191459
Dok2	2.27524269	0.00013357	0.01191459
Kynu	1.47327284	0.000134014	0.01191459
Stard5	0.63400675	0.00014063	0.0122596
Marchf1	0.84628234	0.000141414	0.0122596
Jaml	1.28275999	0.000141842	0.0122596
Ccr3	3.89768705	0.000142328	0.0122596
Gimap7	1.53284532	0.000142399	0.0122596
Dph2	0.73123627	0.000143098	0.0122596
Lcn2	3.28375507	0.000145562	0.01239553
Cd48	1.00311126	0.000148037	0.01246359
Spaar	0.89055973	0.000148545	0.01246359
Smyd5	0.70268835	0.000149614	0.01246359
Galnt6	1.12865493	0.000152347	0.01246359
Ctsz	1.30307784	0.000152393	0.01246359
Nfkb2	0.95304097	0.000152614	0.01246359
Mrc1	1.0051359	0.00015342	0.01246359
Cxcl5	5.73659792	0.000153452	0.01246359
Ptpn7	1.14222726	0.000154296	0.01246359
Ccr1	2.69302286	0.00015543	0.01248388
Zfp385b	1.68415381	0.000157357	0.01251831
Cxcr2	3.36112514	0.00015763	0.01251831
Klhl23	-0.6118705	0.000159077	0.01256264
Mmp12	4.09347121	0.000162459	0.01275844
Lrmp	0.8759835	0.000163807	0.01277244
Ptgs1	1.34090846	0.000164506	0.01277244
Ccl3	3.05108709	0.000165348	0.01277244
Slamf6	1.64090152	0.000167541	0.01287155
Zfp961	0.80795955	0.000170857	0.01305534
Il33	1.93818803	0.00017628	0.01329021
Tnfrsf26	1.34394756	0.000176941	0.01329021
Tgtp1	1.66121787	0.000177465	0.01329021
Tnip3	2.26537312	0.000177692	0.01329021
Slc27a3	1.06878356	0.000180559	0.01343359
Bcan	-1.9178015	0.000182154	0.01348133
Birc3	0.75890112	0.000183696	0.01348481
Cd53	1.25750339	0.000184109	0.01348481
Acod1	5.67557813	0.000187986	0.01369778
Lilrb4a	1.28016787	0.000190482	0.01377237

Fxyd4	5.57558959	0.000190958	0.01377237
Depp1	-1.1332938	0.000193852	0.01390349
Prkcd	0.73400275	0.000194743	0.01390349
Ntn1	-0.6672291	0.000200344	0.01423148
Chil4	9.98019737	0.000206192	0.01447573
B430306N03Rik	3.41130411	0.000206897	0.01447573
Pdcd1lg2	4.77172166	0.00020714	0.01447573
Tmem268	0.89576775	0.000207879	0.01447573
Ly86	1.24167656	0.000210528	0.01457446
Lat2	1.39819808	0.000212162	0.01457446
Cd6	1.65967989	0.000213264	0.01457446
Samsn1	1.33199581	0.000214443	0.01457446
Derl3	3.30476907	0.000216024	0.01457446
Phyhipl	-0.6018652	0.00021632	0.01457446
Slc38a4	-1.7790932	0.000216514	0.01457446
Cd69	1.66156486	0.000218516	0.01463947
Relt	1.46255659	0.000219551	0.01463947
Fcgbp	3.42196587	0.000221998	0.01473319
Acaa1b	-2.6424242	0.000224859	0.01485332
Ccl6	1.57947614	0.000229556	0.01502896
Sh2d2a	1.78895238	0.000229645	0.01502896
Serpina3f	3.7282714	0.00023105	0.01505125
Tnfsf14	2.29438401	0.000233922	0.01509996
Rhoh	1.06955857	0.000234736	0.01509996
Gm525	2.32260396	0.000236873	0.01509996
Muc5ac	4.94542936	0.000237579	0.01509996
Pdia6	0.91657941	0.000237977	0.01509996
Izumo1r	2.70735115	0.000238479	0.01509996
C4bp	3.20033789	0.000239275	0.01509996
Lcp2	1.21361154	0.000241047	0.01514418
Podnl1	2.01483867	0.000248213	0.01532108
Tspan32	1.08784976	0.00024853	0.01532108
Arhgap30	0.91461227	0.000248832	0.01532108
Epsti1	1.35454638	0.000249017	0.01532108
Lsp1	0.72108662	0.000249282	0.01532108
Ccr2	1.01638556	0.000250637	0.01532686
Itgb2	1.05162049	0.000252361	0.01532686
Ctsk	2.11529389	0.000253748	0.01532686
Ly6c2	1.3520341	0.000254645	0.01532686
Psmb8	1.03037623	0.000257181	0.01532686
Cd300ld	1.6371363	0.000257973	0.01532686
Bcl2a1a	1.11961562	0.000259837	0.01532686
Akr1c19	1.22859468	0.00025987	0.01532686
Necab1	-1.900445	0.000259931	0.01532686
Ms4a6d	2.69572537	0.000260218	0.01532686
St3gal4	1.04230372	0.000261834	0.01534285
Ms4a7	2.00258649	0.00026337	0.01534285
Atp6v0d2	1.08651204	0.000263746	0.01534285
Armc7	0.78714341	0.000272738	0.01575786
Ppp2r2b	-1.0695273	0.000273109	0.01575786
Neurl3	0.97989081	0.000279946	0.01606578
Epdr1	-0.748025	0.000280719	0.01606578
Cideb	1.44336752	0.000286131	0.01630948
Rgs14	0.95361489	0.000287626	0.01632884
B3gnt7	1.30357827	0.00029619	0.016673
Laptm5	0.90707279	0.00029715	0.016673
Chil5	1.93290399	0.000297227	0.016673
Map4k1	1.3726667	0.000306946	0.01715014
Kcnn4	1.63105121	0.000311369	0.01729017
Ptgir	1.16715984	0.000311898	0.01729017
Zfp667	-1.2159007	0.000313481	0.01730678
Gtf2ird2	0.64091006	0.000314646	0.01730678
Trpm2	1.72141104	0.000316468	0.01732574
Tnf	2.1642364	0.000317442	0.01732574
Saa3	6.9863751	0.00032974	0.0179277
Sgcb	-0.6706883	0.000337055	0.01825523
Wdr86	1.82730906	0.000338726	0.01827566
Ccl11	3.52580067	0.000341106	0.01833413
Cd247	0.94439982	0.000342575	0.01834332
Ccr5	2.14935832	0.00034722	0.01843137
Apol7c	2.35581146	0.000347976	0.01843137
Ctsc	0.93926789	0.000349624	0.01843137
Spib	1.23734968	0.000350245	0.01843137
Irf5	0.98195922	0.000350738	0.01843137
Nrros	1.21910777	0.000354246	0.01847741
Myo1f	0.90904777	0.00035525	0.01847741
Batf3	3.1857744	0.000355536	0.01847741
Serpina3g	3.75589802	0.000358749	0.01857611
Cd37	0.97408602	0.000361068	0.01862796
Gpr65	1.91295364	0.000365624	0.01878977
Jak3	0.77101866	0.000366863	0.01878977
Ms4a6c	1.69519064	0.000368669	0.01881409
Nfil3	1.36397596	0.000379607	0.01921058
Itgbl1	-2.0724768	0.000379914	0.01921058
Cd19	0.88397832	0.000380515	0.01921058
Nfkbid	1.19895456	0.000383094	0.01927196
Arg2	1.0082188	0.000385869	0.01934269
Ctse	1.42853922	0.000389895	0.01947547
Cyth4	1.19529239	0.000396629	0.01972737
Gm5150	2.31734769	0.00040056	0.01972737
Lax1	1.17710731	0.000400697	0.01972737
Sh2d4b	1.99891586	0.00040161	0.01972737
H2-DMb2	1.46219932	0.000401916	0.01972737
Ggt5	1.10599814	0.00041074	0.02009072
Psd4	0.87207891	0.000419995	0.02047259
Nfkbie	1.18465638	0.00042485	0.02063809
Hk3	2.19279818	0.000428507	0.02065896
Hsdl2	-0.7669839	0.000429184	0.02065896
Il7r	1.08130362	0.000429664	0.02065896

Tmem106a	1.00263891	0.000431368	0.02067057
Ccl19	1.5607197	0.000441327	0.02102466
Rnf19b	0.95053963	0.000441732	0.02102466
H13	0.72402573	0.000450047	0.02134852
Zap70	1.17628218	0.000452676	0.02140144
Fermt3	0.89304413	0.000462822	0.02180672
Col6a5	4.83711784	0.000464334	0.02180672
Acap1	1.09904334	0.000467272	0.02187206
Fyb	1.35332713	0.000469728	0.02191443
Tshz1	-0.6444698	0.000477935	0.02222397
Cd79b	0.95089233	0.000481106	0.02224424
Itgax	1.59114345	0.000481518	0.02224424
Sirpb1b	1.74715787	0.00048454	0.02231095
Smim13	-0.5637178	0.000489153	0.02245023
Acot1	-1.2855827	0.000496604	0.02271841
Fmo3	-2.18151	0.000499891	0.0227316
Apol7e	1.406475	0.000500108	0.0227316
Pirb	0.95464129	0.000504849	0.02281537
Rubcnl	1.07521657	0.000505179	0.02281537
Fam8a1	-0.6000445	0.000511452	0.02302512
Rab37	1.55039756	0.000515943	0.02311534
Esyt1	0.47493468	0.000516727	0.02311534
Hsh2d	1.38898764	0.000521034	0.02319225
Ubxn11	0.77437744	0.000521727	0.02319225
Ly9	1.01464997	0.000529585	0.0234128
Tifab	0.9644942	0.00053071	0.0234128
Lag3	1.37777132	0.000531657	0.0234128
Fggy	-0.7038503	0.000542902	0.02379344
Rsad2	2.0932738	0.000543667	0.02379344
Trim30b	1.50086347	0.000546326	0.02383601
Dmp1	4.19485615	0.000549673	0.02390826
Ube2l6	0.75651461	0.000553532	0.02391656
Sftpd	1.32172708	0.000554655	0.02391656
Tspan14	0.59566086	0.00055494	0.02391656
Mmp9	2.17041726	0.00055788	0.0239612
H2-M2	3.06248043	0.000559366	0.0239612
Ahsg	2.15211031	0.000567143	0.02422097
AA467197	5.10537222	0.000571998	0.02435471
Mkrn3	-0.7993827	0.000575553	0.0244325
Tnfrsf25	1.26534687	0.00058178	0.02447221
Ccl12	3.39692523	0.000582445	0.02447221
Tas1r3	-1.1213811	0.000583691	0.02447221
Kifap3	-0.6598667	0.000585226	0.02447221
Steap4	1.80763485	0.00058596	0.02447221
Sell	1.16922763	0.000587791	0.02447221
Tmem26	2.11676376	0.000588607	0.02447221
Tnfrsf1b	1.01343212	0.000594273	0.02463531
Slc11a2	0.70125319	0.000598539	0.02466984
Dclk1	1.54800651	0.000598596	0.02466984
Aldh1a3	3.67685072	0.000610153	0.02507304
Mrgpra2b	3.54275837	0.000615804	0.02523188
Cd28	1.37939449	0.000621689	0.02537063
Cebpe	4.1598663	0.000624202	0.02537063
Cdk2ap2	0.84574068	0.000624574	0.02537063
Sp5	1.45348875	0.000634119	0.02568453
Slamf8	1.82078382	0.000648269	0.02618266
Ccl17	3.20576046	0.000650709	0.02620633
Wfdc17	2.82943271	0.000663204	0.02658385
Gpr35	1.63607039	0.000663844	0.02658385
Irf8	0.66687249	0.000665977	0.02659392
Slc15a3	0.96140936	0.000669055	0.02664157
Alox5ap	1.18855156	0.000673583	0.02674654
Rhbdf2	0.56058809	0.000676497	0.02678701
Snx8	1.0339939	0.000683838	0.02700207
Tcf24	-1.6073478	0.000693765	0.02731773
Bcap29	1.06158522	0.000698497	0.02742765
Samhd1	0.59155554	0.000703785	0.02755875
Ccl4	2.58691972	0.000714862	0.02790207
Kmo	1.15175385	0.000716501	0.02790207
Prkar2b	-0.8477101	0.000723372	0.02809225
Lrg1	2.13360391	0.000727122	0.02809677
Ch25h	3.18303393	0.000727463	0.02809677
Relb	0.94125489	0.000736275	0.02835961
S100a8	2.64808014	0.000739474	0.02838452
Tnfrsf18	1.12275358	0.000740937	0.02838452
Ldlr	0.61084603	0.000745505	0.02848234
Crmp1	1.87570146	0.000750195	0.02858426
Pla2g15	0.60639941	0.000756001	0.02872803
Cd7	1.30064423	0.000758549	0.02874758
Tmem213	2.70758905	0.00076069	0.02875166
Arhgap25	0.63069203	0.000766125	0.0288323
Ces1g	-2.1739681	0.000766903	0.0288323
Pdia3	0.81066502	0.0007752	0.02906691
Slfn1	2.29757144	0.000787557	0.0292985
Cd209d	2.34981793	0.000787716	0.0292985
Tnfrsf8	1.9745029	0.000788141	0.0292985
Pglyrp2	1.11514272	0.000792792	0.0292985
Treml2	1.73247251	0.000793632	0.0292985
Oas3	1.72055174	0.000793812	0.0292985
Klhl7	-0.5112574	0.000801714	0.02951309
Serpina3c	1.23506953	0.000811199	0.02970969
Cd52	1.39297084	0.000811258	0.02970969
Lta	1.47277864	0.000828162	0.03025039
Ranbp9	-0.8198409	0.000831886	0.03030811
Rab3il1	1.6504101	0.00083615	0.03038512
Tnfrsf13b	0.80333202	0.000844735	0.03061841
Sting1	1.61305346	0.000847582	0.0306226
Ffar2	1.97398943	0.000849184	0.0306226
Susd6	0.4813445	0.000853436	0.03069765

Mfap4	0.77280364	0.000858674	0.03080767
Ptges	1.37783354	0.000861255	0.03082202
Mgl2	1.48555187	0.000868174	0.0308842
Nckap1l	1.04755278	0.000868596	0.0308842
Il21	3.35052674	0.000869547	0.0308842
Naaa	0.97605692	0.000874845	0.0309945
Hrh2	1.57094953	0.00087704	0.03099461
4930430F08Rik	-0.7718394	0.000887443	0.03128403
B430305J03Rik	0.9160576	0.000890786	0.0312986
Actb	0.80919712	0.000892648	0.0312986
AI467606	0.70517944	0.000894499	0.0312986
Tbc1d17	0.5978585	0.000901232	0.03145632
Dnajb11	0.93965881	0.000912891	0.03178478
Pik3cd	0.9201199	0.000917069	0.0318518
Gm15448	1.36356491	0.000922157	0.03195004
Tnfaip8l2	1.07509871	0.00092554	0.03198883
Siglece	1.32061001	0.000931866	0.03212891
S100a9	2.33811196	0.000934201	0.03213107
Pdia4	1.0144398	0.00094528	0.03241808
Clec4e	1.96905147	0.000947575	0.03241808
Il2rb	1.35385636	0.000949426	0.03241808
Ubash3a	1.71944984	0.000955291	0.03243374
Cyrib	0.77020637	0.000955667	0.03243374
Afdn	-0.4526312	0.000956768	0.03243374
Cndp2	0.76761961	0.000963345	0.03252936
Trem1	1.93996108	0.000964191	0.03252936
Gfra1	-1.2029981	0.000969516	0.03255931
Sec11c	0.7135372	0.000971974	0.03255931
Pim1	1.45846772	0.000971988	0.03255931
Isyna1	0.86565743	0.000997328	0.03332913
Creld2	1.37684868	0.001003234	0.03344743
Ppfia2	-1.4926265	0.001015054	0.03376189
Retnlg	2.92229584	0.001017673	0.03376954
Foxa3	2.91648127	0.001020613	0.03378779
Unc93b1	0.60333049	0.001031459	0.03405116
Hmgcs2	-1.7271102	0.001036908	0.03405116
Syk	0.89210372	0.001037112	0.03405116
Sirpb1c	1.60590545	0.001040019	0.03405116
Spn	0.61849746	0.001042373	0.03405116
Krt10	-0.615405	0.001043021	0.03405116
Smpdl3b	0.98845295	0.00105131	0.03421258
Cst8	-2.6578827	0.001052807	0.03421258
Cd244a	1.04665901	0.001065599	0.03450863
Ddost	0.77258589	0.001066799	0.03450863
Jade1	-0.8283266	0.00107347	0.03464513
Kcnab2	1.02448597	0.001087874	0.03503004
Clec5a	2.00791212	0.001097547	0.03523494
Gatm	2.9826172	0.001099222	0.03523494
Tpcn2	0.59673726	0.001105588	0.03535879
Slc17a9	1.31213951	0.001122393	0.03581524
Pkib	0.95017884	0.001130121	0.03582925
Psap	0.61338711	0.001134526	0.03582925
Il2ra	2.08744273	0.001134864	0.03582925
Galnt15	-2.0870653	0.001137966	0.03582925
Efhd2	0.9116686	0.001140212	0.03582925
Nlrp3	1.12048647	0.001140404	0.03582925
Fbn2	-2.5053303	0.001140574	0.03582925
Cd96	1.49884169	0.001146382	0.03593184
Ltb4r1	1.58949176	0.001150288	0.03596227
Pgc	-0.84918	0.001152441	0.03596227
Cd44	0.67997675	0.001157405	0.03603761
Batf	1.31623935	0.001163208	0.03613871
Slc35c2	0.60381612	0.001166243	0.03615355
Gclc	1.06459354	0.001169073	0.03616196
Acot7	0.92362555	0.001184541	0.03654225
Camk4	1.47789024	0.001187294	0.03654225
Ccl7	4.39505256	0.001189123	0.03654225
C1ra	0.59905046	0.001205283	0.03693225
Pon1	-2.2128564	0.001208219	0.03693225
Nr2f2	-0.4224744	0.001209651	0.03693225
Mvp	0.49164218	0.001218883	0.03706208
Gramd1b	0.56272501	0.001219147	0.03706208
Col6a4	1.96910477	0.001231193	0.03730544
Tmem51	0.68915744	0.001232431	0.03730544
Cd200r3	3.39498921	0.001238897	0.03742105
Clec4b1	1.04364906	0.001253859	0.03779222
Traf3	0.57194399	0.001260875	0.03780261
Ghr	-0.5977071	0.001261765	0.03780261
Col6a2	1.07699475	0.001265657	0.03780261
Bhlha15	1.74811214	0.001266242	0.03780261
Calhm6	1.77466191	0.001273547	0.03780261
Cbx2	-0.8710637	0.001274703	0.03780261
Cerkl	1.05905145	0.001275769	0.03780261
H2-DMa	0.87142426	0.001276281	0.03780261
Upk1a	-1.8575195	0.001283134	0.03780261
Retnlb	5.32816635	0.001283471	0.03780261
Olr1	1.11613826	0.00128362	0.03780261
Clptm1l	0.55450151	0.001291823	0.03796508
Adora3	1.97681907	0.001309273	0.03839811
Adora2b	1.12786375	0.001312536	0.03841411
L2hgdh	-0.7981767	0.001318203	0.03850023
Cpne7	1.67893614	0.001325999	0.03860144
Mvd	1.20429074	0.001329065	0.03860144
Cyp39a1	-0.7002688	0.00132986	0.03860144
Fgf23	4.90154675	0.001333784	0.03863601
Mycl	-1.0149817	0.001339566	0.03872413
Cd72	0.47423605	0.001343653	0.03876303
Aif1	1.64856154	0.0013546	0.03899923
Abca8b	-1.2280596	0.001365668	0.03916981

Hspa5	0.92755053	0.001366067	0.03916981
Tmie	-1.1890251	0.001373027	0.03928968
H4c8	-1.1334436	0.001389754	0.03968622
Gm14548	1.29801464	0.001397884	0.03968622
Tmprss4	0.91100804	0.001400036	0.03968622
Txndc11	0.56021573	0.00140059	0.03968622
Tmem198	0.5958559	0.001400921	0.03968622
Sftpa1	0.75677856	0.0014042	0.03969955
Tbc1d10c	1.02786867	0.001412061	0.0398421
Srp72	0.43887094	0.001418191	0.03993535
Clec4d	2.1154426	0.001421343	0.03994455
Oasl1	1.76101497	0.00143209	0.0401367
Cd79a	1.09285529	0.001433859	0.0401367
Tob1	-0.6561438	0.001442743	0.04030557
Scara5	-3.1925764	0.001451289	0.04044902
Nptxr	-1.1393926	0.001453601	0.04044902
Parvg	0.78498587	0.001472549	0.04085948
Slc2a3	0.98336826	0.001474132	0.04085948
Ripor2	0.73535923	0.00147752	0.04087323
Cep290	-0.5785754	0.001496928	0.04127754
5730507C01Rik	0.79493721	0.001499549	0.04127754
Rpn1	0.70413875	0.001500895	0.04127754
Bcl2a1d	1.22905521	0.001509242	0.04142649
Edem1	0.72181631	0.001520398	0.04165182
P2ry6	1.27689499	0.001528283	0.04178686
Manf	1.5425311	0.001545671	0.04218072
Retnla	4.45354706	0.001551678	0.04218494
Atp6v1b2	0.48599242	0.001551795	0.04218494
Sirpa	0.61869959	0.001554986	0.04219056
Adra1d	-1.4130316	0.001566278	0.04240433
Serpina3n	2.47479282	0.001570781	0.04240433
Glipr2	1.53223931	0.001571864	0.04240433
Gm10033	-0.6583865	0.001583422	0.04256581
Mdm1	-0.5722108	0.001585052	0.04256581
Dcaf6	-0.6375818	0.001586883	0.04256581
Fbln2	-1.73733	0.001590019	0.04256914
Slc4a2	0.52092397	0.001596702	0.04257286
Spring1	0.5724542	0.00159867	0.04257286
Cyba	1.24164646	0.001600014	0.04257286
Akr1b7	-1.3054386	0.001602204	0.04257286
Ttll13	-1.1635316	0.001617146	0.04288925
Rab15	0.70492775	0.001626306	0.04301388
Slamf1	1.55536428	0.00162793	0.04301388
Ccr7	1.33987024	0.001632744	0.04306058
Flt3	1.40057652	0.001635947	0.0430647
Calr	0.74572869	0.001652852	0.04342884
Ankra2	-0.5031133	0.001661773	0.04356504
Icam1	0.680906	0.001666126	0.04356504
Nuak2	0.63708508	0.001672059	0.04356504
Enpp6	-1.2438257	0.001672701	0.04356504
Pdk4	-2.391246	0.001673445	0.04356504
Sirpb1a	1.25673748	0.001683788	0.04364724
Angptl2	-0.9644544	0.001685364	0.04364724
Hpx	1.41256818	0.001687131	0.04364724
Ampd2	0.55631515	0.001691866	0.04364724
Cd177	3.67819845	0.001693262	0.04364724
Acad10	-0.5828278	0.001695128	0.04364724
Chmp1b	-0.4273047	0.001714632	0.04406917
Mttp	-0.6988034	0.00172112	0.04415562
Yipf1	0.55288167	0.001725255	0.0441618
Dpyd	-1.6053879	0.001727609	0.0441618
Grap2	0.95597386	0.001741723	0.04437985
Cd14	1.61344686	0.001742418	0.04437985
Tbc1d12	-0.7835383	0.001757223	0.04467644
Acvr2b	-0.7602012	0.001764049	0.04476947
Vpreb1	-0.7478685	0.00176862	0.04480505
2700081O15Rik	-0.7500215	0.001786356	0.0451734
Copz1	0.79855713	0.001799659	0.04542855
Pcdhga4	-0.7856594	0.001820829	0.04580556
Padi2	0.77199258	0.001821076	0.04580556
Foxq1	-1.0908928	0.001835982	0.04609847
5730455P16Rik	-0.6161245	0.001847885	0.04627621
Dennd1c	0.67643321	0.001853665	0.04627621
Rasal3	1.13666755	0.001856815	0.04627621
Gpr85	1.30085831	0.001860583	0.04627621
Otulinl	0.61120289	0.001860969	0.04627621
Zc3h12a	1.29746654	0.001864722	0.04627621
N4bp1	0.48612338	0.001865976	0.04627621
Zfp942	-0.6112588	0.001877552	0.04640564
H2-Ab1	0.95226063	0.001877761	0.04640564
Ktn1	-0.5639551	0.00188225	0.04643541
2810459M11Rik	-1.5430298	0.001893595	0.04660698
Dhx58	0.92234681	0.001895838	0.04660698
Pcsk6	-1.7618772	0.001900966	0.04660698
Ebi3	1.26772259	0.001902393	0.04660698
Trim45	-0.7065587	0.001910528	0.04669584
Orai1	0.86906039	0.001917398	0.04669584
Zfp267	-0.8017584	0.001917456	0.04669584
Ptprc	0.71347696	0.001919233	0.04669584
Tm4sf5	1.68426641	0.001942952	0.0471096
Fcgr1	1.43331948	0.001944902	0.0471096
Gpr132	1.05748775	0.001949002	0.0471096
Gabrr2	-1.6650453	0.001949918	0.0471096
Traf3ip3	0.79025706	0.001952948	0.0471096
Anxa4	0.46244915	0.001956353	0.0471096
Pnpo	0.56993664	0.001959568	0.0471096
Rhog	0.61691715	0.001967218	0.04721323
Ppp1cb	-0.4574567	0.001978293	0.04739857
Klhdc1	-0.8945389	0.001990106	0.04752569

Sit1	1.55935357	0.001990323	0.04752569
Rftn1	0.67874961	0.001997501	0.04761665
Wfdc13	2.15181887	0.002011756	0.04787572
B3galt4	0.72928449	0.002017133	0.04788824
Trarg1	-0.8481154	0.002019057	0.04788824
Rassf9	-0.6463825	0.002032839	0.04805896
Zfp960	-0.9641684	0.002034667	0.04805896
Lrmda	0.82782341	0.002036692	0.04805896
Esyt3	-1.2399102	0.002041438	0.04805896
Arpc1b	0.94711017	0.002044454	0.04805896
P2ry2	1.24798011	0.002046653	0.04805896
Macrod1	-0.9111345	0.00206283	0.04835847
Il18bp	1.22720045	0.002078398	0.04857958
Ubash3b	0.71349657	0.002079134	0.04857958
Azgp1	-2.0927594	0.002089738	0.04871715
Zbtb10	-0.5653794	0.00209222	0.04871715
Gadd45g	1.68329605	0.002095361	0.04871715
Kcnj8	-0.7032395	0.002121553	0.04924512
Plekho2	0.90776776	0.002125351	0.04925239
Peg3	-0.8247547	0.002134791	0.04939018
Ppp2r3a	-1.0891248	0.002157418	0.04983212
Zmynd15	0.8030713	0.002163085	0.04988151
Prmt8	0.88047215	0.002170225	0.04996466

Table.S11
GSEA Pathways by CRA vs vehicle

ID	Description	Enrichment Score	NES	Unadjusted P	Adjusted P	Core Enrichment Genes
WP2292	Chemokine signaling pathway	0.621876537	2.47797175	1.00E-10	1.53E-08	Rac2/Ccl8/Cxcl2/Ccl24/Fgr/Prkcb/Ccr3/Ccl17/Ccl11/Ccl19/Ccl3/Vav1/Cxcl5/Cxcr6/Ccr8/Ccr5/Ccl4/Ccr2/Ccl12/Ccl6/Jak3/Prkcd/Ccr1/Ccl20/Ccl7/Cxcr2/Ncf1/Ccl9/Ccl22/Ccr7/Cxcl9/Pik3r5/Cxcl1/Ccr6/Ccl1/Cxcl10/Arrb2/Cxcl16/Pik3cd/Nfkbib/Cxcl15/Ccr4/Itk/Nfkbia/Csk/Nfkb1/Ccl2/Cxcl12/Grk6/Cxcl13/Cxcr5/Pf4/Ccl5
WP163	Cytoplasmic ribosomal proteins	0.673084117	2.41298978	2.61E-10	2.00E-08	Rps6kb2/Rpl13a/Rpl18/Rpl35a/Rps3/Rpl38/Rpl35/Rps11/Rpl10a/Rpsa/Rpl28/Rpl27a/Rps15a/Rps27a/Rpl8/Rpl34/Rpl34/Rps18/Rps15/Rpl41/Rplp2/Rpl7a/Rpl37a/Rps2/Rps13/Rps17/Rps7/Rpl17/Rps29/Rps16/Rpl27/Rpl27/Rps8/Rps8/Rps25/Rpl9/Rps26/Rpl19/Rps6/Rps3a1/Rpl26/Rpl12/Rplp1/Rps12/Fau/Rpl23a/Rpl11/Rpl6/Rplp0/Rpl29/Rpl13/Rpl7/Rps21/Rpl21/Rps5/Rpl15/Rpl36/Rpl37/Rpl37/Rps19/Rps27/Rpl3/Rps24/Rpl30/Rps23/Rps14/Rpl18a/Rpl31/Rpl32/Rpl22/Rps10/Rpl4
WP3625	Tyrobp causal network in microglia	0.713606619	2.42442286	4.00E-10	2.04E-08	Gapt/C3/Samsn1/Ncf2/Hcls1/Cd84/Nrros/Tmem106a/Cd37/Cd4/Itgax/Ppp1r18/Rgs1/Tyrobp/Cxcl16/Apbb1ip/Tcirg1/Itgb2/Bin2/Gpx1/Nckap1l/Tnfrsf1b/C1qc/Npc2/Slc7a7/Capg/Creb3l2/Runx3/Igsf6
WP3626	Microglia pathogen phagocytosis pathway	0.731235998	2.32510033	2.17E-08	8.30E-07	Lat/Rac2/Ncf2/Vav1/Ncf4/Ptpn6/Fcer1g/Ncf1/Cyba/Fcgr1/C1qb/Syk/Arpc1b/Tyrobp/Itgb2/C1qa/Pik3cd/Trem3/Nckap1l/Trem2/C1qc/Siglece
WP1253	Type II interferon signaling (IFNG)	0.74474137	2.22544108	2.38E-07	7.27E-06	Il1b/Socs1/Spi1/Irf4/Prkcd/Irf8/Cxcl9/Psmb9/Cxcl10/Isg15/Ifngr2/Icam1/Eif2ak2/Socs3/Irf1/Tap1/Irf2/Irf9
WP189	GPCRs class A rhodopsin-like	0.557108708	2.05993141	1.13E-06	2.88E-05	Ptgdr/Htr7/Ccr3/Gpr65/Ccr8/Ccr5/Ccr2/Ccr1/Ptafr/Cxcr2/Adora3/Adora3/Gpr35/Ptgir/Hrh2/P2ry6/Ccr6/Adora2b/Adra2a/Adora2a/Ccr4/Ltb4r1/C3ar1/Ptger4/P2ry2/Cxcr5/Bdkrb1/Adra1b
WP373	IL-3 signaling pathway	0.537670519	2.008287	1.90E-06	4.15E-05	Fcer2a/Lck/Rac2/Matk/Prkcb/Spi1/Vav1/Ptpn6/Csf2rb/Cish/Syk/Selp/Rps6kb2/Pik3cd/Cbl/Tnfrsf1b/Nfkb1/Socs3/Mras/Mmp9/Socs2/Fes/Grb2/Fyn/Il3ra/Rack1/Shc1/Bcl2l1/Rara/Bax/Cdc42/Bad/Hck/Ywhaz/Akt1/Bcl2/Ywhab/Inpp5d/Stat1
WP222	Cytokines and inflammatory response	0.781904853	2.17727059	2.62E-06	5.01E-05	Il10/Cxcl3/Il1b/Tnf/Cd4/Il4/Cxcl1/H2-Eb1/Il5/Il6/Csf2
WP519	Proteasome degradation	0.634338611	2.0898164	3.88E-06	6.59E-05	Psmb8/Psmb10/Psme1/Psmb9/Rpn1/Psme2/Rpn2/Psmb5/Psmd9/Psmb2/Psma6/Psmb6/H2az1/Psma2/Psma7/Psma5/Psmd13/Psma4/Psmb4/Psmc3/H2ax/Psma3/Psmb1/Psme3/Psmc5/Ube2d2a/H2-Q10/Psmc6/Psmd6/Psmd8/Psmc4/Psmd4/Psmb7
WP151	IL-5 signaling pathway	0.575794269	2.02344246	5.06E-06	7.74E-05	Socs1/Prkcb/Hcls1/Vav1/Ptpn6/Csf2rb/Prkcd/Il2rb/Pim1/Syk/Il5/Alox5ap/Itgb2/Icam1/Cbl/Nfkbia/Ccnd3/Sh2b2/Nfkb1
WP3632	Lung fibrosis	0.595512459	2.04104942	6.37E-06	8.87E-05	Il1b/Cxcl2/Ccr3/Ccl11/Ccl3/Ccl4/Ccr2/Tnf/Il4/Il5/Il6/Sftpa1/Csf2/Cxcl15/Ccl2/Calca/Mmp9/Tgfb1/Ccl5/Cebpb/Igf1
WP1269	Fatty acid beta-oxidation	-0.672377572	-2.2058093	7.72E-06	9.85E-05	Crat/Echs1/Lipe/Hadhb/Acsl3/Hadha/Lpl/Cpt2/Hadh/Pnpla2/Dld/Acsl1/Cpt1a/Acss2/Acadm/Lipf
WP310	mRNA processing	0.374185021	1.6218416	9.29E-06	0.00010627	Oas1g/Oas1a/Cyp4f18/Oas3/Rbm14/Zbp1/Oasl2/Fbl/Cfb/Hnrnpc/Hnrnpa3/Eif2ak2/Alyref2/Rnpep/Srsf9/Imp4/Snrnp35/Oas2/Tmed10/Polr2g/Snrpd3/Slbp/Nop9/Hnrnpf/U2af2/Mrpl23/Hnrnpab/Raly/Elavl1/Lsm4/Snrpg/Sf3b5/Mrps28/Sf3b6/Srp19/Eif4e/Nudt21/Snrnp40/Snrpb/Lsm7/Mrpl11/Naa38/Snrpa1/Sfpq/Exosc8/Snrpf/Secisbp2/Snrpd1/Pcolce/Alyref/Srp9/Rpl38/Nip7/Ptbp1/Sf3a1/Snrpb2/Rbm4/Cpsf2/Lsm1/Pcbp2/Cdk9/Rae1/Rps11/Sf3b4/Ppie/Hnrnpa2b1/Rbm17/Hnrnpm/Rps9/Srp14/Clp1/Ppp1r8/Eif3g/Rbm8a/Snrpa/Exosc4/Ppp1r14b/Wdr55/Ddx39b/Zmat2/Rbm25/Rnaset2b/Snrpd2/Cstf3/Ssb/Rpl8/Dnd1/Lsm2/Tarbp2/Snrpe/Tial1/Ltv1/Dhx9/Rps13/Srsf6/Eif4a3/Prmt1/Rps7/Pcbp1/Rps29/Hnrnpa0/Lsm8/Rpl9/Mrm3/Npm1/Rpl19/Phf5a/Ddx21/Ybx1/Park7/Snrpc/Srsf3/Rps6/Adar/Rnaseh2a/Nifk/Rpl26/Rpl12/Sf3a2/Rbmxl1/Nufip1/Sf3a3/Tsn/Rnps1/Rbm47/Pum2/Mak16/Cpsf4/Rpl11/Tmem223/Nelfe/Srsf2/Tra2b/Hnrnpd/Snu13/Snu13/Hnrnpl/Syncrip/Rbm28/Exosc9/Brca1/Ddx56/Rpl7/Dazap1/G3bp1/Rpl37/Rpl37/Eif4e2/Srsf10/U2af1/Smn1/Eif4h/Rps27
WP1254	Apoptosis	0.556241467	2.01198421	9.83E-06	0.00010627	Irf4/Birc3/Nfkbie/Tnf/Lta/Irf5/Irf7/Tnfrsf25/Traf3/Nfkbib/Nfkbia/Tradd/Tnfrsf1b/Casp4/Nfkb1/Trp53/Traf2/Igf1/Casp6/Irf1/Casp1/Irf2/Casp8/Bcl2l1/Bak1/Fasl/Mcl1/Bax/Bad/Pmaip1/Rela/Fas/Akt1/Bcl2/Irf3/Casp3/Casp7/Myc
WP1396	GPCRs non-odorant	0.469671359	1.84547099	1.04E-05	0.00010627	Ptgdr/Htr7/Ccr3/Gpr65/Cxcr6/Ccr8/Ccr2/Ccr1/Ptafr/Gpr132/Cxcr2/Ffar2/Adora3/Adora3/Ccr7/Ptgir/Hrh2/P2ry6/Ccr6/Adora2b/Adra2a/Adora2a/Ccr4/Fzd5/Ltb4r1/C3ar1/Ackr1/Ptger4/Adgre1/P2ry2/Cxcr5/Bdkrb1/Gpr18/Adra1b/Lpar1
WP1248	Oxidative phosphorylation	0.609212491	2.00502656	3.79E-05	0.00036221	Gzmb/Ndufb5/Ndufa11/Atp5pb/Ndufa2/Ndufa3/Ndufs6/Atp5j2/Ndufb7/Atp5h/Ndufa7/Atp5g1/Ndufb6/Ndufs5/Atp5g2/Atp5g3/Ndufv2/Atp5d/Ndufb10/Ndufc2/Ndufs8/Atp5e/Atp5l/Ndufc1/Atp5k/Ndufs7/Atp5o/Ndufs4/Ndufb9/Ndufa6/Ndufa8/Ndufv3/Ndufs3/Ndufs2/Ndufa9/Atp5j
WP2271	Macrophage markers	0.869314229	1.99408624	4.08E-05	0.00036755	Rac2/Cd68/Cd52/Cd14/Cd86/Cd74/Cd83
WP450	IL-2 signaling pathway	0.527264974	1.90161712	5.72E-05	0.00048592	Lck/Socs1/Vav1/Ptpn6/Jak3/Cish/Il2rb/Cd53/Il2ra/Syk/Pik3cd/Icam1/Cbl/Nmi/Nfkb1/Socs3/Grb2/Eif4e/Fyn/Rack1/Shc1/Hsp90aa1/Pik3cg/Ptk2b/Rela/Map2k2/Ybx1/Akt1
WP5128	Fibrin complement receptor 3 signaling pathway	0.637282927	1.99821499	6.06E-05	0.0004879	Cxcl3/Fcer1g/Tnf/Syk/Cd14/Cxcl10/Tyrobp/Il6/Myd88/Itgb2/Nfkb1/Tlr4/Ticam2/Ccl2/Ticam1
WP2316	PPAR signaling pathway	-0.511835656	-1.9963437	7.39E-05	0.0005652	Ubc/Scd2/Slc27a2/Pparg/Cyp4a12b/Scd4/Ehhadh/Pdpk1/Acsl3/Fabp3/Scd1/Lpl/Sorbs1/Cpt2/Rxrg/Slc27a1/Ppara/Acsl1/Cpt1a/Rxra/Cyp27a1/Fabp1/Acox1/Acadm/Hmgcs2/Acaa1b
WP295	Electron transport chain	0.50737785	1.85020156	0.00013662	0.00099537	Ndufb5/Cox6a1/Cox8a/Uqcrh/Atp5pb/Ndufa2/Cox5b/Ndufa3/Ndufs6/Uqcrq/Atp5j2/Ndufb3/Ucp2/Ndufb7/Atp5h/Sdhd/Atpif1/Ndufa7/Cox6b1/Ndufb6/Ndufs5/Sdhb/Atp5g2/Atp5g3/Ndufv2/Atp5d/Ndufb10/Ndufc2/Cox5a/Ndufs8/Atp5e/Atp5l/Sdhc/Uqcr11/Atp5c1/Ndufc1/Ndufb4/Ndufb4/Ndufb4/Ndufa12/Cox4i1/Atp5k/Ndufs7/Atp5o/Ndufs4/Uqcrb/Ndufb9/Ndufa6/Ndufa8/Uqcr10/Ndufv3/Cox7a2/Ndufs3/Cox7a2l/Cox6c/Ndufs2/Ndufa9/Atp5j/Uqcrc2
WP458	Inflammatory response pathway	0.676309098	1.99208094	0.000175129	0.00118005	Lck/Cd28/Zap70/Il2rb/Il2ra/Il4/Il5/Cd40/Cd86/Tnfrsf1b/Thbs1
WP246	TNF-alpha NF-kB signaling pathway	0.413170017	1.65201179	0.000177393	0.00118005	Nfkbiz/Nfkb2/Tnfrsf8/Birc3/Nfkbie/Tnf/Relb/Traf3/Fbl/Actl6a/Nfkbib/Tnip1/Bcl3/Nfkbia/Tradd/Tnfrsf1b/Tab2/Polr2h/Nfkb1/Tank/Tnfaip3/Psmb5/Traf2/Ywhah/Ikbke/Polr1c/Polr1e/Ripk3/Smarcb1/Rps11/Dap/Rack1/Tnfrsf11a/Casp8/Map3k8/Psmd13/Hsp90aa1/Psmc3/Rpl8/Mcm5/Hdac1/Pebp1/Pfdn2/Rel/Rps13/Tifa/Eif4a3/Rela/Csnk2a1/Kpna6/Ywhaz/Akt1/Smarce1/Alpl/Ywhae/Psmd6/Csnk2a2/Ywhab/Stat1/Map3k14/Rpl6/Casp3/Casp7/Commd1/Mcm7/Fkbp5/Kpna2/Sumo1/Hsp90ab1/Psmc2/Ywhag/Nkiras2/Cradd/Polr1d/Rpl30
WP234	GPCRs peptide	0.632194707	1.91853869	0.000188841	0.00120386	Ccr3/Cxcr6/Ccr8/Ccr5/Ccr2/Ccr1/Cxcr2/Ccr7/Ccr6/Ccr4/C3ar1/Cxcr5/Bdkrb1
WP1496	Oxidative damage response	0.584153927	1.85742564	0.000475016	0.00290709	Nfkbie/Tnf/C1qb/C1ra/C1qa/Traf3/Tnfrsf1b/C1qc/C3ar1/Nfkb1/Traf2/Bak1/Cycs/Pcna/Cdc42/Bad/Bcl2/C2/Casp3/Gadd45a/C4b/C5ar1/Cdkn1a
WP150	DNA replication	0.582264493	1.85141783	0.000542393	0.00305796	Pola2/Mcm3/Rfc2/Uba52/Pold2/Cdc6/Pold4/Cdt1/Cdc45/Rpa2/Pole2/Mcm2/Mcm5/Pold1/Rfc4/Pcna/Rfc3/Rpa3/Orc1/Rfc5/Cdc7/Mcm6/Pold3/Mcm7/Mcm10/Prim2/Mcm4/Cdk2/Orc6/Pole
WP2432	Spinal cord injury	0.468325741	1.72854567	0.000547737	0.00305796	Fcgr2b/Ltb/Tnfsf13b/Il1b/Cxcl2/Mmp12/Ccr2/Tnf/Il4/Cxcl1/Cxcl10/Il6/Selp/Pirb/Arg1/Icam1/Tnfsf13/Ltb4r1/Tlr4/Ccl2/Mmp9/Trp53/Tgfb1
WP412	Oxidative stress response	0.65187166	1.92010002	0.000559627	0.00305796	Cyba/Gclc/Sod3/Gpx1/Nfkb1/Junb/Txn2/Sod2/Gpx3/Gabpb1/Gsr/Txnrd2/Fos/Hmox1/Gstt2
WP200	Complement activation classical pathway	0.753570526	1.85572003	0.000734267	0.00387389	C3/C1qb/C1ra/C1qa/C1qc/C7
WP336	Fatty acid biosynthesis	-0.644641355	-1.9709045	0.001207564	0.00602708	Ech1/Echdc2/Echs1/Acsl3/Fasn/Scd1/Acly/Acaca/Hadh/Acsl1/Acacb/Echdc3/Acaa2/Acss2
WP4466	Oxidative stress and redox pathway	0.463154454	1.68163942	0.001221173	0.00602708	Ctla4/Ncf2/Ncf4/Ggt5/Ncf1/Cyba/Ptgs1/Gclc/S100a8/Cd44/Lap3/S100a9/Sod3/Gpx1/Slc7a11/Sod2/Gpx3/Gsr
WP4347	Eicosanoid metabolism via cyclooxygenases (COX	0.634146469	1.84363321	0.001503928	0.00719066	Ptgdr/Tbxas1/Cyp4f18/Ptgir/Ptgs1/Ptges
WP339	ESC pluripotency pathways	-0.373146911	-1.6319799	0.00162771	0.00754665	Smad5/Lifr/Fgfr3/Axin1/Bmpr1b/Fgf2/Fgf12/Wnt11/Fgfr1/Wnt3a/Fzd8/Lrp5/Fzd3/Lrp6/Gab1/Wnt4/Fzd7/Fgf7/Raf1/Fgfr2/Wnt7b/Wnt7a/Egfr/Pdgfrb/Smad9/Selenop/Fgf1/Smad4/Fzd2/Egf/Pdgfra/Map2k3/Mtor/Wnt2b/Pik3r2/Fzd1/Acvr1/Wnt10b/Dvl1/Map2k6/Mapk6/Fzd6/Bmpr1a/Mapk12
WP454	Osteoclast signaling	0.750655959	1.81530173	0.001796524	0.00808436	Ctsk/Tnfsf11/Acp5/Atp6v1g1/Gpr68/Tnfrsf11a
WP374	Prostaglandin synthesis and regulation	0.586642413	1.75353143	0.002133757	0.00932756	Ptgdr/Tbxas1/Ptgir/Ptgs1/Ptger4/Hsd11b2/Anxa4
WP1272	Selenium micronutrient network	0.660645052	1.82038084	0.002461914	0.01046313	Tbxas1/Kmo/Ptges/Gpx1/Selenok/Gpx3/Pnpo/Gsr
WP2318	Fatty acid oxidation	-0.731382684	-1.7933071	0.002839582	0.01174205	Echs1/Cpt2/Mttp/Hadh/Cpt1a/Acaa2/Acadm
WP460	Blood clotting cascade	0.77449345	1.77658053	0.003227156	0.01299355	F10/F7/Vwf
WP567	Eukaryotic transcription initiation	0.534498481	1.69953695	0.003747291	0.01470091	Taf12/Polr2h/Polr2g/Taf9/Taf9/Polr1c/Gtf2f2/Polr2j/Polr3k/Gtf2e2/Gtf2h3/Polr2k/Gtf2b/Polr2e/Gtf2h2/Ilk/Polr2i/Taf13/Polr1d/Cdk7/Polr2c/Gtf2h4/Polr3d/Taf5/Tbp
WP488	Alpha 6 beta 4 integrin signaling pathway	-0.403369401	-1.6237468	0.004032886	0.01542579	Irs1/Lama5/Lamc1/Yes1/Pik3ca/Ptk2/Egfr/Col17a1/Erbin/Dsp/Mtor/Lama3/Dst/Erbb2/Rtkn/Itga6/Pik3r3/Lamb2/Abl1/Lamb1/Pik3r2/Pik3cb/Itgb4/Smad3/Lama2/Lamb3/Ntn1
WP2573	Primary focal segmental glomerulosclerosis (FSG	-0.390876805	-1.5892644	0.00615304	0.02296134	Smarcal1/Dnm1/Itgb3/Agrn/Cdkn1b/Mme/Notch1/Trpc6/Lrp5/Synpo/Tln1/Myh9/Itgb1/Lrp6/Vtn/Plcg1/Lama5/Itgav/Ptk2/Podxl/Cdk5rap2/Utrn/Col4a3/Vcl/Dag1/Cdh2/Lamb2/Jag1/Irf6/Plce1/Parva/Actn4/Itgb4
WP1983	Splicing factor NOVA regulated synaptic proteins	-0.480872294	-1.6705393	0.006526287	0.02362804	Epb41l2/Plcb4/Aplp2/Map4/Clstn1/Cdh2/Clasp1/Ank3/Neo1/Rap1gap/Epb41l3/Epb41l1
WP1243	EBV LMP1 signaling	0.640297152	1.7305122	0.006750083	0.02362804	Nfkb2/Ccl20/Tnf/Tradd/Nfkb1/Ccl5
WP317	Glycogen metabolism	-0.496946343	-1.7091834	0.006794992	0.02362804	Ppp2r2c/Ppp2r1b/Pygl/Gys1/Ppp2r2a/Phkg1/Phkb/Agl/Pygm/Gbe1/Ppp2r3a/Ppp2r2b
WP509	Nuclear Receptors	-0.492693093	-1.6945549	0.007521161	0.02557195	Nr4a2/Rarg/Pparg/Rarb/Esr2/Nr2f2/Nr3c1/Rxrg/Thrb/Nr2c2/Ppara/Esr1/Rxra/Rora/Thra/Nr1d2
WP3857	Wnt signaling in kidney disease	-0.456392107	-1.64151	0.008175902	0.02719376	Mapk9/Axin1/Wnt11/Mapk10/Wnt3a/Fzd8/Mapk8/Lrp5/Fzd3/Kitl/Invs/Wnt4/Fzd7/Wnt7b/Btrc/Wnt7a/Fzd2/Wnt2b/Fzd1/Wnt10b/Dvl1/Fzd6
WP386	Triacylglyceride synthesis	-0.601045542	-1.7989838	0.008450659	0.02750959	Lipe/Gpam/Lpl/Pnpla2/Gnpat/Dgat2/Agps/Lipf
WP413	G1 to S cell cycle control	0.458548658	1.58383259	0.009356196	0.02982287	Pola2/Ccnd3/Trp53/Mcm3/Ccna1/Cdkn2d/E2f4/Cdk4/Cdk6/Cdc45/Rpa2/Pole2/Mcm2/Ccnd2/Mcm5/Pcna/Cdc25a/Rpa3/Pkmyt1/Orc1/Ccnd1/E2f2/Mcm6/Myc/Mcm7/Gadd45a/Ccne1/Prim2/Mcm4/Cdk7/Cdkn1a/Cdk1/Cdk2/Orc6/Pole/E2f1
WP41	GPCRs other	0.540907112	1.66532675	0.010462206	0.0326677	Gpr84/Gpr132/Adora3/Adora3/Ccr7/Fzd5/Ackr1/Gpr88
WP3652	EDA signaling in hair follicle development	0.656959823	1.61780943	0.011312679	0.03402086	Ltb/Nfkb2/Relb/Nfkb1/Shh/Ascc1
WP407	Kit receptor signaling pathway	0.440206256	1.54191649	0.011340287	0.03402086	Matk/Fgr/Socs1/Prkcb/Vav1/Ptpn6/Csf2rb/Cish/Cbl/Sh2b2/Fes/Grb2/Fyn/Mitf/Shc1/Dok1/Pik3cg/Bad/Socs4/Hck/Akt1/Stap1/Inpp5d/Stat1

Table.S12
GSEA Pathways by 3EH+CRA vs vehicle

ID	Description	Enrichment Score	NES	Unadjusted P	Adjusted P	Core Enrichment Genes
WP2292	Chemokine signaling pathway	0.63157629	2.39151659	1.00E-10	7.65E-09	Fgr/Rac2/Cxcl2/Vav1/Cxcr6/Ccl8/Prkcb/Ncf1/Ccl24/Ccr3/Cxcl5/Ccr1/Cxcr2/Ccl3/Prkcd/Ccl6/Ccr2/Ccl11/Ccr5/Jak3/Ccl19/Ccl12/Ccl17/Ccl4/Pik3cd/Ccl7/Ccr7/Ccr8/Ccl9/Ptk2b/Cxcl10/Pik3r5/Ccr6/Itk/Cxcl16/Cxcl1/Cxcl12/Ccl22/Ccr4/Cxcl15/Nfkbib/Arrb2/Ccl20/Cxcr5/Jak2/Grk6/Ccl1/Dock2/Nfkbia/Pik3cg/Cxcl9/Shc1/Ccl2/Hck
WP3625	Tyrobp causal network in microglia	0.770964264	2.50049406	1.00E-10	7.65E-09	Ncf2/C3/Cd84/Hcls1/Gapt/Samsn1/Itgb2/Nrros/Cd37/Tmem106a/Itgax/Tnfrsf1b/Nckap1l/Igsf6/Tyrobp/Ppp1r18/Cd4/Cxcl16/Il10ra/Tcirg1/Capg/Bin2/Plek/Gpx1/Npc2/Lhfpl2/Slc7a7/Apbb1ip/Creb3l2/C1qc/Rgs1
WP3626	Microglia pathogen phagocytosis pathway	0.783463181	2.42292175	3.72E-10	1.90E-08	Lat/Rac2/Ncf2/Vav1/Ncf1/Ptpn6/Ncf4/Fcer1g/Itgb2/Nckap1l/Pik3cd/Siglece/Trem1/Syk/Cyba/Fcgr1/Arpc1b/Tyrobp/Trem3/C1qb/Trem2/C1qa/Pik3cg/C1qc/Hck
WP1253	Type II interferon signaling (IFNG)	0.784392648	2.29076822	1.32E-08	5.04E-07	Il1b/Spi1/Socs1/Irf4/Prkcd/Irf8/Icam1/Cxcl10/Ifngr2/Eif2ak2/Nos2/Socs3/Psmb9/Jak2/Isg15/Cxcl9
WP151	IL-5 signaling pathway	0.653692719	2.18499311	5.82E-08	1.78E-06	Csf2rb/Vav1/Prkcb/Ptpn6/Hcls1/Socs1/Prkcd/Itgb2/Alox5ap/Il2rb/Pim1/Syk/Icam1/Ptk2b/Sh2b2/Il5/Cbl/Jak2/Ccnd3/Nfkbia/Pik3cg/Shc1/Hck/Unc119/Nfkb1/Akt1/Il5ra/Stat5a/Map2k2/Itgam/Sdcbp/Stat3
WP5128	Fibrin complement receptor 3 signaling pathway	0.710856967	2.17100123	6.74E-07	1.72E-05	Cxcl3/Fcer1g/Itgb2/Tnf/Syk/Cd14/Tyrobp/Myd88/Cxcl10/Il6/Ticam2/Nos2/Ccl2/Ticam1/Rassf5/Tlr4/Rel/Irak2/Nfkb1/Akt1/Lbp/Irf3
WP373	IL-3 signaling pathway	0.556485937	1.95432192	1.97E-06	4.30E-05	Fcer2a/Lck/Rac2/Spi1/Matk/Csf2rb/Vav1/Prkcb/Ptpn6/Mmp9/Tnfrsf1b/Pik3cd/Syk/Cish/Fes/Selp/Rps6kb2/Cbl/Socs3/Jak2/Shc1/Hck/Rack1/Socs2/Nfkb1/Akt1/Stat5a
WP519	Proteasome degradation	0.652258237	2.076953	3.74E-06	7.16E-05	Psmb8/Rpn1/Rpn2/Psme1/Psmb10/Psme2/Psmb9/Psmd9/Psma6/H2az1/Psmb2/Psmb5/Psme3/Psmd2/H2-Q10/Psmd13/Psma5/Psmb6/Psmc3/Psmb4/Psma4/Psmd4/Psmc4/Psma3/Psma2/Psmd6/Psmc5/Psmd8/Psmb1/Psma7/Psmc2/Psmd3/Psmd11
WP450	IL-2 signaling pathway	0.578119235	1.96644457	4.53E-06	7.69E-05	Lck/Vav1/Ptpn6/Socs1/Cd53/Jak3/Pik3cd/Il2rb/Syk/Il2ra/Icam1/Ptk2b/Cish/Cbl/Socs3/Jak2/Pik3cg/Nmi/Shc1/Eif3b/Rack1/Nfkb1/Akt1/Stat5a/Fyn/Map2k2
WP189	GPCRs class A rhodopsin-like	0.557103519	1.95159843	6.06E-06	9.27E-05	Ptgdr/Htr7/Ptafr/Ccr3/Ccr1/Cxcr2/Ccr2/Ptgir/Ccr5/Gpr65/Gpr35/Hrh2/Ltb4r1/Adora3/Adora3/Adora2b/P2ry6/P2ry2/Ccr8/Adora2a/Ccr6/Adra2a/C3ar1/Ccr4/Cxcr5/Ptger4/Bdkrb1/Adra1b/F2rl2/Ptger2/P2ry13/P2ry14/P2ry14
WP163	Cytoplasmic ribosomal proteins	0.581691185	1.96778507	1.08E-05	0.00015049	Rps6kb2/Rpl18/Rps3/Rps2/Rpsa/Rpl3/Rps3a1/Rpl8/Rps11/Rpl4/Rplp0/Rpl7a/Rps6/Rpl6/Rps25/Rps18/Rpl27a/Rpl17/Rpl35a/Rpl10a/Rps13/Rpl38/Rpl12/Rpl27/Rpl27/Rpl29/Rpl19/Rps7/Rps15a/Rpl35/Rps15/Rpl13/Rps8/Rps8/Rps27a/Rpl15/Rpl28/Rpl9/Rps5/Rps16/Rpl7/Rplp2/Rps19/Rpl34/Rpl18a/Rpl23a/Fau/Rpl26/Rps26/Rpl21/Rplp1/Rps12/Rps17/Rpl41/Rps10/Rpl37a/Rpl11/Rps24/Rpl32/Rpl30/Rps20/Rps14/Rpl13a
WP1396	GPCRs non-odorant	0.478878167	1.79147624	2.15E-05	0.00027432	Ptgdr/Htr7/Cxcr6/Ptafr/Ccr3/Ccr1/Cxcr2/Ccr2/Ptgir/Gpr65/Ffar2/Hrh2/Ltb4r1/Adora3/Adora3/Adora2b/P2ry6/Ccr7/Gpr132/P2ry2/Ccr8/Adgre1/Adora2a/Ccr6/Adra2a/Fzd5/C3ar1/Ccr4/Cxcr5/Gpr68/Ptger4/Bdkrb1/Gpr18/Adra1b/Ackr1
WP2271	Macrophage markers	0.875449723	1.94837318	3.81E-05	0.00044888	Rac2/Cd68/Cd52/Cd14/Cd86/Cd74/Cd83
WP1254	Apoptosis	0.547310176	1.86992646	5.54E-05	0.00060586	Irf4/Birc3/Tnf/Irf5/Nfkbie/Tnfrsf25/Tnfrsf1b/Lta/Traf3/Traf2/Irf7/Tradd/Nfkbib/Casp4/Nfkbia/Trp53/Bid/Nfkb1/Akt1/Mcl1/Irf3/Prf1/Irf1/Casp6/Casp1/Myc/Igf1/Casp8/Bak1
WP222	Cytokines and inflammatory response	0.749431886	2.01688405	8.94E-05	0.00091168	Il10/Cxcl3/Il1b/Tnf/Cd4/Cxcl1/Il6/H2-Eb1/Csf2/Il4/Il5/Csf1
WP458	Inflammatory response pathway	0.6840699	1.92776692	0.000125955	0.00120445	Lck/Zap70/Tnfrsf1b/Cd28/Il2rb/Il2ra/Thbs1/Cd40/Il4/Cd86/Il5/Il4ra/Fn1/Il5ra/Cd80
WP234	GPCRs peptide	0.6536965	1.93015738	0.0001454	0.0013086	Cxcr6/Ccr3/Ccr1/Cxcr2/Ccr2/Ccr5/Ccr7/Ccr8/Ccr6/C3ar1/Ccr4/Cxcr5/Bdkrb1
WP246	TNF-alpha NF-kB signaling pathway	0.434009758	1.65162689	0.00029213	0.00248311	Nfkbiz/Nfkb2/Birc3/Tnf/Nfkbie/Tnfrsf1b/Relb/Tnfrsf8/Traf3/Traf2/Tnfaip3/Tradd/Nfkbib/Tnfrsf11a/Bcl3/Actl6a/Ikbke/Fbl/Tank/Nfkbia/Smarcb1/Tnip1/Alpl/Map3k14/Polr2h/Polr1c/Rel/Rack1/Ywhah/Hdac1/Polr1e/Psmb5/Nfkb1/Ripk3/Akt1/Psmd13/Psmc3/Tifa/Eif4a3/Smarca4/Rpl8/Rps11/Psmd6/Rpl4/Mcm5/Rpl6/Psmc2/Psmd3/Casp8/Pml/G3bp2/Rps13/Dap/Map3k8/Psmd1/Tnfrsf1a/Ywhab/Pebp1/Ywhaz/Casp7/Smarce1/Psmc1/Nkiras2/Casp3
WP3632	Lung fibrosis	0.550006862	1.80738333	0.000346326	0.00278884	Il1b/Cxcl2/Ccr3/Ccl3/Ccr2/Tnf/Ccl11/Mmp9/Ccl4/Sftpa1/Il6/Atp11a/Cxcl15/Csf2/Il4/Il5/Cebpb/Hmox1/Ccl2/Dpp9/Pdgfb/Tgfb1/Pdgfa
WP412	Oxidative stress response	0.659124613	1.85746899	0.000409184	0.00313026	Gclc/Cyba/Gsr/Sod2/Gpx1/Sod3/Hmox1/Junb/Txnrd2/Gabpb1/Txn2/Fos/Nfkb1/Txnrd1
WP2432	Spinal cord injury	0.50068873	1.74446639	0.000453745	0.00330586	Fcgr2b/Ltb/Il1b/Cxcl2/Tnfsf13b/Mmp12/Ccr2/Tnf/Pirb/Mmp9/Ltb4r1/Icam1/Cxcl10/Cxcl1/Mag/Il6/Selp/Arg1/Il4/Nos2/Xylt1/Ccl2/Cdk4/Trp53/Tlr4/Fos/Il1r1
WP460	Blood clotting cascade	0.828222804	1.84326644	0.000477834	0.00332312	F10/F7/Vwf
WP103	Cholesterol biosynthesis	0.758760108	1.90429165	0.00129405	0.00860825	Mvd/Hmgcr/Idi1/Sqle/Cyp51/Msmo1/Dhcr7/Hmgcs1/Fdft1/Pmvk/Sc5d/Mvk/Fdps/Lss
WP1269	Fatty acid beta-oxidation	-0.571495347	-1.8518457	0.001442888	0.00919841	Crat/Hadhb/Decr1/Lpl/Acadl/Lipf/Cpt2/Acsl1/Pnpla2/Acss2/Dld/Lipe/Cpt1a/Acadm
WP200	Complement activation classical pathway	0.760334761	1.85778714	0.001830566	0.01120306	C3/C1ra/C1qb/C1qa/C1qc/C4b/C7
WP2318	Fatty acid oxidation	-0.764071007	-1.8991651	0.002229043	0.01311706	Cpt2/Acaa2/Cpt1a/Acadm/Mttp
WP407	Kit receptor signaling pathway	0.498541438	1.66984492	0.002534275	0.01391964	Fgr/Matk/Csf2rb/Vav1/Prkcb/Ptpn6/Socs1/Cish/Sh2b2/Fes/Cbl/Jak2/Mitf/Pik3cg/Shc1/Hck
WP454	Osteoclast signaling	0.759533002	1.81932951	0.002547384	0.01391964	Ctsk/Acp5/Tnfrsf11a/Gpr68/Tnfsf11/Pdgfb/Atp6v1g1
WP1496	Oxidative damage response	0.557325387	1.72357277	0.003603574	0.01836413	Tnf/Nfkbie/Tnfrsf1b/C1ra/Traf3/Traf2/C1qb/C3ar1/C1qa/C1qc/Nfkb1/C4b/C5ar1/Bak1/Pcna/Cdc42/Bad/Gadd45a
WP509	Nuclear Receptors	-0.507800192	-1.6941867	0.003626235	0.01836413	Rarb/Rxrb/Nr2c2/Rarg/Esr1/Ppara/Nr2f1/Rxra/Thrb/Nr3c1/Nr1d2/Thra/Rora/Nr2f2
WP1272	Selenium micronutrient network	0.654782094	1.74229877	0.003720838	0.01836413	Tbxas1/Kmo/Ptges/Pnpo/Gsr/Gpx1
WP297	IL-7 signaling pathway	0.531742963	1.65804612	0.003894262	0.01861944	Blk/Jak3/Il7r/Ptk2b/Cbl/Shc1/Cdk4/Muc1/Akt1/Mcl1/Stat5a/Fyn/Map2k2/Irf1/Stat3/Grb2/Bad/Lyn
WP4466	Oxidative stress and redox pathway	0.471365909	1.61398579	0.004215185	0.01954313	Ctla4/Ncf2/Ncf1/Ncf4/Ptgs1/Ggt5/S100a8/S100a9/Cd44/Gclc/Cyba/Gsr/Sod2/Lap3/Gpx1/Sod3/Txnrd2/Slc7a11/Hpgds/Gstm4/Txnrd1/Txnrd3/Ggct/Prdx3/Gstt2/Prdx5
WP4347	Eicosanoid metabolism via cyclooxygenases (COX)	0.608885794	1.70478067	0.006319003	0.02843551	Ptgdr/Tbxas1/Cyp4f18/Ptgs1/Ptgir/Ptges/Akr1b3
WP374	Prostaglandin synthesis and regulation	0.574510433	1.65231923	0.007627146	0.03334152	Ptgdr/Tbxas1/Ptgs1/Ptgir/Anxa4/Anxa6/Ptger4/Hsd11b2
WP435	One-carbon metabolism	0.57939023	1.63277074	0.008186829	0.03479402	Ahcyl2/Mat1a/Mthfd2/Mthfd1l/Dnmt3a/Shmt1/Shmt2/Dhfr/Gart
WP37	IL-1 signaling pathway	0.549930502	1.64983077	0.009262912	0.03830339	Il1b/Sirpa/Myd88/Nfkbib/Il1rn/Nfkbia/Map3k14/Il1r1/Irak2/Nfkb1/Akt1/Il1r2/Map3k7/Irak3/Casp1/Irak4
WP339	ESC pluripotency pathways	-0.338133386	-1.4270037	0.010078204	0.04057803	Fgf2/Braf/Fgfr3/Mapk4/Wnt2b/Pik3r2/Gab1/Fzd3/Wnt4/Fzd2/Map2k3/Lifr/Smad4/Fgfr1/Smad7/Wnt7b/Egfr/Fgf12/Pten/Map2k6/Smad9/Fgf1/Pdgfra/Fzd1/Bmpr2/Fzd8/Egf/Lrp5/Dvl1/Selenop/Fzd6/Acvr1/Bmpr1a/Mapk12/Fgf7
WP2316	PPAR signaling pathway	-0.394198047	-1.5316768	0.011747706	0.04608715	Pltp/Slc27a1/Fabp3/Lpl/Rxrb/Acadl/Ppara/Rxra/Cpt2/Acsl1/Sorbs1/Cyp8b1/Cyp27a1/Acox1/Fabp1/Cpt1a/Acadm/Hmgcs2/Acaa1b
WP88	Toll-like receptor signaling	0.536070443	1.56951452	0.012826821	0.04906259	Nfkb2/Traf3/Myd88/Ticam2/Eif2ak2/Ikbke/Ticam1/Tlr4/Irak2/Nfkb1/Mal/Irf3/Irak3/Casp8/Irak4/Mxd3

Table.S13

PeakID (cmd=annotatePeaks.pl lost_sites_strict_43_051021.bed hg19)	Chr	Start	End	Strand	Annotation	Detailed Annotation	Distance to TSS	Nearest PromoterID	Entrez ID	Nearest Unigene	Nearest Refseq	Nearest Ensembl	Gene Name	Gene Alias	Gene Description	Gene Type
**43 regions that were present only in all vehicle-treated samples but absent in all 12,13-diHOME-treated samples**																
chr3.165660509.165661060	chr3	165660510	165661060	+	Intergenic	L1MB7|LINE|L1	-105525	NM_000055	590	Hs.420483	NM_000055	ENSG00000114200	BCHE	BCHED|CHE1|CHE2|E1	butyrylcholinesterase	protein-coding
chr1.237638947.237639504	chr1	237638948	237639504	+	intron (NM_001035, intron 17 of 104)	Tigger10|DNA|TcMar-Tigger	4807	NR_039626	100616141		NR_039626	ENSG00000266262	MIR4428	-	microRNA 4428	ncRNA
chr8.104871549.104871873	chr8	104871550	104871873	+	intron (NR_145711, intron 1 of 21)	LTR78B|LTR|ERV1	40295	NM_001348500	9699	Hs.655271	NM_014677	ENSG00000176406	RIMS2	OBOE|RAB3IP3|RIM2	regulating synaptic membrane exocytosis 2	protein-coding
chr5.80148536.80148899	chr5	80148537	80148899	+	intron (NM_002439, intron 20 of 23)	intron (NM_002439, intron 20 of 23)	-107790	NM_006909	5924	Hs.162129	NM_006909	ENSG00000113319	RASGRF2	GRF2|RAS-GRF2	Ras protein specific guanine nucleotide releasing factor 2	protein-coding
chr13.22152858.22153218	chr13	22152859	22153218	+	intron (NM_152726, intron 1 of 11)	intron (NM_152726, intron 1 of 11)	25317	NM_152726	221154	Hs.412103	NM_152726	ENSG00000165487	MICU2	1110008L20Rik|EFHA1	mitochondrial calcium uptake 2	protein-coding
chr4.138120076.138120549	chr4	138120077	138120549	+	non-coding (NR_134640, exon 2 of 2)	non-coding (NR_134640, exon 2 of 2)	5403	NR_134640	729307	Hs.552764	NR_134640		LINC02510	-	long intergenic non-protein coding RNA 2510	ncRNA
chr14.71631835.71632346	chr14	71631836	71632346	+	Intergenic	Intergenic	-155096	NM_001354288	26037	Hs.654657	NM_015556	ENSG00000197555	SIPA1L1	E6TP1|SPAR1	signal induced proliferation associated 1 like 1	protein-coding
chr14.61863959.61864390	chr14	61863960	61864390	+	intron (NM_006255, intron 2 of 13)	intron (NM_006255, intron 2 of 13)	76014	NM_006255	5583	Hs.333907	NM_006255	ENSG00000027075	PRKCH	PKC-L|PKCL|PRKCL|nPKC-eta	protein kinase C eta	protein-coding
chr14.31072699.31073004	chr14	31072700	31073004	+	intron (NM_017769, intron 10 of 14)	AluJr|SINE|Alu	-18608	NM_001283033	23256	Hs.369168	NM_016106	ENSG00000092108	SCFD1	C14orf163|RA410|SLY1|SLY1P|STXBP1L2	sec1 family domain containing 1	protein-coding
chr5.111691991.111692527	chr5	111691992	111692527	+	intron (NR_144931, intron 1 of 22)	ERVL-E-int|LTR|ERVL	62754	NR_144931	64097	Hs.584954	NM_022140	ENSG00000129595	EPB41L4A	EPB41L4|NBL4	erythrocyte membrane protein band 4.1 like 4A	protein-coding
chr2.82567750.82568020	chr2	82567751	82568020	+	Intergenic	Intergenic	-516042	NR_033423	1720	Hs.169235	NR_033423		DHFRP3	-	dihydrofolate reductase pseudogene 3	pseudo
chr11.48175551.48175903	chr11	48175552	48175903	+	intron (NM_002843, intron 19 of 24)	MIRb|SINE|MIR	57393	NR_036119	100423000		NR_036119	ENSG00000263693	MIR3161	-	microRNA 3161	ncRNA
chr6.142366312.142366843	chr6	142366313	142366843	+	Intergenic	Intergenic	43358	NM_002511	4829	Hs.552106	NM_002511	ENSG00000135577	NMBR	BB1|BB1R|NMB-R	neuromedin B receptor	protein-coding
chr11.110018392.110018804	chr11	110018393	110018804	+	intron (NM_033390, intron 2 of 5)	intron (NM_033390, intron 2 of 5)	54511	NM_033390	85463	Hs.376289	NM_033390	ENSG00000149289	ZC3H12C	MCPIP3	zinc finger CCCH-type containing 12C	protein-coding
chr12.102415489.102416159	chr12	102415490	102416159	+	intron (NM_016053, intron 6 of 6)	intron (NM_016053, intron 6 of 6)	40078	NM_001301107	51019	Hs.405692	NM_016053	ENSG00000120860	WASHC3	CCDC53|CGI-116	WASH complex subunit 3	protein-coding
chr12.22343736.22344296	chr12	22343737	22344296	+	Intergenic	LTR51|LTR|ERV1	143632	NM_001304450	6489	Hs.408614	NM_003034	ENSG00000111728	ST8SIA1	GD3S|SIAT8|SIAT8-A|SIAT8A|ST8SiaI	ST8 alpha-N-acetyl-neuraminide alpha-2,8-sialyltransferase 1	protein-coding
chr8.68914762.68915218	chr8	68914763	68915218	+	intron (NM_025170, intron 1 of 23)	intron (NM_025170, intron 1 of 23)	50640	NM_025170	80243	Hs.169943	NM_024870	ENSG00000046889	PREX2	DEP.2|DEPDC2|P-REX2|PPP1R129	phosphatidylinositol-3,4,5-trisphosphate dependent Rac exchange factor 2	protein-coding
chr12.88938632.88939039	chr12	88938633	88939039	+	intron (NM_000899, intron 2 of 9)	intron (NM_000899, intron 2 of 9)	35414	NM_003994	4254	Hs.1048	NM_000899	ENSG00000049130	KITLG	DCUA|DFNA69|FPH2|FPHH|KL-1|Kitl|MGF|SCF|SF|SHEP7|SLF	KIT ligand	protein-coding
chr20.7093153.7093514	chr20	7093154	7093514	+	Intergenic	Intergenic	141515	NR_110609	101929265	Hs.637730	NR_110609	ENSG00000228888	LINC01428	-	long intergenic non-protein coding RNA 1428	ncRNA
chr3.150233058.150233475	chr3	150233059	150233475	+	Intergenic	L1ME3|LINE|L1	31161	NM_014445	27230	Hs.518326	NM_014445	ENSG00000120742	SERP1	RAMP4	stress associated endoplasmic reticulum protein 1	protein-coding
chr4.120478049.120478328	chr4	120478050	120478328	+	intron (NM_001083, intron 7 of 20)	LTR16B2|LTR|ERVL	70253	NM_033430	8654	Hs.647971	NM_001083	ENSG00000138735	PDE5A	CGB-PDE|CN5A|PDE5	phosphodiesterase 5A	protein-coding
chr4.93609067.93609447	chr4	93609068	93609447	+	intron (NM_001286838, intron 2 of 14)	AluSq|SINE|Alu	383707	NM_001286838	2895	Hs.162727	NM_001510	ENSG00000152208	GRID2	GluD2|SCAR18	glutamate ionotropic receptor delta type subunit 2	protein-coding
chr15.84703817.84704331	chr15	84703818	84704331	+	intron (NM_001301110, intron 28 of 29)	intron (NM_001301110, intron 28 of 29)	-44865	NR_036652	648809	Hs.585157	NR_036652		EFL1P1	EFTUD1P1|FAM42B|HsT19321	elongation factor like GTPase 1 pseudogene 1	pseudo
chr6.147609115.147609485	chr6	147609116	147609485	+	intron (NM_139244, intron 8 of 25)	L1M4|LINE|L1	-83550	NR_034115	729178	Hs.557608	NR_034115		STXBP5-AS1	-	STXBP5 antisense RNA 1	ncRNA
chr8.104106284.104106719	chr8	104106285	104106719	+	Intergenic	Tigger1|DNA|TcMar-Tigger	-26758	NR_145429	379034	Hs.591862	NR_145429		LINC01181	-	long intergenic non-protein coding RNA 1181	ncRNA
chr18.22237028.22237382	chr18	22237029	22237382	+	intron (NR_040033, intron 1 of 2)	intron (NR_040033, intron 1 of 2)	29059	NR_040033	729950	Hs.585850	NR_040033	ENSG00000265485	LINC01915	-	long intergenic non-protein coding RNA 1915	ncRNA
chr18.37262417.37262763	chr18	37262418	37262763	+	intron (NR_024391, intron 2 of 3)	L2c|LINE|L2	-5849	NR_049848	100846997		NR_049848	ENSG00000283645	MIR5583-2	-	microRNA 5583-2	ncRNA
chr19.39152734.39153362	chr19	39152735	39153362	+	intron (NM_004924, intron 1 of 20)	intron (NM_004924, intron 1 of 20)	14792	NM_001322033	81	Hs.270291	NM_004924	ENSG00000130402	ACTN4	ACTININ-4|FSGS|FSGS1	actinin alpha 4	protein-coding
chr3.189978049.189978421	chr3	189978050	189978421	+	Intergenic	MLT1B|LTR|ERVL-MaLR	62000	NM_021101	9076	Hs.439060	NM_021101	ENSG00000163347	CLDN1	CLD1|ILVASC|SEMP1	claudin 1	protein-coding
chr14.36117382.36117915	chr14	36117383	36117915	+	intron (NM_014990, intron 30 of 40)	AluSx|SINE|Alu	114401	NM_032594	84684	Hs.62813	NM_032594	ENSG00000168348	INSM2	IA-6|IA6|mlt1	INSM transcriptional repressor 2	protein-coding
chr8.55379059.55379467	chr8	55379060	55379467	+	Intergenic	CpG	8768	NM_022454	64321	Hs.98367	NM_022454	ENSG00000164736	SOX17	VUR3	SRY-box transcription factor 17	protein-coding
chr9.12076040.12076453	chr9	12076041	12076453	+	Intergenic	AluSx3|SINE|Alu	-617139	NM_000550	7306	Hs.270279	NM_000550	ENSG00000107165	TYRP1	CAS2|CATB|GP75|OCA3|TRP|TRP1|TYRP|b-PROTEIN	tyrosinase related protein 1	protein-coding
chr1.193210935.193211341	chr1	193210936	193211341	+	intron (NM_024529, intron 15 of 16)	L1MA4A|LINE|L1	-55395	NM_003783	8707	Hs.518834	NM_003783	ENSG00000162630	B3GALT2	BETA3GALT2|GLCT2|beta3Gal-T2	beta-1,3-galactosyltransferase 2	protein-coding
chr8.76097903.76098331	chr8	76097904	76098331	+	Intergenic	L1PA16|LINE|L1	92599	NR_103848	101805492	Hs.571424	NR_103848		CASC9	ESCCAL-1|ESSCAL1|LINC00981|linc-JPH1	cancer susceptibility 9	ncRNA
chr9.76113723.76114190	chr9	76113724	76114190	+	Intergenic	LTR55|LTR|ERV	347310	NM_000700	301	Hs.494173	NM_000700	ENSG00000135046	ANXA1	ANX1|LPC1	annexin A1	protein-coding
chr6.80175922.80176375	chr6	80175923	80176375	+	Intergenic	Intergenic	70998	NM_181714	167691	Hs.21945	NM_181714	ENSG00000135338	LCA5	C6orf152	lebercilin LCA5	protein-coding
chr2.208221396.208221731	chr2	208221397	208221731	+	Intergenic	Intergenic	-87416	NR_031633	100302130		NR_031633	ENSG00000221628	MIR1302-4	MIRN1302-4|hsa-mir-1302-4	microRNA 1302-4	ncRNA
chr6.146437151.146437546	chr6	146437152	146437546	+	intron (NM_001278065, intron 2 of 9)	AluSz6|SINE|Alu	87015	NM_001278064	2911	Hs.32945	NM_000838	ENSG00000152822	GRM1	GPRC1A|MGLU1|MGLUR1|PPP1R85|SCA44|SCAR13	glutamate metabotropic receptor 1	protein-coding
chr1.215618423.215618744	chr1	215618424	215618744	+	Intergenic	L1PA13|LINE|L1	-122138	NM_001319295	51133	Hs.335139	NM_016121	ENSG00000136636	KCTD3	NY-REN-45	potassium channel tetramerization domain containing 3	protein-coding
chr12.87712445.87712802	chr12	87712446	87712802	+	Intergenic	AluSx|SINE|Alu	-11892	NR_135020	105369879	Hs.552351	NR_135020		LOC105369879	-	uncharacterized LOC105369879	ncRNA
chr12.75013379.75013952	chr12	75013380	75013952	+	Intergenic	ERVL-int|LTR|ERVL	82115	NM_001136262	552889	Hs.744849	NM_001136262	ENSG00000253719	ATXN7L3B	lnc-SCA7	ataxin 7 like 3B	protein-coding
chr8.55238258.55238926	chr8	55238259	55238926	+	Intergenic	Intergenic	-131903	NM_022454	64321	Hs.98367	NM_022454	ENSG00000164736	SOX17	VUR3	SRY-box transcription factor 17	protein-coding
chr6.135746167.135746578	chr6	135746168	135746578	+	intron (NM_001134830, intron 18 of 26)	intron (NM_001134830, intron 18 of 26)	72530	NM_001134832	54806	Hs.386684	NM_017651	ENSG00000135541	AHI1	AHI-1|JBTS3|ORF1|dJ71N10.1	Abelson helper integration site 1	protein-coding
**6 regions that were present only in all 12,13-diHOME-treated samples but absent in all vehicle-treated samples**																
PeakID (cmd=annotatePeaks.pl gained_sites_strict_6_051021.bed hg19)	Chr	Start	End	Strand	Annotation	Detailed Annotation	Distance to TSS	Nearest PromoterID	Entrez ID	Nearest Unigene	Nearest Refseq	Nearest Ensembl	Gene Name	Gene Alias	Gene Description	Gene Type
chr20.36020582.36021041	chr20	36020583	36021041	+	intron (NM_198291, intron 5 of 13)	intron (NM_198291, intron 5 of 13)	46281	NM_198291	6714	Hs.195659	NM_005417	ENSG00000197122	SRC	ASV|SRC1|THC6|c-SRC|p60-Src	SRC proto-oncogene, non-receptor tyrosine kinase	protein-coding
chr3.112590680.112591047	chr3	112590681	112591047	+	Intergenic	L1MA3|LINE|L1	-26067	NM_001199215	344807	Hs.531814	NM_001008784	ENSG00000206531	CD200R1L	CD200R2|CD200RLa	CD200 receptor 1 like	protein-coding
chr5.103681102.103681432	chr5	103681103	103681432	+	Intergenic	Intergenic	-753908	NR_000039	9366	Hs.158296	NR_000039	ENSG00000232159	RAB9BP1	RAB9P1	RAB9B, member RAS oncogene family pseudogene 1	pseudo
chr18.52606861.52607240	chr18	52606862	52607240	+	intron (NM_001143829, intron 4 of 11)	intron (NM_001143829, intron 4 of 11)	18228	NM_001143829	80323	Hs.120790	NM_025214	ENSG00000166510	CCDC68	SE57-1	coiled-coil domain containing 68	protein-coding
chr3.85410057.85410335	chr3	85410058	85410335	+	intron (NM_001167674, intron 1 of 9)	intron (NM_001167674, intron 1 of 9)	-24664	NR_049870	100847077		NR_049870	ENSG00000264084	MIR5688	-	microRNA 5688	ncRNA
chr4.190653046.190653346	chr4	190653047	190653346	+	Intergenic	L2c|LINE|L2	54227	NR_135513	105379514	Hs.543309	NR_135513		LOC105379514	-	uncharacterized LOC105379514	ncRNA

Table.S14

Donor	Treatment	IgE (ng/mL)	IgG (ng/mL)	E_over_G	IgE_IgG ratio
X	Vehicle	0.731	102.91	0.00710329	0.87773289
X	f171	0.701	54.38	0.01289077	1.592873871
X	f171_diHOME	0.835	56.99	0.01465169	1.810466079
X	Peanut	0.733	67.5	0.01085926	1.341846308
X	Peanut_diHOME	0.802	58.36	0.01374229	1.698093731
X	Vehicle	0.477	52.52	0.00908225	1.12226711
X	f171	0.639	42.43	0.0150601	1.860931555
X	f171_diHOME	0.656	46.87	0.01399616	1.729463734
X	Peanut	0.627	52.52	0.01193831	1.475181295
X	Peanut_diHOME	0.641	43.37	0.0147798	1.826296055
Y	Vehicle	0.722	86.33	0.00836326	1.263765093
Y	f171	0.687	94.68	0.00725602	1.0964514
Y	f171_diHOME	0.847	72.57	0.01167149	1.763669424
Y	Peanut	0.789	93.68	0.00842229	1.272685276
Y	Peanut_diHOME	0.848	77.57	0.01093206	1.651935018
Y	Vehicle	0.427	87.64	0.0048722	0.736234907
Y	f171	0.711	62.72	0.0113361	1.712988509
Y	f171_diHOME	0.848	59.22	0.01431949	2.163806135
Y	Peanut	0.603	82.76	0.00728613	1.101001039
Y	Peanut_diHOME	0.634	67.77	0.00935517	1.413652517

Note

1. f171 is the peanut protein.

2. diHOME is short for 12,13-diHOME.

3. peanut is short for peanut extract.

Treat group	Treat
A	Vehicle
B	f171
C	f171_diHOME
D	Peanut
E	Peanut_diHOME

Fig.S3E

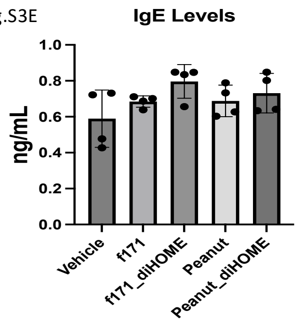

Fig.S3F

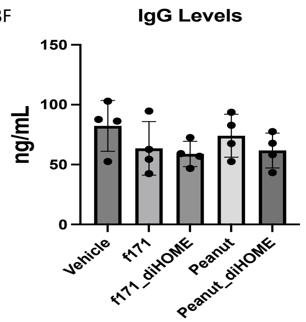

IgE/IgG rato

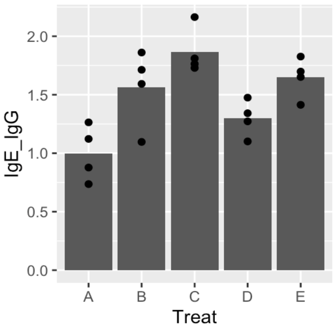

Fig.2F

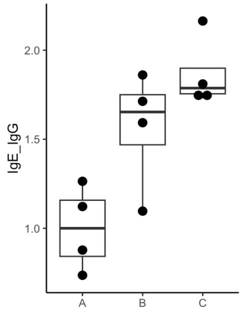

Fig.S3B

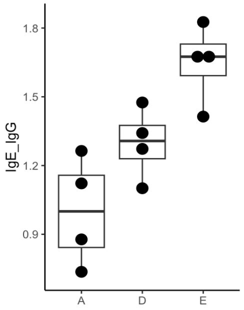

Supplement: Supplementary 2 — Table 1: list of qRT-PCR primers. Table 2: linear mixed model analyses of time-course qRT-PCR. Table 3: differentially expressed genes in primary human macrophages after 4 hr treatment with 12,13-diHOME. Table 4: differentially expressed genes in primary human macrophages after 8 hr treatment with 12,13-diHOME. Table 5: differentially expressed genes in primary human macrophages after 8 hr treatment with combinatorial 12,13-diHOME and peanut. Table 6: enriched gene set enrichment analysis (GSEA) pathways in primary human macrophages after treatment with 12,13-diHOME. Table 7: enriched gene set enrichment analysis (GSEA) pathways in primary human macrophages after treatment with combinatorial 12,13-diHOME and peanut. Table 8: differentially expressed genes in murine airway cells after treatment with E. coli-expressing 3-epoxide hydrolase. Table 9: differentially expressed genes in murine airway cells after treatment with cockroach antigen. Table 10: differentially expressed genes in murine airway cells after treatment with combinatorial cockroach antigen and E. coli-expressing 3-epoxide hydrolase. Table 11: enriched gene set enrichment analysis (GSEA) pathways in murine airway cells after treatment with cockroach antigen. Table 12: enriched gene set enrichment analysis (GSEA) pathways in murine airway cells after treatment with combinatorial cockroach antigen and E. coli-expressing 3-epoxide hydrolase. Table 13: detailed ATAC-seq genomic annotations for 49 differentially accessible chromatin regions. Table 14: human IgE ELISA and IgG CBA data from the ex vivo coculture assay. [file 2506586.f2.pdf]
